# Family-based cognitive behavioural therapy versus family-based relaxation therapy for obsessive-compulsive disorder in children and adolescents: protocol for a randomised clinical trial (the TECTO trial)

**DOI:** 10.1186/s12888-021-03669-2

**Published:** 2022-03-19

**Authors:** Anne Katrine Pagsberg, Camilla Uhre, Valdemar Uhre, Linea Pretzmann, Sofie Heidenheim Christensen, Christine Thoustrup, Iben Clemmesen, Amanda Aaen Gudmandsen, Nicoline Løcke Jepsen Korsbjerg, Anna-Rosa Cecilie Mora-Jensen, Melanie Ritter, Emilie D. Thorsen, Klara Sofie Vangstrup Halberg, Birgitte Bugge, Nina Staal, Helga Kristensen Ingstrup, Birgitte Borgbjerg Moltke, Anne Murphy Kloster, Pernille Juul Zoega, Marie Sommer Mikkelsen, Gitte Sommer Harboe, Katrin Frimann Larsen, Line Katrine Harder Clemmesen, Jane Lindschou, Janus Christian Jakobsen, Janus Engstrøm, Christian Gluud, Hartwig Roman Siebner, Per Hove Thomsen, Katja Hybel, Frank Verhulst, Pia Jeppesen, Jens Richardt Møllegaard Jepsen, Signe Vangkilde, Markus Harboe Olsen, Julie Hagstrøm, Nicole Nadine Lønfeldt, Kerstin Jessica Plessen

**Affiliations:** 1 Child and Adolescent Mental Health Center, Copenhagen University Hospital – Mental Health Services CPH, Gentofte Hospitalsvej 3A, 1. sal, 2900 Hellerup, Copenhagen, Denmark; 2 Department of Clinical Medicine, Faculty of Health and Medical Sciences, University of Copenhagen, Copenhagen, Denmark; 3 Danish Research Centre for Magnetic Resonance, Centre for Functional and Diagnostic Imaging and Research, Copenhagen University Hospital - Amager and Hvidovre, Copenhagen, Denmark; 4 Applied Mathematics and Computer Science, Technical University of Denmark, Kgs Lyngby, Denmark; 5 Copenhagen Trial Unit, Centre for Clinical Intervention Research, Capital Region of Denmark, Rigshospitalet, Copenhagen University Hospital, Copenhagen, Denmark; 6 Department of Regional Health Research, Faculty of Health Sciences, University of Southern Denmark, Odense, Denmark; 7 Department of Neurology, Copenhagen University Hospital Bispebjerg and Fredriksberg, Copenhagen, Denmark; 8 Department of Child and Adolescent Psychiatry, Aarhus University Hospital, Psychiatry, Copenhagen, Denmark; 9 Child and Adolescent Psychiatric Department, Region Zealand Psychiatry, Research Unit, Roskilde, Denmark; 10 Center for Clinical Intervention and Neuropsychiatric Schizophrenia Research (CINS), Mental Health Center Glostrup, Copenhagen University Hospital, Glostrup, Denmark; 11 Department of Psychology, Faculty Social Sciences, University of Copenhagen, Copenhagen, Denmark; 12 Department of Neuroanaesthesiology, The Neuroscience Centre, The Neuroscience Centre, Copenhagen University Hospital – Rigshospitalet, Copenhagen, Denmark; 13 Division of Child and Adolescent Psychiatry, Department of Psychiatry, Lausanne University Hospital (CHUV) and University of Lausanne, Lausanne, Switzerland

**Keywords:** Obsessive-compulsive disorder, Children, Adolescents, Youth, Cognitive behavioural therapy, Psycho-education and relaxation training, Randomised clinical trial, Treatment effects

## Abstract

**Background:**

Cognitive behavioural therapy (CBT) is the recommended first-line treatment for children and adolescents with obsessive-compulsive disorder (OCD), but evidence concerning treatment-specific benefits and harms compared with other interventions is limited. Furthermore, high risk-of-bias in most trials prevent firm conclusions regarding the efficacy of CBT. We investigate the benefits and harms of family-based CBT (FCBT) versus family-based psychoeducation and relaxation training (FPRT) in youth with OCD in a trial designed to reduce risk-of-bias.

**Methods:**

This is an investigator-initiated, independently funded, single-centre, parallel group superiority randomised clinical trial (RCT). Outcome assessors, data managers, statisticians, and conclusion drawers are blinded. From child and adolescent mental health services we include patients aged 8–17 years with a primary OCD diagnosis and an entry score of ≥16 on the Children’s Yale-Brown Obsessive-Compulsive Scale (CY-BOCS). We exclude patients with comorbid illness contraindicating trial participation; intelligence quotient < 70; or treatment with CBT, PRT, antidepressant or antipsychotic medication within the last 6 months prior to trial entry. Participants are randomised 1:1 to the experimental intervention (FCBT) versus the control intervention (FPRT) each consisting of 14 75-min sessions. All therapists deliver both interventions. Follow-up assessments occur in week 4, 8 and 16 (end-of-treatment). The primary outcome is OCD symptom severity assessed with CY-BOCS at end-of-trial. Secondary outcomes are quality-of-life and adverse events. Based on sample size estimation, a minimum of 128 participants (64 in each intervention group) are included.

**Discussion:**

In our trial design we aim to reduce risk-of-bias, enhance generalisability, and broaden the outcome measures by: 1) conducting an investigator-initiated, independently funded RCT; 2) blinding investigators; 3) investigating a representative sample of OCD patients; 3) using an active control intervention (FPRT) to tease apart general and specific therapy effects; 4) using equal dosing of interventions and therapist supervision in both intervention groups; 5) having therapists perform both interventions decided by randomisation; 6) rating fidelity of both interventions; 7) assessing a broad range of benefits and harms with repeated measures.

The primary study limitations are the risk of missing data and the inability to blind participants and therapists to the intervention.

**Trial registration:**

ClinicalTrials.gov: NCT03595098, registered July 23, 2018.

**Supplementary Information:**

The online version contains supplementary material available at 10.1186/s12888-021-03669-2.

## Background

Obsessive-compulsive disorder (OCD) affects 0.5 to 3% of children and adolescents in the population [1] and is associated with reduced quality of life and significant social and occupational impairment [2]. In Denmark, a recent study showed that the cumulative incidence rate of OCD in children (< age 18 years) was higher for girls, 0.96% [95% CI, 0.92–1.00%], than for boys 0.63%, [95% CI, 0.56–0.72%] [3]. OCD is characterised by persistent intrusive thoughts, urges, or images that cause anxiety (obsessions), and/or by repetitive behaviours (compulsions) that are performed in an attempt to reduce anxiety or discomfort [4]. Early detection and intervention is important to ensure a good prognosis, as the disorder often persists into adulthood and can become chronic if left untreated [5, 6].

The recommended first-line treatment for youth with OCD (age < 18 years) is behavioural therapy or cognitive behavioural therapy (CBT) either alone or in combination with antidepressant medication in more severely affected cases [7–9]. Yet, more than 40% of patients do not or only partially benefit from CBT. The cornerstone of CBT for OCD is exposure and response prevention (ERP), in which patients are gradually exposed to anxiety provoking situations that trigger obsessions and then encouraged to refrain from compulsive behaviour. Our recent systematic review showed that CBT may be an effective treatment for OCD in youths, but the included trials were at high risk-of-bias and the certainty of the evidence was low [10]. Also, information about effects on outcomes other than symptom severity was limited [10]. While symptom reduction represents an important outcome, outcomes such as adverse events, quality of life, and daily life functioning are equally relevant [10].

The efficacy of CBT for children and adolescents with OCD has been compared with credible control interventions such as relaxation training (RT) or psychoeducation and relaxation training (PRT) in three randomised clinical trials (RCTs), all pointing to the superiority of CBT [11–13]. Response rates in the three trials were 50 to 72% for CBT versus 20 to 41% for PRT with an effect size of 0.3 reported in one of the studies [12]. However, these trials were at risk-of-bias due to unclear randomisation process, missing outcome data and, for one trial, deviations from the intended treatment [14]. Also, although one study found higher response rates and a faster decline in OCD severity with CBT compared to PRT, symptom reduction at end of treatment was comparable in the two groups [13].

While drop-out rates from CBT of up to 26% implies some degree of unacceptability of the treatment [15], adverse events or reactions are not systematically monitored or reported in psychotherapy trials [16]. One study reported that psychotherapists within child- and adolescent psychiatric services in Sweden were unfamiliar with the concept of adverse events in psychotherapy [17]. Current estimates of how frequently adverse events occur in psychotherapy are based on surveys that ask either therapists or patients to evaluate negative therapy outcomes in retrospect. For example, 5.2% of patients reported lasting harmful effects from psychotherapy in a British survey [18].

To improve our understanding of the treatment effects of CBT in children and adolescents with OCD there is a need for a carefully designed RCT at low risk-of-bias, which specifically addresses the broader treatment effects as well as tolerability.

The TECTO trial aims to compare the benefits and harms of family-based CBT (FCBT) versus family-based psychoeducation and relaxation training (FPRT) in children and adolescents with OCD to guide future clinical practice and research. We include an active intervention as comparison to tease apart general and specific therapy effects and allow us to investigate possible predictors, moderators, and mediators of CBT.

The null hypothesis of this superiority trial is that both interventions have similar therapeutic effects for the outcomes of interest. The alternative hypothesis is that FCBT will be superior to FPRT in alleviating OCD symptoms and improving health-related quality of life, and the co-primary alternative hypothesis is that FCBT will be associated with more adverse events than FPRT due to the ERP component of the FCBT.

## Methods

### Design

**Fig. 1:**
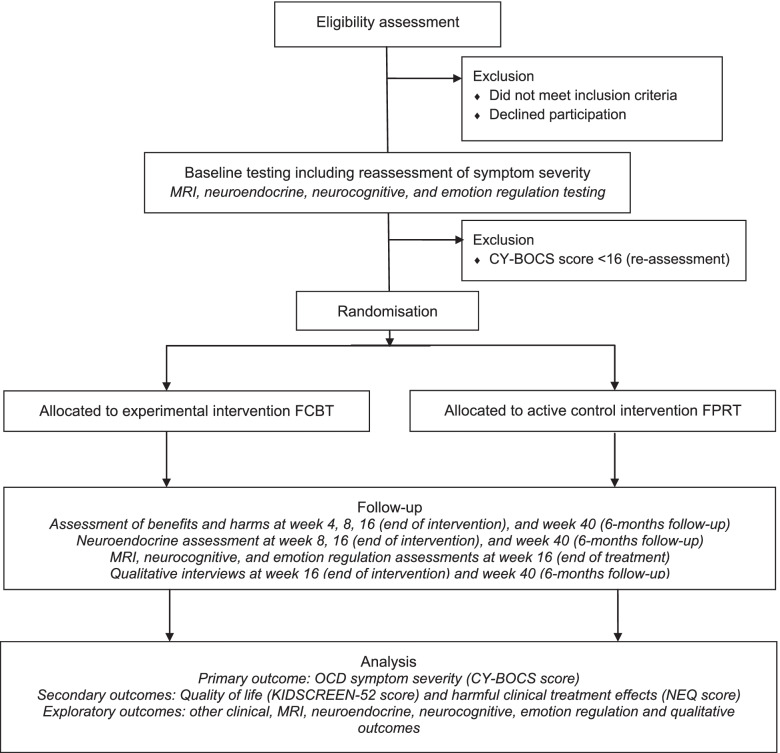
TECTO flow diagram

The TECTO trial is an investigator-initiated, independently funded, single-centre, parallel group, randomised superiority clinical trial in a hospital setting comparing 16 weeks of FCBT versus FPRT in children and adolescents with OCD aged 8 to 17 years (both inclusive). This design allows us to test how CBT-specific factors (e.g. the ERP component) contribute to the observed treatment effects. A follow-up assessment is conducted 6 months after end of treatment to investigate the stability of treatment outcomes. The TECTO trial protocol follows the SPIRIT recommendations [19] and has been registered at clinicaltrials.gov (NCT03595098, 23 July 2018, final update is protocol version 13.0, 11 June 2021). Figure 1 shows the TECTO flow diagram and the populated SPIRIT checklist is provided in Supplementary file 1.

### Sub-studies

We combine the TECTO RCT with longitudinal case-control sub-studies to elucidate how neurobiological, cognitive, emotional, and neuroendocrine factors may predict, moderate and mediate CBT responses. The sub-studies involve neuroimaging of brain structure and function, evaluation of therapy factors (such as patient and parent treatment confidence, motivation, alliance, and compliance, and therapist fidelity to manuals), as well as tests of neurocognitive functions across domains, emotion regulation, and salivary oxytocin levels. Analysis of the TECTO trial data will be conducted in three steps. Step 1 is the main analysis of the RCT presented here, in which we test the efficacy of FCBT versus FPRT. In Step 2, we test sub-study-specific hypotheses and extract features for Step 3. In Step 3, we integrate data using machine learning techniques (see e.g. [20]) to investigate which multivariate combinations of features (e.g. brain activity patterns; clinical, therapeutic and family factors; cognitive and emotion regulation measures; and oxytocin levels) best predict treatment outcomes and differentiate between patients and healthy controls, and between treatment responders and non-responders among patients. Finally, we conduct a separate sub-study involving both quantitative and qualitative methods to examine which and how adverse events are related to psychotherapy for youth with OCD.

The sub-studies as well as the six-month follow-up study will not be presented in further detail in the present paper (but they are detailed including plans for collection, laboratory evaluation, and storage of biological specimens in NCT03595098 on clinicaltrials.gov).

### Setting

The TECTO trial is conducted at the Child and Adolescent Mental Health Center (CAMHC), Copenhagen University Hospital – Mental Health Services CPH, Denmark. CAMHC is a free-of-charge public healthcare provider for children and adolescents below age 18 years. 1.8 million people live in the Capital Region of Denmark of whom around 200,000 are in the target age group of the TECTO trial. The sample will be representative of the clinical population of youth with moderate to severe OCD, as only a limited capacity of non-hospital mental health services exists in Denmark. All individuals with suspected OCD aged 8 to 17 years are directly referred by the Central Visitation Unit to our OCD team, which is established to promote clinical expertise and research in the management of OCD at CAMHC. In addition, we facilitate referral of patients with suspected OCD from 1) the Tourette Clinic at the Department of Pediatric and Adolescent Medicine, Herlev Hospital, Capital Region; 2) Pedagogical Psychological Services in the 29 municipalities of the Capital Region; and 3) general practitioners and physicians from non-hospital child and adolescent psychiatric clinics in the Capital Region. The TECTO trial organization is shown in supplementary file 2.

### Participants

#### Inclusion and exclusion criteria

Inclusion criteria

- OCD as primary diagnosis, meeting the criteria for ICD-10 F42 [4], based on a semi-structured psychopathological interview using the Kiddie-Schedule for Affective Disorders and Schizophrenia - Present and Lifetime Version (K-SADS-PL) [21].
- Children’s Yale-Brown Obsessive-Compulsive Scale (CY-BOCS) [22] entry score ≥ 16, a cut-off score used in previous studies [13, 23].
- Ages 8 through 17 years (both inclusive).
- Signed informed consent.

Exclusion criteria

- Comorbid illness that contraindicates trial participation: pervasive developmental disorder excluding Asperger’s syndrome (ICD-10 F84.0–84.4 + F84.8–84.9); schizophrenia/paranoid psychosis (ICD-10 F20–25 + F28–29); mania or bipolar disorder (ICD-10 F30 and F31); depressive psychotic disorders (F32.3 + F33.3); substance dependence syndrome (ICD-10 F1x.2) [4].
- Intelligence quotient < 70 measured with the full scale Wechsler Intelligence Scales (either WISC-V [24] for children ages 8 to 16 years or WAIS-IV [25] for adolescents aged 17 years).
- Treatment with CBT, PRT, antidepressant or antipsychotic medication within the last 6 months prior to trial entry.

#### Recruitment procedure, eligibility screening, and baseline assessment

Based on the standard clinical assessment, our specialised OCD team evaluates whether a patient is eligible for participation in the trial. All diagnostic evaluations are based on the structured psychopathological interview (K-SADS-PL) and confirmed by a consultant or a specialised psychologist in child and adolescent psychiatry. If the patient meets the criteria, the family members receive age-appropriate verbal and written information about the trial (for details, please see supplementary file 3 and 4).

If the parents or legal caretaker gives informed consent to study participation, we collect baseline data. In addition to patient medical history, clinical and diagnostic evaluation, and somatic examination, the assessment includes the CY-BOCS, a semi-structured interview assessing the severity of OCD symptomatology [22]; the Wechsler Intelligence Scales (WISC-V or WAIS-IV depending on the age of the participant [24, 25]); and the Social Responsiveness Scale (SRS) (a parent and/or teacher rating scale assessing the presence and extent of social and communicative impairment) [26]. If the period between screening and start of treatment exceeds 1 week, we perform a new baseline CY-BOCS before randomisation.

Trial participants are compensated with a DKK 250 gift card per test day for engaging in research activities that go beyond the standard assessment and treatment programme.

#### Withdrawal/discontinuation from trial

Participants who no longer wish to participate in the trial can withdraw their informed consent at any time without explaining the reason and with no consequences for the participant’s further treatment. We discontinue participants from the intervention if the participant experiences intolerable adverse reactions, shows symptoms contraindicating further trial participation, is diagnosed with any disorder that is defined as an exclusion criterion during the intervention period, or experiences a significant worsening of their clinical state during the course of the trial (i.e. increases of 30% or more from baseline on the CY-BOCS total score). In all cases of discontinuation, the investigator and/or therapist will encourage the participant to continue with follow-up assessment and collected data will be used in analyses. Reasons for withdrawal or discontinuation are systematically documented.

#### Risks and benefits for participants

We are not aware of any major risks or safety issues associated with participation in the trial. We expect most patients to benefit from both interventions. We hypothesise that some patients in both intervention groups may experience anxiety symptoms or lack of improvement. All procedures of the trial have been designed with careful consideration of our participants being vulnerable children and adolescents. We believe that any potential inconvenience caused by trial participation can be justified by the potential scientific value of our results, leading to improved treatment options for youth with OCD. CAMHC provides care for participants who need more treatment after receiving psychotherapy in the TECTO trial.

### Parental participation

Parents or caretakers of children with OCD are often involved in the child’s symptoms which may negatively affect the functional level of the family [27]. Thus, parental training is important to increase the effectiveness of psychotherapy [28]. Therefore, we include parents as participants. The parents are involved in the clinical assessments and treatment of their child. We observe and score parent-child interactions in clinically relevant situations (e.g. in the presence of a feared stimulus, and in an emotion regulation task) [29, 30]. If the parents do not give informed consent to be trial participants, the child can still be included.

### Processes

#### Trial conduct

The trial is conducted in compliance with the study protocol, the Helsinki Declaration [31], and the applicable regulatory requirements (The Ethics Committee of Capital Region of Denmark approval number: H-18010607, and The Knowledge Centre on Data Protection Compliance in The Capital Region of Denmark: VD-2018-263, I-Suite no.: 6502). We act in accordance with the Danish personal and health data regulations when collecting information from patients’ medical records (The Danish Act on Processing of Personal Data, and Danish Health Act, Section 43, Subsection 1). Recruitment of participants started after regulatory approvals was obtained. Recruitment and randomisation of the first participant took place on September 4, 2018, and randomisation of the last participant is expected to take place by the end of 2021. Final follow-up of the last participant (at six-month follow-up) is scheduled for the end of 2022.

Protocol amendments are implemented only after re-approvals from the ethics committee and important protocol modifications (e.g. changes to eligibility criteria, outcomes, analyses) are communicated directly and in collaboration with Copenhagen Trial Unit to relevant parties (e.g. investigators, clinical departments, trial participants, trial registries, steering committee, advisory board).

#### Ethics approval and consent to participate

The Ethics Committee of Capital Region of Denmark approved the protocol (H-18010607). Patients who are deemed eligible to participate in the trial according to the in- and exclusion criteria receive verbal (all ages) and written (adolescents aged at least 15 years and all parents/legal guardians) information about the trial and are informed of their rights to withdraw from the trial at any point without it affecting future treatment. All participants and their guardians are given verbal information about the trial by a health care professional in the outpatient clinic, OCD-team, and are asked permission to be contacted by the research team. Initial information about the trial is provided in an age-appropriate manner and during regular clinic visits at the CAMHS in the presence of a guardian and in the privacy of an examination room. Guardians are explained their right to have an assessor (e.g. friend or family member) present and in case the guardian should want that, a new appointment will be made for the information meeting. Written information brochures approved by the ethical committee explaining the study background, procedures and aims are handed out to all potential participants and their guardians. Potential participants then have a minimum of 24 h to consider participation before being contacted by the doctor, psychologist, or the trial manager involved in the trial. If potential participants and their guardians approve to participate, both guardians sign informed consent at the first contact. Guardians have the possibility of signing a power of attorney to the other guardian. Guardians are informed that use of this form is voluntary and can be withdrawn at any time. Furthermore, if a participant turns 18 years old before the end of the trial, the participant is be asked to sign an informed consent at trial start. Each guardian receives their own participant information (verbal and written) and informed consent form regarding parental participation (for details, please see supplementary file 3 and 4).

#### Randomisation

Participants are randomised at the allocation ratio 1:1. Randomisation is handled centrally at an external unit, the Copenhagen Trial Unit, using a computer-generated allocation sequence with varying block sizes concealed from the investigators. The allocation sequence is stratified by age (8 to 12 years and 13 to 17 years) and CY-BOCS total score at baseline (16 to 23 points (moderate severity) and 24 to 40 points (severe to extreme severity)). Participants are enrolled and assigned to the intervention groups using a web-based system developed by the Copenhagen Trial Unit.

#### Blinding

We employ blinding to the intervention whenever possible. It is not possible to fully blind the participants, their parents, and the therapists due to the explicit nature of the intervention. However, the name and the specific content of the assigned intervention is not disclosed to participants and their parents. Outcome assessment is performed by blinded investigators. Data managers, statisticians, and conclusion drawers are fully blinded as well. Before the follow-up assessment sessions are conducted during the trial, unblinded trial personnel instruct the child/families to avoid giving any information concerning the therapy to the blinded outcome assessor. We will follow the rule that statistical analyses are conducted with the intervention groups coded as e.g. ‘Intervention A’ and ‘Intervention B’. We will write two abstracts while the blinding is intact: one assuming the experimental intervention group is A and the control intervention group is B, and one assuming the opposite. After this, the code will be broken.

Investigators doing qualitative interviews will be unblinded and do no further assessment of the participant after the interview. Participants leaving the trial can be unblinded if they wish to. Unblinding for the entire trial cohort will be performed confidentially via the data manager to the steering committee after the two conclusions have been drawn.

#### Participant timeline

Both intervention groups involve therapy delivered over 16 weeks. Participants undergo assessments at baseline (week 0–1), at week 4, week 8, and at end-of-treatment (week 16). A long-term follow-up takes place at week 40. Table 1 shows the participant timeline and outcome assessments.

**Table 1:** Participant timeline and outcome assessments in the 16-week TECTO trial and at the week-40 follow-up

	week													
Activities/assessment	0	1	2	3	4	5	6	7	8	9–13	14	15	16	40
Intervention		x	x	x	x	x	x	x	x	x	x	x	x	
**Measures**														
Clinical state (CY-BOCS, KIDSCREEN, COIS*, CGI-S, CGI-I**)	x				x				x				x	x
C-GAS	x												x	
Self-rated obsessive-compulsive traits	x												x	
Diagnostics (K-SADS-PL) including suicidality	x												x	
Intelligence (WISC-V/WAIS-IV)	x													
Social competences (SRS)	x													
Negative effects of psychotherapy (NEQ)					x				x				x	x
Parental Stress (PSS), Family accommodation (FAS)	x				x				x				x	x
Family Environment (FES)*	x													
Confidence in treatment		x												
Motivation for treatment		x			x				x		x			
Treatment compliance		x	x	x	x	x	x	x	x	x	x	x	x	
Therapeutic alliance (TASC-R)		x			x				x				x	

* Self-reported from age: 11 years. Parent-reported from age: 8–17 years, ** Not at assessed at week 0

*CGAS* The Children’s Global Assessment Scale; *CGI-S/I* The Clinical Global Impression Scale - severity/improvement; *COIS* Child Obsessive Compulsive Disorder Impact Scale; *CY-BOCS* Children’s Yale-Brown Obsessive-Compulsive Scale; *FAS* Family Accommodation Scale; *FES* Family Environment Scale; *K-SADS-PL* Kiddie-Schedule for Affective Disorders and Schizophrenia; *NEQ* Negative Effects Questionnaire; *PSS* Parental Stress Scale; *SRS* Social Responsiveness Scale; *TASC-R* Therapeutic Alliance Scale for Children; *TOCS* Toronto Obsessive-Compulsive Rating Scale; *WAIS-IV* The Wechsler Adult Intelligence Scale; *WISC-V* The Wechsler Intelligence Scale for Children. Note: assessments for sub-studies (MRI, neuroendocrine, neurocognitive, emotion regulation and qualitative interviews) are not stated here.

#### Outcomes

The primary outcome is OCD symptom severity assessed with the CY-BOCS at the end of intervention. Secondary outcomes are 1) health-related quality of life assessed with the Health-related Quality of Life Screening Instrument for Children and Adolescents (KIDSCREEN-52) [32] at the end of intervention; and 2) adverse events during the intervention, assessed with the Negative Effects Questionnaire (NEQ), which measures six factors; symptoms, quality, dependency, stigma, hopelessness, and failure.

Exploratory outcomes are: serious adverse events (SAE) (assessed until week 40); Child Obsessive-Compulsive Impact Scale (COIS) [33]; Clinical Global Impression – Severity and Improvement (CGI-S and CGI-I) [34]; Children’s Global Assessment Scale (C-GAS) [35]; diagnostic status and proportion of patients in remission (no longer meeting the diagnostic criteria for OCD (ICD-10 F.42)), assessed with K-SADS-PL [21] at the end of the intervention; response defined as a reduction on the CY-BOCS at end-of-treatment of at least 30% in intraindividual comparison with the score at baseline; Toronto Obsessive-Compulsive Rating Scale (TOCS) [36]; suicidality (K-SADS-PL suicidality items sum-score); the Family Accommodation Scale (FAS) [37], a parent-reported measure that examines parental accommodation to children’s obsessions and compulsions; and the Parental Stress Scale (PSS) [38], a measure of perceived stress pertaining to the parenting role. Finally, we will assess social and environmental characteristics of families with the Family Environment Scale (FES) [39].

Therapy factors, such as confidence in treatment (on a 7-point Likert scale), motivation for treatment (on a 7-point Likert scale), the Therapeutic Alliance Scale for Children–revised (TASC-R) [40, 41], and compliance (see below) will be assessed in exploratory analyses as covariates for outcome.

In the 16-week trial, all outcomes are measured at baseline and week 16. In addition, several outcomes are measured repeatedly: clinical state measures (CY-BOCS, KIDSCREEN, COIS and CGI-I/S) along with family factors (PSS and FAS) and adverse events (NEQ) are also assessed at week 4 and week 8. Moreover, treatment compliance is measured at every session and motivation for treatment and therapeutic alliance is assessed at week 4 and week 8.

#### Assessment team

Trained clinicians blind to intervention group (PhD students or psychologists/MDs, and for selected assessments trained and supervised psychology or medical students) perform the assessments. Participating patients and parents fill out the self-administrated questionnaires.

#### Safety

We use the generic definition of adverse events as defined by the International Conference on Harmonization of Technical Requirements for Registration of Pharmaceuticals for Human Use – Guidelines for Good Clinical Practice [42] (see supplementary file 5). All SAE’s will be reported to The Ethics Committee of Capital Region of Denmark.

#### Quality assurance and quality control

Representatives from the Copenhagen Trial Unit monitor activities in accordance with Good Clinical Practices [42] as far as applicable for a non-pharmacological trial. Activities are monitored via on-site visits combined with central (remote) monitoring. In general, a risk-based approach will be taken by defining the intensity of monitoring required and central monitoring and central review of monitoring reports.

### Interventions

The experimental intervention is a manualised form of exposure-based FCBT for OCD [43]. The key components are ERP, family involvement, psychoeducation, and homework assignments. The active control condition is manualised FPRT [13]. The key components are relaxation training (activation and relaxation of individual muscles and muscle groups, breathing exercises), family involvement, psychoeducation, and homework assignments.

Both interventions include 14 sessions each of 75 min, delivered over 16 weeks (weekly sessions at week 1 to 12, and a session at week 14 and one at week 16, with the possibility of a flexible planning of the two session-free weeks). Elements common to both interventions include: the therapeutic approach of externalising OCD; setting an agenda at each session; assigning and reviewing homework; monitoring and ranking symptoms; providing treatment rationale; involving parents; using positive reinforcement (rewards); building a collaborative working alliance; and providing psychoeducation about OCD and the connection between thoughts, emotions, bodily sensations, and behaviours (the cognitive diamond). Parents may assume a supportive role for the child or as a co-therapist. In five of the 14 sessions (sessions 1, 2, 7, 11, and 14) the parents join their child for the entire session. In the remaining sessions, the child is treated individually for 45 min, followed by parent-sessions for an additional 30 min with or without the child present. To be classified as a family-based intervention, at least one parent or legal caretaker must participate in at least three sessions. The participants will be offered a booster session within the first 6 months after the 16-week intervention. Table 2 illustrates similarities and differences between FCBT and FPRT.

**Table 2:** Similarities and differences between FCBT and FPRT in the TECTO trial

FCBT & FPRT		FCBT		FPRT	
**Session**	**Parent Presence in minutes**	**Activities/Topics**	**Parent Themes**	**Activities/ Topics**	**Parent Themes**
**1**	**75**	Establish contact	Role: helper	Establish contact	Establish contact
		Psychoeduation on OCD and FCBT		Psychoeduation on OCD and FPRT	
				Externalizing OCD	
**2**	**75**	Externalizing OCD	Expectations for therapy	Psychoeduation on OCD and FPRT	Expectations for therapy
		Symptom list		Goals for therapy	
		Symptom hierarchy		Symptom hierarchy	
		Homework			
**3**	**30**	Cognitive training	Family beliefs and attitude toward OCD Rewards	Symptom hierarchy	Parent relaxation practice
		Mapping OCD symptoms		Rewards	
		Test ERP		Identify OCD discomfort/distress in body	
				PRT	
**4**	**30**	ERP	Role	Symptom hierarchy	Facilitate PRT for child
		Toolbox	Guilt and blame	PRT	
**5**	**30**	ERP	FA	Symptom hierarchy	Guilt and blame
		Fight against OCD		PRT	
**6**	**30**	ERP	Child’s responsibility for treatment	Symptom hierarchy	Family beliefs and attitude toward OCD and affected child
		Get more control over OCD		PRT	
**7**	**75**	ERP	Role	Symptom hierarchy	PRT practice
		Psychoeduation on OCD and CBT		PRT	
				Goals for therapy	
				Psychoeduation on OCD and FPRT	
**8**	**30**	ERP	Motivation	Symptom hierarchy	Child’s responsibility for treatment
		special therapeutic needs	Obstacles	PRT in new environment	
**9**	**30**	ERP	Differientiate OCD from other problems	Symptom hierarchy	Differientiate OCD from other problems
		Fight against OCD		PRT	
**10**	**30**	ERP	Cohesion	Symptom hierarchy	Cohesion
		Fight against OCD		PRT	Family engagement in treatment
**11**	**75**	ERP	Problem solving	Symptom hierarchy	Problem solving
		Evaluate treatment		Goals for therapy	
				PRT	
**12**	**30**	ERP	Relapse prevention	Symptom hierarchy	Relapse prevention
		Agree on continued focused		PRT	
**13**	**30**	ERP	Relapse prevention	Symptom hierarchy	Relapse prevention
		Relapse prevention		PRT	
**14**	**75**	End of therapy ceremony	Future plans	End of therapy ceremony	Future plans
		Review treatment		Review treatment	evaluation

*FCBT* Family-based Cognitive Behavioural Therapy; *FPRT* Family-based Psychoeducation & Relaxation Training; *OCD* Obsessive compulsive disorder; *ERP* Exposure and Response Prevention

#### Experimental intervention – family based cognitive behavioural therapy

The FCBT manual was published in Danish in 2015 [43], and was used in the The Nordic long-term OCD treatment study (NordLOTS), a large, multicentre, open study covering three Scandinavian countries [23]. It is based on the treatment manuals by March and Mulle [44] as well as an adapted version by Piacentini [13], adding more family-based intervention. Addressing family factors that may influence the treatment response in paediatric OCD is a potential target for optimising exposure-based CBT. In particular, family accommodation (i.e. family members of the patient with OCD participate in rituals and/or modification of routines) appears to constitute a barrier to treatment because it reinforces avoidance behaviours and undermines exposure-based exercises [13, 28]. The key components in FCBT are in-session and at home ERP practice [45].

#### Control intervention – Psychoeducation/relaxation training

The active control intervention is manualised FPRT based on the relaxation manual by Cautela and Groden [46], adapted by Piacentini for use in a previous trial [13] and translated into Danish and adapted for use in the TECTO trial. The sessions consist of psychoeducation, muscle relaxation, attention training, breathing exercises, and visualisation techniques. *Proscribed* interventions include ERP, discouraging compulsive behaviour, discouraging family accommodation, replacing compulsions with relaxation techniques, and positively reinforcing refraining from performing compulsions.

#### Concomitant interventions

Concomitant treatment with any other psychotherapy, antidepressant and antipsychotic medication is not permitted. All other types of concomitant treatments, such as counselling, parent support, network management or in-patient care are allowed provided both intervention groups have equal access.

#### Criteria for modification of interventions for a given trial participant

We strive to perform all 14 sessions of treatment within 16 weeks (maximal duration 18 weeks). For an individual treatment course to be defined as complete, 10 out of the total 14 sessions should be delivered. Breaks in treatment are minimised and reasons for breaks are registered. In the case of adverse events or significant worsening of clinical state, the patient may be discontinued from the intervention by the investigator and continue in treatment as usual in the clinic.

#### Assessment of participant compliance

During the treatment period (weeks 1 to 16), we assess the participants’ and parents’ compliance to therapy on a weekly basis. Compliance is assessed by the therapist and includes measures of patient and parent attendance and homework compliance.

#### Therapists

Each therapist conducts both interventions. To avoid potential ‘treatment-by-therapist-confounding’, we balance the assignment of the clinical therapists over time as part of the randomisation process. Both interventions are carried out by master’s level clinical therapists who are either psychologists or child and adolescent consultant psychiatrists with comprehensive post-graduate clinical training in cognitive therapeutic techniques. Each therapist receives education and bi-weekly supervision in both interventions by a certified (FCBT) or specially trained (FPRT) supervisor. Before treating any trial participant, therapists are required to treat at least one non-trial patient with FCBT and one with FPRT under live or video recorded observation.

#### Treatment fidelity

All treatment sessions are video recorded if the participant consents to this. To investigate fidelity to the treatment manuals, approximately 15% of all FCBT sessions and FPRT sessions, distributed evenly across the 14 treatment sessions, are randomly selected for adherence and quality review. Fidelity for FCBT is evaluated using the NordLots Treatment Integrity Scale [23, 45] and for FPRT by a corresponding manual developed by the TECTO research team (supplementary file 6). We evaluate both interventions concerning therapeutic alliance, psychoeducation, exposure, relaxation training, and family involvement on three categories of treatment fidelity: 1) manual adherence, 2) treatment differentiation, and 3) therapist competence.

### Statistical analysis

#### Data management

Data management is handled by an external and independent party at the Copenhagen Trial Unit. Data is collected in OpenClinica, an electronic data capture system for clinical trials. All entries are logged in OpenClinica and data validation checks are conducted to obtain a high quality of data. The electronic data capture system and all associated databases follow the regulations set by The Knowledge Centre on Data Protection Compliance in The Capital Region of Denmark and adheres to the General Data Protection Regulation.

#### Sample size estimation and feasibility of recruitment

The sample size is based on the primary outcome, the CY-BOCS score (continuous variable) measuring severity of OCD symptoms on 10 items which can be rated 0 to 4 points (total score range 0 to 40). Using a power of 80%, a two-sided alpha of 5%, and expecting a SD of 8 on the CY-BOCS total score based on reports in similar patient groups [13], the required sample size necessary to detect or reject a minimal relevant difference of at least 4 points on CY-BOCS total score was estimated to be 64 participants in each intervention group, a total of 128 [13, 47]. Power calculations for secondary outcomes (KIDSCREEN-52 and NEQ) will follow in a detailed statistical analysis plan (see below).

To estimate the expected recruitment potential of OCD patients in CAMHC we drew on the available hospital statistics in the planning phase of the trial before initiation in 2018. In the year 2016, 108 patients aged 8–17 years were referred to and treated in CAMHC for OCD. We therefore estimated that around 324 patients would be eligible for participation in the TECTO trial within our recruitment period of 3 years (ultimo 2018 to ultimo 2021). With a target sample size of 128, we considered it feasible to recruit 40% of all referred patients. Randomised clinical trials with psychiatric patients are prone to drop-outs and missing data [48]. Thus, we aim to include and randomise up to 20 extra participants, i.e. up to 148 participants in total (74 in each group), which will increase our power for our primary outcome to 85.7%.

#### Statistical analysis plan

We will analyse all continuous outcomes with linear regression, dichotomous outcomes with logistic regression, and count data with the *van Elteren* test [49]. In the primary analysis, we will include the intention-to-treat population, and the analysis will be adjusted for the stratification variables used in the randomisation. A detailed statistical analysis plan will be developed and published before any analyses are carried out. The analysis plan will include subgroup analysis and handling of missing data.

## Discussion

The TECTO trial is designed to systematically investigate beneficial and adverse effects of FCBT versus FPRT in the treatment of children and adolescents with OCD with as minimised risk-of-bias as we found operational. The main and intended difference between the two treatment approaches is the absence of the ERP component in the FPRT arm of the trial, which is deemed the most effective treatment element for OCD [50, 51]. Several treatment elements of the active control intervention FPRT are specifically designed to mimic traditional FCBT for OCD, thereby providing rigorous control for the non-ERP aspects. In addition to conducting a trial at risk-of-bias with a credible control intervention, the TECTO trial strives to meet the need for systematic and repeated assessment of adverse events and of additional outcomes pertaining to treatment effects beyond symptom reduction.

The American Academy of Child and Adolescent Psychiatry (AACAP) practice parameter recommends CBT as first-line treatment for youth with OCD, emphasizing that families are involved in the treatment of especially younger children with OCD, for whom parents control many aspects of daily activity [9, 52]. Our recent systematic review updated the evidence base for CBT for paediatric OCD and indicated that CBT appears superior to no intervention/placebo and has effects comparable with sertraline [10]. However, the included studies had a high risk-of-bias. Risk-of-bias is an inherent feature of psychotherapeutic interventions which renders the blinding of participants and therapists impossible. In addition, some of the included studies did not conceal allocation, did not blind outcome assessors, or reported incomplete outcome data. These shortcomings resulted in low or very low certainty of the evidence (GRADE) across the evaluated outcomes.

Similar intervention groups as those used in the TECTO trial – CBT versus PRT – with varying degrees of parent involvement have been investigated in three previous RCTs [11–13]. The first trial was published in 2008 and investigated FCBT versus family-based RT (FRT) in 42 young children with OCD aged 5 to 8 years [11]. The intention-to-treat analysis showed a non-significant moderate treatment effect of FCBT, while complete case analysis showed a larger and significant effect. These findings led to a second trial published in 2014 which included 127 participants. This larger trial showed a superiority of FCBT relative to FRT for both primary outcomes: (1) responder status defined as an independent, evaluator-rated CGI-I score of 1 (very much improved) or 2 (much improved) and (2) change in independent evaluator-rated CY-BOCS total score [12]. This trial had a pre-specified sample size, manualised interventions, supervision of therapists, and fidelity ratings in both intervention groups. Furthermore, most comorbidities (except pervasive developmental disorders and Paediatric Autoimmune Neuropsychiatric Disorders Associated with Streptococcal Infections (PANDAS)) were included, strengthening the generalisability to clinical samples. The trial was, however, limited by (1) allowing antidepressant medication at inclusion and during the trial, which may have affected effect sizes; (2) including only outpatients without acute suicidality, which limits generalisability; and (3) not assessing negative effects of treatment (only SAEs were reported).

The third trial was published in 2011 and examined the efficacy of FCBT versus PRT in children and adolescents aged 8 to 17 years with OCD [13]. The 71 patients were randomized 7:3 to 12 sessions of manualized FCBT or PRT. The participants were largely medication-free (8.5% medicated but not with antidepressants) and included OCD patients with comorbidities (except for disorders contraindicating trial participation, including psychosis, pervasive developmental disorders, mania, or substance dependence). Suicidal patients were excluded. FCBT led to significantly higher response rates than PRT in intention-to-treat (57% vs. 27%) and completer analyses (68% vs. 35%). The participants receiving FCBT showed a faster decline in OCD severity during the trial, as compared with those receiving PRT, however, the magnitude of symptom reduction was comparable in the two groups at end point. The trial had careful quality adherence procedures, therapist assignment balanced across conditions, and weekly group supervisions and case reviews for therapists. Therapy sessions were videotaped, and 10% of FCBT sessions were selected and reviewed by experienced CBT therapists and found satisfactory regarding adherence/quality. The trial also had several limitations. No adherence/quality procedure was implemented for the PRT group. Although the trial was well-powered with a randomised design, the somewhat small sample of PRT participants (*n* = 22) combined with quite large effects of PRT may still question whether ERP really is the ‘active ingredient’ in successful OCD treatment. Even though parents attended some full sessions and parts of sessions in the PRT group, there was less parental involvement in PRT than FCBT, and negative effects of treatment were not assessed or reported. Regarding potential conflicts of interest, several of the authors disclosed receiving royalties for the manuals used in the study from Oxford University Press.

We believe to have improved the trial design in TECTO compared to the previous RCTs by planning a sufficiently powered trial with a well-balanced and concealed allocation (1:1). We use repeated measures by assessing several outcomes not only at baseline and end-of-treatment (week 16) but also at week 4 and week 8. Moreover, as part of broadening the spectrum of treatment outcomes TECTO is the first RCT to assess remission for pediatric OCD at end-of treatment according to diagnostic criteria. Another important strength of our trial is the systematic assessment of negative treatment effects throughout the trial.

We further balance the trial groups by providing equal dosing of therapy sessions, parent participation, therapist education and supervision, and fidelity ratings of both interventions. Each therapist will conduct both interventions and we strive to avoid potential ‘treatment-by-therapist-confounding’ by balancing the assignment of the clinical therapists over time. We monitor motivation for treatment and therapeutic alliance repeatedly.

To enhance generalizability, we include patients with a broad range of comorbidities, only excluding patients with conditions contraindicating study participation. Our RCT is the first to include patients with suicidality. Suicidality appears relatively common in paediatric OCD where one small study with 54 patients found 13% with clinically significant suicidal ideation [53]. In adults with OCD, 16 to 63% experience suicidal ideation, with as many as 25% reporting at least one prior suicide attempt [54–56]. To avoid risks of confounding effects, we do not allow concomitant treatment with antidepressant medication.

Both interventions in the TECTO trial are fully manualised. The manual used for FCBT stems from the NordLOTS study, in which the first part of this stepped care study was an uncontrolled clinical trial including 269 participants with OCD aged 7 to 17 years. The study successfully applied an intervention consisting in 14 weekly sessions of FCBT in community mental health clinics, and the response rate among completers was 73% [45]. Patients receiving the therapy had a substantial mean symptom reduction of 53% measured with CY-BOCS, and about half of the participants were in remission at end of treatment. In the TECTO trial, we match our control intervention, FPRT, as closely as possible to the FCBT intervention. The manual used for FPRT stems from Cautela and Groden [46] and is modified by Piacentini, in which patients treated with PRT experienced reduced OCD symptoms, but to a lesser degree than those treated with FCBT [13]. In the trial by Piacentini, participant- and parent-rated confidence in the efficacy of treatment did not differ between PRT and FCBT, further emphasizing that PRT is a credible control treatment [13].

We aim to further minimise risk-of-bias by blinding of outcome assessors, using a random allocation sequence generation through an external unit and performing the trial as an investigator-initiated, independently funded trial. However, we were unable to come up with pragmatic solutions on how to blind the participants, parents and caregivers to the two interventions. The primary trial limitations are the implicit lack of participant and therapist blinding, and the risk of missing data from follow-up assessments.

To conclude, the TECTO trial in an investigator-initiated, independently funded trial using an RCT design with blinded outcome assessment addressing the limitations of former studies of the effects of CBT, which is the recommended first-line treatment for children and adolescents with OCD. We investigate the benefits and harms of FCBT versus FPRT in an optimal trial design including a trial size based on sample size estimation. We aim to minimise risk-of-bias, enhance generalisability, and broaden the outcome measures, several assessed repeatedly. We investigate a representative sample of youth with OCD including suicidal patients, use equal dosing of interventions and equal dosing of therapist supervision in both interventions, have therapists perform both interventions decided by randomisation, perform fidelity ratings of both interventions, and systematically assess both benefits and harms of treatment.

For future perspectives, the TECTO sub-studies, involving specific neurobiological and neurocognitive targets combined with the RCT design presented here, makes it possible to further tease apart CBT-specific and general treatment mechanisms in OCD therapy by including a wide range of neurocognitive and neurobiological outcomes that may predict, moderate or mediate successful treatment. The data from the TECTO RCT forms the basis for our analysis plan for the sub-studies involving testing specific sub-study hypotheses and extraction of features for data integration using machine learning techniques to investigate which multivariate combinations of features best differentiate patients versus healthy controls and treatment responders versus non-responders, and best predict treatment outcomes. The TECTO trial therefore has the potential to document the absolute effect of CBT and suggest concrete mechanisms of change. Finally, the in-depth mixed-method sub-study of adverse events can help inform safer psychotherapy practices, develop instruments and guidelines for monitoring adverse events, and improve patient and parent information regarding expectations and potential risks in psychotherapeutic treatment.

## Supplementary Information

**Additional file 1.**

**Additional file 2.**

**Additional file 3.**

**Additional file 4.**

**Additional file 5.**

**Additional file 6.**

## Data Availability

After the results have been published, we aim to make a depersonalised dataset publically available on, e.g. clinicaltrials.gov, and/or the EU ZENODO database. The final choice will reflect which platform(s) that are compliant with current legislation at that time.

## Abbreviations

AACAP: American Academy of Child and Adolescent Psychiatry
CAMHC: Child and Adolescent Mental Health Centre
CBT: Cognitive Behavioural Therapy
C-GAS: Children’s Global Assessment Scale
CGI-I: Clinical Global Impression-Improvement
CGI-S: Clinical Global Impression-Severity
COIS: Child Obsessive-Compulsive Impact Scale
CY-BOCS: Children’s Yale-Brown Obsessive-Compulsive Scale
ERP: Exposure and Response Prevention
FAS: Family Accommodation Scale
FCBT: Family-based Cognitive Behavioural Therapy
FES: Family Environment Scale
FPRT: Family-based Psychoeducation/Relaxation Training
ICD-10: International Classification of Diseases-10
K-SADS-PL: Kiddie-Schedule for Affective Disorders and Schizophrenia for school-aged children, Present and Lifetime version
NEQ: Negative Effects Questionnaire
OCD: Obsessive-Compulsive Disorder
PRT: Psychoeducation/Relaxation Training
PSS: Parental Stress Scale
RCT: Randomised clinical trial
SAE: Serious Adverse Event
SD: Standard deviation
SRS: Social Responsiveness Scale
TASC-R: Therapeutic Alliance Scale for Children – Revised
TECTO: Treatment Effects of Family Based Cognitive Therapy in Children and Adolescents with Obsessive Compulsive Disorder
TOCS: Toronto Obsessive-Compulsive Scale
WAIS-IV: Wechsler Adult Intelligence Scale, IV
WISC-V: Wechsler Intelligence Scale for Children, V
