# Correction: Family-based cognitive behavioural therapy versus family-based relaxation therapy for obsessive-compulsive disorder in children and adolescents: protocol for a randomised clinical trial (the TECTO trial)

**DOI:** 10.1186/s12888-022-04142-4

**Published:** 2022-07-29

**Authors:** Anne Katrine Pagsberg, Camilla Uhre, Valdemar Uhre, Linea Pretzmann, Sofie Heidenheim Christensen, Christine Thoustrup, Iben Clemmesen, Amanda Aaen Gudmandsen, Nicoline Løcke Jepsen Korsbjerg, Anna-Rosa Cecilie Mora-Jensen, Melanie Ritter, Emilie D. Thorsen, Klara Sofie Vangstrup Halberg, Birgitte Bugge, Nina Staal, Helga Kristensen Ingstrup, Birgitte Borgbjerg Moltke, Anne Murphy Kloster, Pernille Juul Zoega, Marie Sommer Mikkelsen, Gitte Sommer Harboe, Katrin Frimann Larsen, Line Katrine Harder Clemmensen, Jane Lindschou, Janus Christian Jakobsen, Janus Engstrøm, Christian Gluud, Hartwig Roman Siebner, Per Hove Thomsen, Katja Hybel, Frank Verhulst, Pia Jeppesen, Jens Richardt Møllegaard Jepsen, Signe Vangkilde, Markus Harboe Olsen, Julie Hagstrøm, Nicole Nadine Lønfeldt, Kerstin Jessica Plessen

**Affiliations:** 1 Child and Adolescent Mental Health Center, Copenhagen University Hospital – Mental Health Services CPH, Gentofte Hospitalsvej 3A, 1. sal, 2900 Hellerup, Copenhagen, Denmark; 2 Department of Clinical Medicine, Faculty of Health and Medical Sciences, University of Copenhagen, Copenhagen, Denmark; 3 Danish Research Centre for Magnetic Resonance, Centre for Functional and Diagnostic Imaging and Research, Copenhagen University Hospital - Amager and Hvidovre, Copenhagen, Denmark; 4 Applied Mathematics and Computer Science, Technical University of Denmark, Kgs Lyngby, Denmark; 5 Copenhagen Trial Unit, Centre for Clinical Intervention Research, Capital Region of Denmark, Rigshospitalet, Copenhagen University Hospital, Copenhagen, Denmark; 6 Department of Regional Health Research, Faculty of Health Sciences, University of Southern Denmark, Odense, Denmark; 7 Department of Neurology, Copenhagen University Hospital Bispebjerg and Fredriksberg, Copenhagen, Denmark; 8 Department of Child and Adolescent Psychiatry, Aarhus University Hospital, Psychiatry, Copenhagen, Denmark; 9 Child and Adolescent Psychiatric Department, Region Zealand Psychiatry, Research Unit, Roskilde, Denmark; 10 Center for Clinical Intervention and Neuropsychiatric Schizophrenia Research (CINS), Mental Health Center Glostrup, Copenhagen University Hospital, Glostrup, Denmark; 11 Department of Psychology, Faculty Social Sciences, University of Copenhagen, Copenhagen, Denmark; 12 Department of Neuroanaesthesiology, The Neuroscience Centre, The Neuroscience Centre, Copenhagen University Hospital – Rigshospitalet, Copenhagen, Denmark; 13 Department of Psychiatry, Division of Child and Adolescent Psychiatry, Lausanne University Hospital (CHUV) and University of Lausanne, Lausanne, Switzerland

**Correction: BMC Psychiatry 22, 204 (2022)**

**https://doi.org/10.1186/s41205-022-00146-8**

Following publication of the original article [1], the authors identified an error in the author name of Line Katrine Harder Clemmensen.

The incorrect author name is: Line Katrine Harder Clemmesen.

The correct author name is: Line Katrine Harder Clemmensen.

The author group has been updated above and the original article [1] has been corrected.
