# Supplementary material for: Family-based cognitive behavioural therapy versus family-based relaxation therapy for obsessive-compulsive disorder in children and adolescents: protocol for a randomised clinical trial (the TECTO trial)
Source: BMC Psychiatry. 2022 Mar 19;22:204. doi: 10.1186/s12888-021-03669-2 (PMC8933964; doi:10.1186/s12888-021-03669-2)
Supplement: Supplementary file 2 — Additional file 2. [file 12888_2021_3669_MOESM2_ESM.docx]

**Supplementary file 2**

# TECTO trial organisation

## Trial sites

| **Type*** | **Site abbreviation** | **Site details** |
| --- | --- | --- |
| Clinical trial site | CAMHS, Bispebjerg | Child and Adolescent Mental Health Centre (CAMHS), Bispebjerg Bakke 30, 16B 1. sal, 2400 Copenhagen NV, Telephone: +45 38641000, Fax: +45 38641001,  Web: [www.bupsykiatri-bispebjerg.dk](http://www.bupsykiatri-bispebjerg.dk) |
| Trial site | DRCMR, Hvidovre Hospital | Danish Research Centre for Magnetic Resonance Imaging (DRCMR) Unit 714, Hvidovre Hospital, University of Copenhagen, Kettegard Allé 30, 2650 Hvidovre, Telephone: +45 38621184,  Web: www.drcmr.dkinfo@drcmr.dk |
| Trial site | Research Unit CAMHS, Hellerup | Research Unit, CAMHS, Kildegårdsvej 28, Opgang 3A, 1. sal  2900 Hellerup  Web: www.psykiatri-regionh.dk/Forskningsenheden-boerne-og-ungdomspsykiatri |
| Trial site | CTU | Copenhagen Trial Unit (CTU), Centre for Clinical Intervention Research, Rigshospitalet, Dept. 7812, Blegdamsvej 9, 2100 Copenhagen Ø, Telephone: +45 35457171, Web: [www.ctu.dk](http://www.ctu.dk) |
| * Only the ‘clinical trial sites’ will be recruiting and including participants | | |

## Sponsor and principal investigator

Professor, Anne Katrine Pagsberg

Research unit CAMHS (Principal Investigator at research unit CAMHS)

Kildegårdsvej 28, Opgang 3A, 1. sal

2900 Hellerup

Telephone: +45 38641180

Web: www.psykiatri-regionh.dk/Forskningsenheden-boerne-og-ungdomspsykiatri

Anne.Katrine.Pagsberg@regionh.dk

## Principal investigators

**DRCMR, Hvidovre Hospital**

Professor Hartwig R. Siebner

Kettegard Allé 30

2650 Hvidovre, Denmark

Telephone: +45 36326212

Web: [www.drcmr.dk](http://www.drcmr.dk)

Hartwig.Roman.Siebner@regionh.dk

## Trial Manager

Sofie Heidenheim Christensen

The TECTO trial group, Research Unit CAMHS

Bispebjerg Bakke 30, 16B 1. sal,

2400 Copenhagen NV

Telephone: +45 20543576

Fax: +45 38641001

Web: www.psykiatri-regionh.dk/Forskningsenheden-boerne-og-ungdomspsykiatri

[Sofie.heidenheim.christensen@regionh.dk](mailto:@regionh.dk)

## Steering committee

- Professor Anne Katrine Pagsberg, CAMHS Bispebjerg (Sponsor and Principal Investigator)
- Professor Hartwig R. Siebner, DRCMR, Hvidovre Hospital (Principal Investigator)
- Adjungated professor Frank Verhulst, CAMHS Bispebjerg
- Associate professor Signe Vangkilde, Department of Psychology, University of Copenhagen
- Chief physician, Director of research Janus Christian Jakobsen, CTU
- Head of Department Christian Gluud, CTU
- Head of Clinical Unit B193, Birgitte Bugge, CAMHS Bispebjerg
- Professor Per Hove Thomsen, Child and Adolscent Mental Health Sevices, Aarhus University Hospital, Risskov
- Professor Kerstin Jessica Plessen, Université de Lausanne, Switzerland & CAMHS
- Clinical psychologist Nicole Nadine Lønfeldt, CAMHS Bispebjerg
- Chief clinician for ambulatory services, Nina Staal, CAMHS Bispebjerg

The Steering Committee has the overall responsibility for the planning, conducting and reporting of the trial. Decisions relevant for day-to-day management will be handled by the Executive Committee listed below.

## Executive committee

- Professor Anne Katrine Pagsberg, Research Unit CAMHS
- Professor Hartwig R. Siebner, DRCMR, Hvidovre Hospital
- Head of Department Christian Gluud, CTU

## Advisory board

- Professor Odile van den Heuvel, Vrie University of Amsterdam
- Professor John Piacentini, University of California Los Angeles
- Associate professor Pia Jeppesen, CAMHS Bispebjerg
- Professor Kerstin J Plessen, Université de Lausanne, Switzerland
- Associate Professor Eli Lebowitz, Yale Child Study Center, New Haven, CT, USAProfessor Niklas Rye Jørgensen, Head of the Department of Clinical Biochemistry, Rigshopitalet/Glostrup University HospitalAssociate professor Line Clemmensen, Denmark Technical University, DTU Compute
- Associate professor Stig Bernt Poulsen, University of Copenhagen
- Senior Researcher, PhD, Jens Richardt Møllegaard Jepsen, CAMHS Bispebjerg

## Therapists

- Consultant in child- and adolescent psychiatry Helga K Ingstrup, MD, CAMHS Bispebjerg
- Consultant in child- and adolescent psychiatry Birgitte Borgbjerg Moltke, Mental Health Services, Capital Region
- Clinical psychologist Klara Sofie Vangstrup Halberg, CAMHS Bispebjerg
- Clinical psychologist Anne Kloster, CAMHS Bispebjerg
- Clinical psychologist Pernille Zoega, CAMHS Bispebjerg
- Clinical psychologist Marie Sommer, CAMHS Bispebjerg
- Clinical psychologist Gitte Harboe Sommer, CAMHS Bispebjerg

## Research group

| **Name**  **titles** | **Affiliation** | **Sponsor**  **/PI** | **Steering or Executive Committee** | **Investi-gator** | **Other role** |
| --- | --- | --- | --- | --- | --- |
| Anne Katrine Pagsberg, Professor, MD, PhD | CAMHS Bispebjerg | sponsor | SC + EC |  | Lead overall study managment, training and PhD supervision |
| Hartwig R. Siebner,  Professor, MD, PhD | DRCMR, Hvidovre Hospital | PI | SC + EC |  | Lead overall neuromaging DRCMR, PhD supervision |
| Frank Verhulst Adj. Professor, MD, PhD | CAMHS Bispebjerg |  | SC |  | Academic supervision |
| Signe Vangkilde, Ass. Prof., MSc psych, PhD | Dpt. of Psych., University of Copenhagen |  | SC |  | PhD supervision  Psychological test supervision |
| Birgitte Bugge, MD, head of clinical unit | CAMHS Bispebjerg |  | SC |  | Cooperation with outpatient clinic |
| Per Hove Thomsen, Professor, MD, DMSc | CAMHS, Århus University Hospital |  | SC |  | Expert advice |
| Christian Gluud, Head of Department, MD, DMSc | CTU |  | SC + EC |  | Protocol development, regulatory affairs, data management, statistical analysis, reporting etc. |
| Janus Christian Jakobsen, MD, PhD | CTU |  | SC |  | Protocol development, regulatory affairs, data management, statistical analysis, reporting etc. |
| Jane Lindschou MSc | CTU |  |  |  | Protocol development, regulatory affairs, data management, statistical analysis, reporting etc. |
| Chief clinician for ambulatory services, Nina Staal | CAMHS Bispebjerg |  | SC |  | Coordination with clinic and board of leaders at CAMCH |
| Kerstin J Plessen, Professor, MD, PhD | Université de Lausanne, Switzerland & CAMHS Bispebjerg |  | SC |  | Expert advice  PhD supervision |
| Nicole N. Lønfeldt, MSc psych, PhD, Senior researcher | CAMHS Bispebjerg |  | SC |  | PhD supervision, Therapist |
| Ayna Nejad,  Postdoc, PhD | DRCMR, Hvidovre Hospital |  |  |  | PhD supervision  Daily lead DRCMR |
| William Baaré, Senior Researcher | DRCMR |  |  |  | Data analysis and interpretation of MRI data, supervision, article writing |
| Kasper Winther Andersen, Postdoc | DRCMR |  |  |  | Data analysis and interpretation of MRI data, supervision, article writing |
| Sofie Heidenheim Christensen, MSc Anthropology | CAMHS Bispebjerg |  |  | X | Trial Manager and coordinator |
| Valdemar Funch Uhre  MSc Psych, PhD student | CAMHS Bispebjerg & DRCMR |  |  | X | PhD study: Neural and Neurocognitive mediators of effectiveness |
| Camilla Funch Uhre,  MSc Psych, PhD student | CAMHS Bispebjerg |  |  | X | PhD study: Neurocognitive mediators of effectiveness |
| Linea Pretzmann,  MSc Psych, PhD student | CAMHS Bispebjerg |  |  | X | PhD study:  Adverse events in CBT for OCD |
| Cecilie Mora- Jensen  MD, PhD student | CAMHS Bispebjerg |  |  | X | PhD study: SOOTHe – Salivary Oxytocin in OCD Treatment - Helping families |
| Christine Thoustrup,  MSc psych, PhD student | CAMHS Bispebjerg |  |  | X | PhD study:  Emotion regulation abilities in peaditric OCD |
| Nicoline L. J. Korsbjerg,  MSc Psych., PhD student applicant | CAMHS Bispebjerg |  |  | X | Planned PhD study: Precision psychotherapy for OCD – a TECTO sub-study |
| Melanie Ritter  MsC Psych., PhD student applicant | CAMHS,  Bispebjerg |  |  | x | Planned PhD study:  Neurocognitive correlates of psychotherapeutic treatment response in pediatric Obsessive-Compulsive Disorder (OCD) |
| Julie Hagstrøm, MSc Psych, PhD, Postdoc | CAMHS Bispebjerg |  |  | X | Research clinician, therapist |
| Klara Sofie Vangstrup Halberg  MSc Psych. | CAMHS Bispebjerg |  |  | x | Research clinician, therapist |
| Iben Clemmesen, MSc Psych. | CAMHS Bispebjerg |  |  | X | Research clinician |
| Amanda Aa. Gudmandsen  MSc Psych. | CAMHS Bispebjerg |  |  | X | Research clinician |
| Emilie D. Thorsen  Psychology student | CAMHS  Bispebjerg |  |  | X | Research assistent |
| Frederik G. Espensen  Psychology student | CAMHS, Bispebjerg |  |  | X | Research assistent |
| Anton H. Hougaard  Psychology student | CAMHS, Bispebjerg |  |  |  | Research assistent |
| Helga K. Ingstrup, MD, consultant | CAMHS Bispebjerg |  |  |  | Therapist |
| Psychologist Gitte Sommer | CAMHS Bispebjerg |  |  |  | Therapist |
| Katja Hybel  MSc psych, PhD | Aarhus University Hospital |  |  |  | Supervisor (ad hoc) and instructor FCBT |
| Katrin F Larsen  BA head nurse | CAMHS Bispebjerg |  |  |  | Supervisor (regular) and instructor FPRT |
| Anne Lydolff  MSc psych | CAMHS Bispebjerg |  |  |  | Supervisor (regular) FCBT |
| Professor Niklas Rye Jørgensen | Dpt Clinical Biochemistry, Rigshopitalet/Glostrup University Hospital |  |  |  | Counsellor and collaborator on oxytocin analyses |
| Associate professor Line Clemmensen | Denmark Technical University, DTU Compute |  |  |  | Counsellor and collaborator on machine learning and data integration |
