# Supplementary material for: Family-based cognitive behavioural therapy versus family-based relaxation therapy for obsessive-compulsive disorder in children and adolescents: protocol for a randomised clinical trial (the TECTO trial)
Source: BMC Psychiatry. 2022 Mar 19;22:204. doi: 10.1186/s12888-021-03669-2 (PMC8933964; doi:10.1186/s12888-021-03669-2)
Supplement: Supplementary file 3 — Additional file 3. [file 12888_2021_3669_MOESM3_ESM.docx]

**
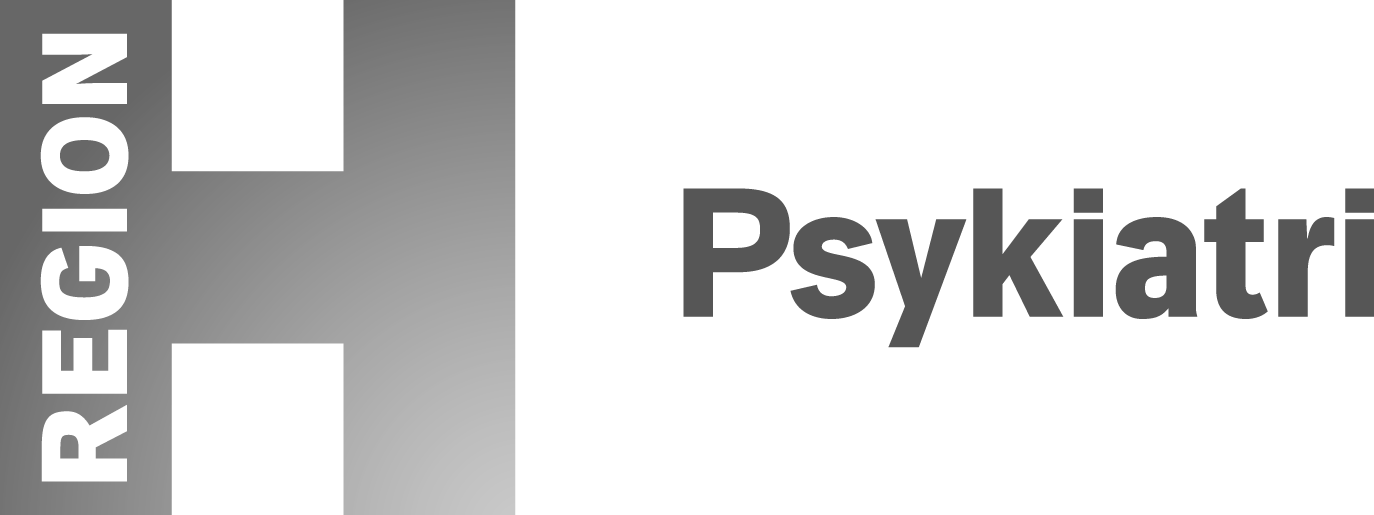
**
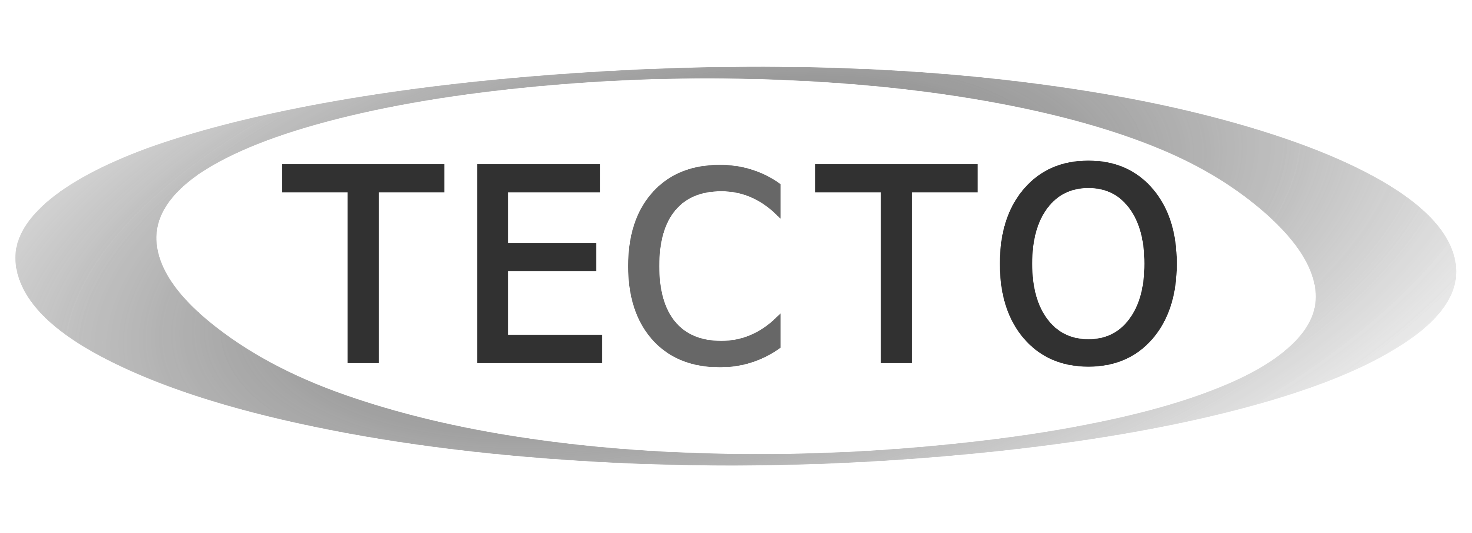


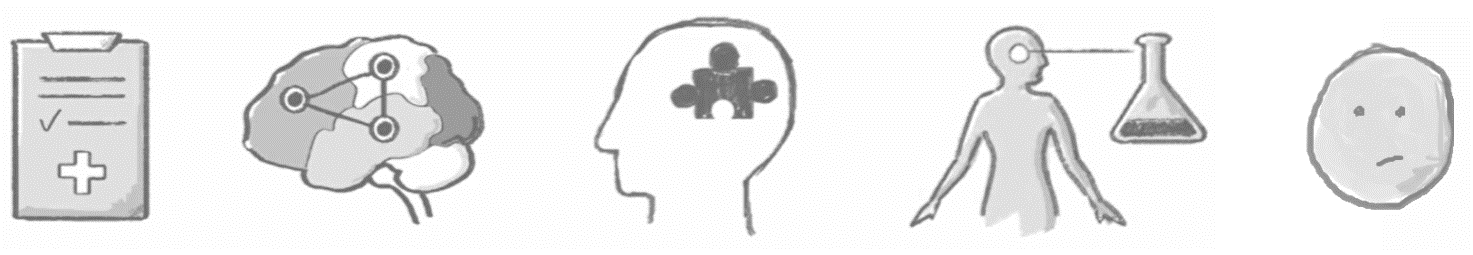

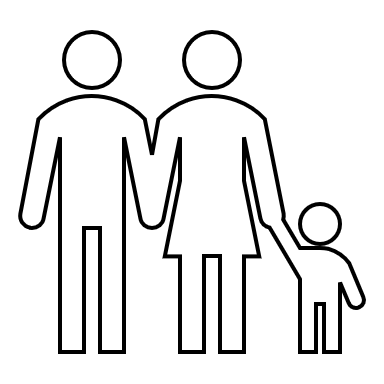


Information on participation in a research project about OCD

*To parents*

## Information on participation in a research project about OCD

Project title: TECTO Trial

**TO PARENTS**

Your child has been referred for symptoms of OCD (Obsessive-Compulsive Disorder) and has been assessed at the Child and Adolescent Psychiatric Centre, Bispebjerg. Based on the results of the examination, your child has been diagnosed with OCD. We would therefore like to ask you if your son/daughter would like to participate in a scientific study of family-based therapy for children and adolescents with OCD. The research project is being carried out by the Child and Adolescent Psychiatric Centre, Capital Region, in collaboration with the MR Department at Hvidovre Hospital.

Before you decide whether your son/daughter should take part in the study, you need to fully understand what the study is about and how we conduct it. We therefore ask you to read this participant information carefully.

The aim of the project is to gain knowledge that can improve treatment for children and adolescents with OCD.

If you decide that your son/daughter should take part in the trial, we will ask you to sign a consent form. Remember that you have the right to consider your decision before deciding whether to sign the consent form.

Participation in the trial is voluntary. You can withdraw consent at any time without giving a reason. It will not affect your child's further treatment if you refuse to participate in the study or if you withdraw your child from the study during the process.

#### Purpose of the study

In our project, we will investigate the effects of family-based therapy and the brain areas and brain processes affected by the therapy in children and adolescents. We will do this by comparing the therapy form with another family-based therapy form. We will also look at OCD symptoms, family life, your child's quality of life, and how your child functions in everyday life.

#### Participation in the project

If you decide that your child should take part in the study, your child will receive one of two types of therapy, both of which consist of therapeutic sessions with one of our therapists. The therapy includes 14 sessions spread over 16 weeks. The therapists are all trained in the two types of therapy and are either psychologists or psychiatric specialists. Both therapies include training in OCD and home exercises. In addition, the therapist will give you ongoing guidance on how best to support your child. The two therapies are similar in most respects, but differ in the psychological approach that will be reflected in the exercises. It is this difference that we want to explore the significance of. Research suggests that both types of therapy are effective in treating OCD. We have chosen not to name the treatments, as this will make it easier to keep the treatment secret from our researchers, so that they can obtain as neutral a measurement as possible. The study will end after 16 weeks, regardless of how many sessions your child has attended. There will be a number of research studies during the course of the programme. In the section "Research studies and programme" on pages 3-5, you can read more about each study and when it is. Six months after the end of the therapy programme, we will ask you to come back for a follow-up study.

#### The study

The study is a randomized trial. This means that the type of treatment we offer your child will be randomly assigned. The random allocation helps to ensure the quality of the project, and you therefore cannot choose which treatment your child will receive in the project. If we assess that further treatment is needed after the programme, your child will be offered to continue with regular treatment.

We plan to include 128 children and adolescents aged 8 to 17 who have OCD and 256 parents of children and adolescents with OCD. It will also include 128 children and adolescents of the same age who do not have OCD and 256 parents of children and adolescents without OCD. We are doing this because we want to compare children and young people who do not have OCD with children and young people who do have OCD. Similarly, we will compare parents of children and adolescents who have OCD with parents of children and adolescents without OCD.

#### Other treatment options

If you do not wish to participate in the project, this will not affect your child's right to current or future treatment. If you choose to participate, you can withdraw from the study at any time. If you withdraw from the project, your child will still be entitled to receive the treatment he/she would have received if he/she had not participated in the project. Your child will therefore not lose any rights as a patient by taking part in the project.

In the table below, we have outlined the main differences between receiving treatment in our research project and receiving the usual treatment in the clinic. It is important to stress that the content of the usual treatment for OCD may vary. Therefore, we cannot guarantee that your treatment will be within the described range if your child starts usual treatment in the clinic.

|  | **OCD research project** | **Common treatment** |
| --- | --- | --- |
| **Number of sessions** | 14 sessions | Varies according to severity and effect of treatment |
| **Duration of session** | 75 min. | Typically 45-60 min. |
| **Time of session** | Typically fixed weekly sessions spread over 16 weeks.  Open Mon-Fri: 8-16 | Typical fixed weekly sessions  Open Mon-Fri: 8-16 |
| **Format** | Individual therapy  Both parents participate in 5 full sessions  At least one parent attends the last 30 minutes. in the remaining sessions | Individual or group therapy  Parents are involved to varying degrees depending on the age of the child/young person |
| **Research activity** | Total time spent by participant approx. 29 hours. For details see page 4 | No |
| **Location** | Primarily B193/B195, Child and Adolescent Psychiatric Centre. Bispebjerg  And 2 days at MR scanning department, Hvidovre Hospital | Child and Adolescent Psychiatric Centre in your associated wards |
| **Remuneration** | Transport: compensation for research activities  Gift card: given per long day of study (see page 8 - Compensation) | No |

#### Absence or interruption of the trial

Participation in the survey is voluntary and if you take part, you can withdraw your consent *at any time*. If you do withdraw your consent, it will be beneficial to the research if you tell us the reason, as this may otherwise affect our results, but if you do not wish to tell us why, please do not do so.

It is also optional whether you want to receive information about your son/daughter's examination results, except for MRI scans. If you consent to us taking an MRI scan of your child's brain, we are obliged to inform you of the results if we detect any abnormal changes in your child's brain (see section on risks and disadvantages). It is rare that this will be the case. If you do not wish to receive information about abnormal changes in your child's brain, you can opt out of having your child MRI scanned. Your child can still be included in the study, even if you do not want your child to be scanned.

Your child will be closely monitored during the study and treatment, and if we decide that the project's treatment is unsuitable for your child, or if your child is expected to get much worse, we will withdraw him/her from the project to find a treatment better suited to your child. If your child fails to attend three consecutive scheduled sessions without cancelling, we will bring forward the final examinations and then end the study. Your child will then continue in regular treatment if you wish and there is a need.

#### Research studies and programme

In addition to the treatment, your child will take part in various studies with the researchers, where we measure the effect of the therapy. The chart on page 5 gives you an overview of when you will have to take part in studies and for how long. In the following we will go through the types of studies you and your child will take part in.

**Parental participation**

Both therapies are family-based, so it is important that parents are present at each session. In five of the sessions, parents must be present for the entire session and in the remaining sessions, parents must be present for the last 30 minutes of the session. It is preferable that both parents attend, but as we realise that it can be difficult to fit this into everyday life, it is also perfectly fine if it is just one parent. If possible, please plan for the same parent to attend the sessions (with the exception of MR test days).

As parents, you will be asked to fill in questionnaires, participate in heart rhythm puzzles with your child and hand in saliva samples, which you can read more about below. It is voluntary for you to fill in questionnaires, participate in the puzzles, have your heart rate measured and provide saliva samples, and if you do, you can withdraw your consent *at any time*. You have also received separate consent forms and separate parental participation information.

**Tasks and questionnaires**

Before your child starts treatment, and after your child is discharged from treatment, he/she will have to complete different types of tasks designed to test problem-solving strategies, attention function and memory. We will also ask you and your child to answer various questionnaires about symptoms of OCD, family life, how your child functions in everyday life and his/her quality of life.

**Magnetic resonance imaging (MRI) of the brain**

During an MRI scan, we take pictures of your child's brain while he/she performs computer tasks.

An MRI scanner consists of a large tubular magnet. Your child lies on a tray, which is led into the tube. Before the scan, we will inform your child about what he/she will do inside the scanner and the whole process will be practised in a so-called MOCK scanner (scanner simulator) so that he/she is prepared for what will happen. The person doing the scan will sit in a room next to you, and throughout the examination your child will be able to get in touch with the staff by pressing a rubber ball. On the test day before, your child will try a virtual reality version of being MRI scanned.

We perform the MR scans at the MR department (section 340B) at Hvidovre Hospital. We can arrange for a taxi to take you there.

**Interviews**

During the course of treatment, your child will be interviewed by health professionals about the progress of his/her OCD symptoms and any adverse symptoms of treatment.

**Puzzles**

You will be asked to have one of you assist your child in completing different difficult puzzles two times during the process. We will also measure yours and your child's heart rate before, during, and after the puzzle.

**Saliva tests**

You and your child will be asked to give saliva samples (max 2.8 mililitres each), four times during the process. We will use the saliva samples to measure levels of the hormone oxytocin, which is thought to be important for attachment between people. When saliva sampling, you will be asked to chew on cotton for 60 seconds. It is important that you do not eat 2 hours before or drink anything half an hour before the samples are taken.

**Obtaining investigation information**

To avoid your child having to undergo the same tests several times, we will obtain information from examinations, tests and video recordings collected during your child's assessment in the department. If it has been less than a year since your child was seen in the psychiatric department, it may be necessary to obtain data from individual tests carried out in previous sessions. This is because individual tests are not meaningful if they are repeated within a year.

**Video recording**

We film all sessions with the therapist, as well as tests and interviews, so that researchers and clinical staff can ensure the quality of treatment and research data.

#### Study programme

In the figure below, you can see when the different examinations take place and how long they last.

Tasks, questionnaires, interviews, puzzles and saliva tests (5 hours)

)

MR scan (3 hours)

**DAY 1**

**DAY 2**

**Starting treatment**

Questionnaires (10 min.)

Interviews and questionnaires (2 hours)

)

))

Interviews, questionnaires and saliva tests (2 hours)

Questionnaires (15 min.)

Interviews (3½ hours)

**Treatment completed**

Tasks, questionnaires, interviews, quizzes and saliva tests (5 hours)

MR scan (3 hours)

Interviews, questionnaires, puzzles and saliva tests (2 hours)

**6 months**

**Studies in progress week**

**1**

**4**

**8**

**15**

**16**

**DAY 1**

**DAY 2**

**STUDY PROGRAM**

#### Risks and disadvantages

Please note that if you choose to participate in the trial, you and your child may experience absence from work and school for research activities and therapy sessions. We have described the risks associated with the different parts of the research below.

**Therapy, interviews and research studies**

There are no known risks associated with the two types of therapy or the studies in which the child participates.

**MR scan**

There are no known risks associated with MR scanning. The scan is painless and cannot be felt in the body. The scanner uses radio waves and a magnetic field to take the images.

As the MRI scanner contains a strong magnetic field, it is important that you tell us if your child:

- Has metal objects implanted in the body (e.g. pacemaker or insulin pump)
- Has metal splinters in the eye
- Has metal bracket for teeth straightening
- Is pregnant

Individuals may experience stress and claustrophobic discomfort. If your child experiences claustrophobia or discomfort during the scan, we will take your child out of the scanner immediately.

In rare cases, we may find abnormal changes in the scan images of your child's brain that are not already known in the child. Only very rarely will these be changes that have a health significance, but if they are, it may affect future opportunities to take out private health or life insurance. You should therefore be aware that if you *do not* want information about any abnormal changes found in your child's brain, and you *do not* want us to pass this information on to your own doctor and/or the relevant hospital department, you must decline to have the child MRI scanned.

We would like to emphasise that the MRI scan is for research purposes and is not a diagnostic scan. You cannot therefore regard the scan as a health check.

**New knowledge on risks and drawbacks**

We do not expect any risks for your child by participating in the study. You should be aware that there can always be unpredictable risks and stresses associated with participation in health science research. Although there are no known risks associated with the study, unexpected risks can never be completely ruled out. Please let us know if you experience any health problems with your child while the study is ongoing. Of course, if we discover new possible side effects during the course of the study that we have not already told you about, you will be informed immediately and you will have to decide whether you want your child to continue in the study. If, contrary to expectations, your child suffers injuries as a result of taking part in the research project, you can seek compensation under the Patient Insurance Act. The project has been approved by the Scientific Ethics Committees of the Capital Region (protocol no. H-18010607).

#### Anonymity, data retention and data processing

All information about your child that is obtained through the research project is covered by the rules on confidentiality. By giving consent, you allow your child's results to be included in the research in an anonymised form. Any material that can be identified (e.g. name or video recording) will be stored anonymously with a code. This code is only available to the research unit at Child and Adolescent Mental Health Centre and the Copenhagen Trial Unit, Rigshopitalet, Region Hovedstaden, which is responsible for data storage. Thus, only the project staff can recognise the individual participant. You have the possibility to obtain access to the file according to the rules of the Public Access Act.

We will keep the code to identify the anonymised data until the time when the data processing ends. This includes data on sex and age, which will be used for the statistical analyses. As we would like to be able to contact you during the study and contact you about the study results (if you wish) or anything else afterwards, we will keep your name, phone number and address. We will also have video recordings of the investigation, examinations and treatment available until the end of the data processing.

Data will be stored by Copenhagen Trial Unit, Rigshopitalet, Region Hovedstaden. We store video recordings and data from the various tests confidentially in protected folders on the Capital Region's server. MR images are stored confidentially in protected folders on the DRCMR (Danish Research Center for Magnetic Resonance) server. We collect saliva samples at Bispebjerg Hospital and transport them to a research biobank at the Clinical Biochemistry Department, Rigshospitalet Glostrup, where they are stored in a locked freezer in a locked room. We follow the rules of the Danish Data Protection Authority and the storage is notified and approved by the Danish Data Protection Authority.

Our plan is to include participants from summer 2018 and three years onwards. The last participant will finish in the summer of 2022, after which we will analyse the results. We expect to have completed the analyses and to be working on publishing scientific papers during 2022. In case of any delays, we will complete the data processing by 01/04 2027. All personally identifiable samples and data, except saliva samples, will be destroyed at the end of the study. Saliva samples will be kept for a maximum of six months after the last participating family's samples have been collected. They will then be destroyed. The samples can only be used in a new research project if a scientific ethics committee gives permission.

During the course of your child's treatment, information may be passed on to the departmental therapist in charge of the case, when your therapist or research team considers it important for your child's treatment. For example, this may be for the purpose of further assessment or treatment. If you do not want this, you can opt out in the consent form.

#### Compensation

To compensate for the time you spend on the project, we offer an allowance to you and your child in the form of 5 gift vouchers of 250 DKK each (1250 DKK in total) to www.sendentanke.dk. We will give a gift voucher for each of the long test days before and after the treatment. This amount is taxable (remember to report this on your annual statement to the tax authorities). If your child has a free card, it will not matter unless she/he earns more than the free card amount in total in a year.

#### Initiative and economic conditions

The initiator of the project is the former head of the research unit at the Child and Adolescent Psychiatric Centre of the Capital Region, Professor Kerstin Plessen, employed at the University of Copenhagen. Professor and senior physician Anne Katrine Pagsberg, employed at the same institution, is leading the project.

The project is funded by the Research Pool of the Capital Region of Denmark Psychiatry (1,659,000), the Capital Region of Denmark Research Fund (DKK 1,475,000), the Lundbeck Foundation (DKK 1,575,000, ref.: R191-2015-922 and DKK 390,000, ref.: R211-2015-3990), the Gangsted Foundation (DKK 216,000, ref.R433-A29811), Holm's Memorial Grant (DKK 86,533, ref.: 20006-1951), Sofus Carl Emil Friis and Olga Friis' Wife Grant (DKK 507,736), Psychiatric Research Fund of 1967 (DKK 50,000), Network for Research and Quality Assurance in Psychotherapy and Child and Adolescent Psychiatric Centre (DKK 10,000). We continue to seek additional funding for the project. The investigators have no financial ties to funders.

#### Complaints

If your child is subjected to unsatisfactory treatment, you have the possibility to complain and we will help you with further information and relevant forms.

#### Access to trial results

We expect the research project to be completed by the end of 2022. The results of the research project will be published in international scientific journals and in the media and may thus help to provide a basis for improving the treatment of OCD internationally.

If you would like to receive information about the results of the research project when it is completed, you can tick a box on the consent form.

We hope that this information has given you enough insight into what it means to participate in the trial and that you feel equipped to make a decision about your child's possible participation. We also ask you to read the attached material "The rights of the particiapant in a research project".

If you want to know more about the project, please contact:

Project coordinator Sofie Heidenheim Christensen, Research Unit BUC, Department Bispebjerg, Bispebjerg Bakke 30, 2400 Kbh NV

Tel: 20543576

Mail: [rhp-tecto@regionh.dk](mailto:rhp-tecto@regionh.dk)

Yours sincerely,

Anne Katrine Pagsberg

Professor, Senior Physician, PhD

Region Hovedstadens Psykiatri, Børne- og Ungdomspsykiatrisk Center and University of Copenhagen

Mail: Anne.Katrine.Pagsberg@regionh.dk


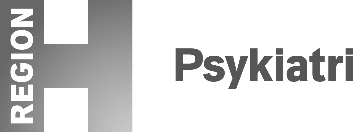

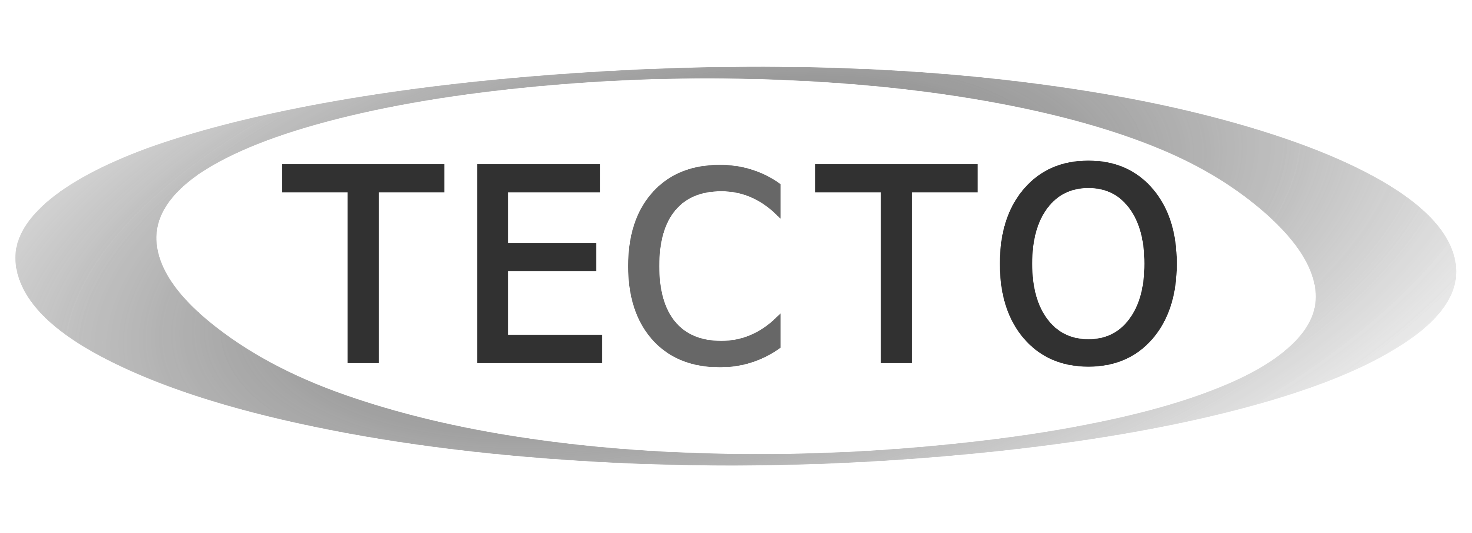


Information on participation in a research project about OCD

*18 years*


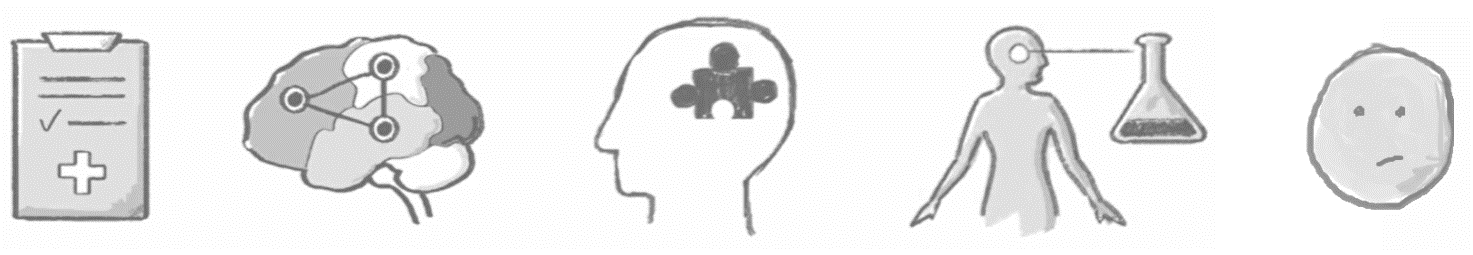

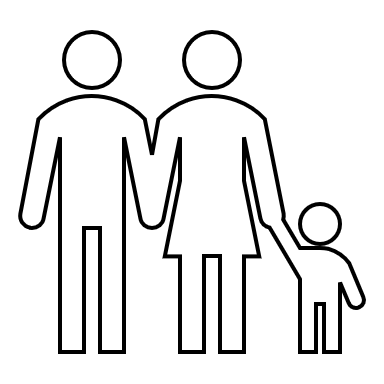


## Information on participation in OCD research project

Project title: TECTO Trial

**FOR YOUNG PEOPLE OF LEGAL AGE - 18 YEARS**

You have been referred because of symptoms of OCD (Obsessive-Compulsive Disorder) and have been assessed at the Child and Adolescent Psychiatric Centre, Bispebjerg. Based on the results of the examination, you have been diagnosed with OCD. We would therefore like to ask you if you would like to participate in a scientific study of family-based therapy for children and adolescents with OCD. The research project is being conducted by the Child and Adolescent Psychiatric Center, Capital Region, in collaboration with the MR Scanning Department at Hvidovre Hospital.

Before you decide whether to take part in the study, you need to fully understand what the study is about and how we conduct it. We therefore ask you to read this Participant Information carefully.

The aim of the project is to gain knowledge that can improve treatment for children and adolescents with OCD.

If you decide that you would like to take part in the trial, we will ask you to sign a consent form. Remember that you have the right to a period of reflection before deciding whether to sign the consent form.

Participation in the trial is voluntary. You can withdraw consent at any time without giving a reason. It will not affect your further treatment if you refuse the study or if you withdraw from the study during the process.

#### Purpose of the study

In our project, we will investigate the effects of a family-based talk therapy and which brain areas and brain processes the therapy affects in children and adolescents. We will do this by comparing the form of therapy with another form of family-based therapy. We will also look at OCD symptoms, family life, your quality of life and how you function in everyday life.

#### Participation in the project

If you agree to take part in the study, you will receive one of two types of therapy, both of which consist of therapeutic sessions with one of our therapists. The therapy consists of 14 sessions spread over 16 weeks. The therapists are all trained in the two types of therapy and are either psychologists or psychiatric specialists. Both therapies include training in OCD and home exercises. In addition, the therapist will give your parents ongoing guidance on how best to support you. The two therapies are similar in most respects, but differ in the psychological approach that will be reflected in the exercises. It is this difference that we want to explore. Research suggests that both therapies are effective in treating OCD. We have chosen not to name the treatments, as this will make it easier to keep the treatments secret from our researchers, so that they can record as neutral a measurement as possible. The study will end after the 16 weeks, regardless of how many sessions you have attended. There will be a number of research studies during the course of your treatment. In the section "Research studies and programme" on pages 3-5, you can read more about each study and when it will take place. Six months after the end of the therapy programme, we will ask you and your parents to come back for a follow-up study.

#### The study

The study is a randomized trial. This means that the type of treatment we offer you will be randomly assigned. The random allocation helps to ensure the quality of the project, and you therefore cannot choose which treatment you will receive in the project. If we assess that further treatment is needed after the trial, you will be offered to continue with regular treatment.

It is planned to include 128 children and adolescents aged 8 to 17 who have OCD and 256 parents of children and adolescents with OCD. It will also include 128 children and adolescents of the same age who do not have OCD and 256 parents of children and adolescents without OCD because we want to compare them and their parents with children and adolescents with OCD and their parents. We do this because we want to compare children and young people who do not have OCD with children and young people who do have OCD. Similarly, we want to compare parents of children and young people who have OCD with parents of children and young people without OCD.

#### Other treatment options

If you do not wish to participate in the project, this will not affect your entitlement to current or future treatment. If you choose to participate, you can withdraw from the project at any time. If you withdraw from the project, you will still be entitled to receive the treatment you would have received if you had not participated in the project. So you do not lose any rights as a patient by participating in the project.

In the table below, we have outlined the main differences between receiving treatment in our research project and receiving the usual treatment in the clinic. It is important to stress that the content of the usual treatment for OCD may vary. We cannot therefore guarantee that your course of treatment will be within the outlined framework if you start usual care in the clinic.

|  | **OCD research project** | **Common treatment** |
| --- | --- | --- |
| **Number of sessions** | 14 sessions | Varies according to severity and effect of treatment |
| **Duration of session** | 75 min. | Typically 45-60 min. |
| **Time of session** | Typically fixed weekly sessions spread over 16 weeks.  Open Mon-Fri: 8-16 | Typically fixed weekly sessions  Open Mon-Fri: 8-16 |
| **Parental participationFormat** | Individual therapy  Both parents participate in 5 full sessions  At least one parent attends the last 30 minutes. in the remaining sessions | Individual or group therapy  Parents are involved to varying degrees depending on the age of the child/young person |
| **Research activity** | Total time spent by trial participant is approximately 29 hours. For details see page 4 | No |
| **Location** | Primarily B193/B195, Child and Adolescent Psychiatric Centre. Bispebjerg  And 2 days at MR scanning department, Hvidovre Hospital | Child and Adolescent Psychiatric Centre in your associated ward |
| **Remuneration** | Transport: compensation for research activities  Gift card: given per long day of study (see page 8 - Compensation) | No |

#### Absence or interruption of the trial

Participation in the study is voluntary and if you take part, you can withdraw your consent *at any time*. If you withdraw your consent, it will benefit the research if you tell us the reason as it may affect our outcome measures, but if you do not want to tell us why, please do not do so.

It is also voluntary whether you want to receive information about your examination results, except for MRI scans. If you consent to us taking an MRI scan of your brain, we are obliged to inform you of the results if we detect abnormal changes in your brain (see section on risks and drawbacks). However, this will rarely be the case. If you do not wish to receive information about abnormal changes in your brain, you can opt out of having an MRI scan. You can still be included in the study even if you do not want to have an MR scan.

You will be closely monitored during the study and treatment, and if we decide that the project's treatment is unsuitable for you, or you are likely to get much worse, we will withdraw you from the project to find a treatment more suitable for you. If you fail to attend three consecutive scheduled sessions without cancelling, we will bring forward the final tests and then end the study. You will then continue in regular treatment if you and your family wish and there is a need.

#### Research studies and programme

In addition to the treatment, you will take part in various studies with the researchers, where we measure the effect of the therapy. The figure on page 6 gives you an overview of when you will have to take part in studies and for how long. In the following we will go through the types of studies you will be taking part in.

**Parental participation**

Both therapies are family-based, so it is important that parents are present at each session. In five of the sessions, parents must be present for the entire session and in the remaining sessions, parents must be present for the last 30 minutes of the session. It is preferable that both parents attend, but as we realise that it can be difficult to fit this into everyday life, it is also perfectly fine if it is just one parent. If possible, please allow your parents to schedule the same parent to attend the sessions (with the exception of MR testing days).

**Tasks and questionnaires**

Before you start treatment and after you are discharged from treatment, you will have to complete different types of tasks designed to test problem-solving strategies, attentional function and memory. We will also ask you and your parents to answer various questionnaires about symptoms of OCD, family life, your quality of life and how you function in everyday life.

**Magnetic resonance imaging (MRI) of the brain**

During an MRI scan, we take pictures of your brain while you perform computer tasks.

An MRI scanner consists of a large tubular magnet. You lie on a tray, which is driven into the tube. Before the scan, we will inform you about what you will do inside the scanner and the whole process will be practised a few times in a so-called MOCK scanner (scanner simulator) so that you are prepared for what will happen. The person doing the scan will sit in a room next to you, and throughout the examination you will be able to get in touch with the staff by pressing a rubber ball. On the test day before, you will try a virtual reality version of being MRI scanned.

We perform the MR scans at the MR department (section 340B) at Hvidovre hospital. We can arrange a taxi for you.

**Interviews**

During the course of treatment, you will be interviewed by healthcare professionals about the progress of your OCD symptoms and any unwanted symptoms of treatment.

**Puzzles**

Four times during the programme, you and one of your parents will be asked to complete different difficult puzzles. We will also measure your and your parent's heart rate before, during and after the puzzle.

**Saliva tests**

You and your parents will be asked to take saliva samples (max 2.8 mililitres each), four times during the course. We will use the saliva samples to measure levels of the hormone oxytocin, which is thought to be important for attachment between people. When saliva sampling, you will be asked to chew on cotton wool for 60 seconds. It is important that you do not eat 2 hours before or drink anything half an hour before the samples are taken.

**Obtaining investigation information**

To avoid you having to do the same tests several times, we will collect information from examinations, tests and video recordings collected during your assessment in the department. If it has been less than a year since you have been in psychiatric care, it will sometimes be necessary to collect data from individual tests carried out in previous care.is because individual tests are not meaningful if they are repeated within a year.

**Video recording**

We film all sessions with the therapist, as well as tests and interviews, so that researchers and clinical staff can ensure the quality of treatment and research data.

#### Study programme

In the figure below, you can see when the different examinations take place and how long they last.

**6 months**

**DAY 2**

**DAY 1**

**DAY 2**

Interviews, questionnaires and saliva tests (2 hours)

Questionnaires (15 min.)

Tasks, questionnaires, interviews, puzzles and saliva tests (5 hours)

)

MR scan (3 hours)

**DAY 1**

**Starting treatment**

Questionnaires (10 min.)

Interviews and questionnaires (2 hours)

)

))

Interviews (3½ hours)

**Treatment completed**

Tasks, questionnaires, interviews, quizzes and saliva tests (5 hours)

MR scan (3 hours)

Interviews, questionnaires, puzzles and saliva tests (2 hours)

**Studies in progress week**

**1**

**4**

**8**

**15**

**16**

**SURVEY PROGRAM**

#### Risks and disadvantages

Please note that if you choose to participate in the trial, you may experience absences from school or work for research activities and therapy sessions. We have described the risks associated with the different parts of the research below.

**Therapy, interviews and research studies**

There are no known risks associated with the two types of therapy or the studies in which you participate.

**MR scan**

There are no known risks associated with MR scanning. The scan is painless and cannot be felt in the body. The scanner uses radio waves and a magnetic field to take the images.

As the MRI scanner contains a strong magnetic field, it is important that you tell us if you:

- have metal objects implanted in the body (e.g. pacemaker or insulin pump)
- has metal splinters in the eye
- has metal bracket for teeth straightening on the teeth
- is pregnant

Individuals may experience stress and claustrophobic discomfort. If you experience claustrophobia or other discomfort during the scan, we will take you out of the scanner immediately.

In rare cases, we may detect abnormal changes on the scan images of your brain that are not known to you beforehand. Only very rarely will these be changes that have a health significance, but if they are, it may affect your future ability to take out private health or life insurance. You should therefore be aware that if you *do not* want information about any abnormal changes found in your brain, and you *do not* want us to pass this information on to your own doctor and/or the relevant hospital department, you should decline to have an MRI scan.

We would like to stress that the MRI scan is for research purposes and is not a diagnostic scan. Therefore, you cannot consider the scan as a health check.

**New knowledge on risks and drawbacks**

We do not expect any risks for you by participating in the study. However, you should be aware that there can always be unpredictable risks and stresses associated with participation in health science research. Thus, although there are no known risks associated with the study, unexpected risks can never be completely ruled out. Please let us know if you experience any health problems during the trial. Of course, if we discover new possible side effects during the course of the study that we have not already told you about, you will be informed immediately and you will have to decide whether you want to continue in the study. If, contrary to expectations, you suffer injuries as a result of taking part in the research project, you can seek compensation under the Patient Insurance Act. The project has been approved by the Scientific Ethics Committees of the Capital Region (protocol no. H-18010607).

#### Anonymity, data retention and data processing

All information about you obtained through the research project is covered by the rules on confidentiality. By consenting, you allow your results to be included in the research in an anonymised form. Any material that can be identified (e.g. name or video recording) will be stored anonymously with a code. This code is only available to the research unit at BUC and the Copenhagen Trial Unit, Rigshopitalet, Region Hovedstaden, which is responsible for data storage. Thus, only the project staff can recognise the individual participant. You have the possibility to obtain access to the file according to the rules of the Public Access Act.

We will keep the code to identify the anonymised data until the time if the data processing has ended. This includes data on gender and age, which will be used for the statistical analysis. As we would like to be able to contact you during the study and to contact you about the study results or anything else afterwards, we will keep your name, telephone number and address. We will also have video recordings of the investigation, examinations and treatment available until the end of the data processing.

Data will be stored by Copenhagen Trial Unit, Rigshopitalet, Region Hovedstaden. We store video recordings and neuropsychological test data confidentially in protected folders on the Capital Region server. MR images are stored confidentially in protected folders on the DRCMR (Danish Research Center for Magnetic Resonance) server. We collect saliva samples at Bispebjerg Hospital and transport them to a research biobank at the Clinical Biochemistry Department, Rigshospitalet Glostrup, where they are stored in a locked freezer in a locked room. Data Protection Authority rules are followed and the storage is notified and approved by the Data Protection Authority.

Our plan is to include participants from summer 2018 and three years onwards. The last participant will finish in the summer of 2022, after which we will analyse the results. We expect to have completed analyses and work on publishing scientific papers during 2022. In case of any delays, we will complete data processing by 01/04 2027. All personally identifiable samples and data, except saliva samples, will be destroyed at the end of the study. Saliva samples will be kept for a maximum of six months after the last participating family's samples have been collected. They will then be destroyed. The samples can only be used in a new research project if a scientific ethics committee gives permission.

During the course of your treatment, information may be passed on to the therapist in the department responsible for the case, when your therapist or the research team considers it important for your treatment. For example, this may be for the purpose of further assessment or treatment. If you do not want this, you can opt out in the consent form.

#### Compensation

To compensate you for the time you spend on the project, we offer you and your parents an allowance in the form of 5 gift vouchers of 250 DKK each (1250 DKK in total) to www.sendentanke.dk. We will give one gift voucher for each of the long test days before and after the treatment. This amount is taxable (remember to report this on your annual statement to the tax authorities). If you have a free card, it will not matter unless you earn more in total than the free card amount in a year.

#### Initiative and economic conditions

The initiator of the project is the former head of the research unit at the Child and Adolescent Psychiatric Centre of the Capital Region, Professor Kerstin Plessen, employed at the University of Copenhagen. Professor and senior physician Anne Katrine Pagsberg, employed at the same institution, is leading the project.

The project is funded by the Research Pool of the Capital Region of Denmark Psychiatry (1,659,000), the Capital Region of Denmark Research Fund (DKK 1,475,000), the Lundbeck Foundation (DKK 1,575,000, ref.: R191-2015-922 and DKK 390,000, ref.: R211-2015-3990), the Gangsted Foundation (DKK 216,000, ref.R433-A29811), Holm's Memorial Grant (DKK 86,533, ref.: 20006-1951), Sofus Carl Emil Friis and Olga Friis' Wife Grant (DKK 507,736), Psychiatric Research Fund of 1967 (DKK 50,000), Network for Research and Quality Assurance in Psychotherapy and Child and Adolescent Psychiatric Centre (DKK 10,000). We continue to seek additional funding for the project. The investigators have no financial ties to funders.

#### Complaints

If you are subjected to unsatisfactory treatment, you have the possibility to complain, and we will assist you with further information and relevant forms.

#### Access to trial results

We expect the research project to be completed by the end of 2022. The results of the research project will be published in international scientific journals and in the media and may thus help to provide a basis for improving the treatment of OCD internationally.

If you would like to receive information about the results of the research project once it is completed, you can tick a box on the consent form.

We hope that this information has given you enough insight into what it means to participate in the trial and that you feel equipped to make a decision about your possible participation. We also ask you to read the attached material "The rights of the subject in a research project".

If you want to know more about the project, please contact:

Project coordinator Sofie Heidenheim Christensen, Research Unit BUC, Department Bispebjerg, Bispebjerg Bakke 30, 2400 Copenhagen

Tel: 20543576

Mail: [rhp-tecto@regionh.dk](mailto:rhp-tecto@regionh.dk)

Yours sincerely,

Anne Katrine Pagsberg

Professor, Senior Physician, PhD

Region Hovedstadens Psykiatri, Børne- og Ungdomspsykiatrisk Center and University of Copenhagen

Mail: Anne.Katrine.Pagsberg@regionh.dk

**
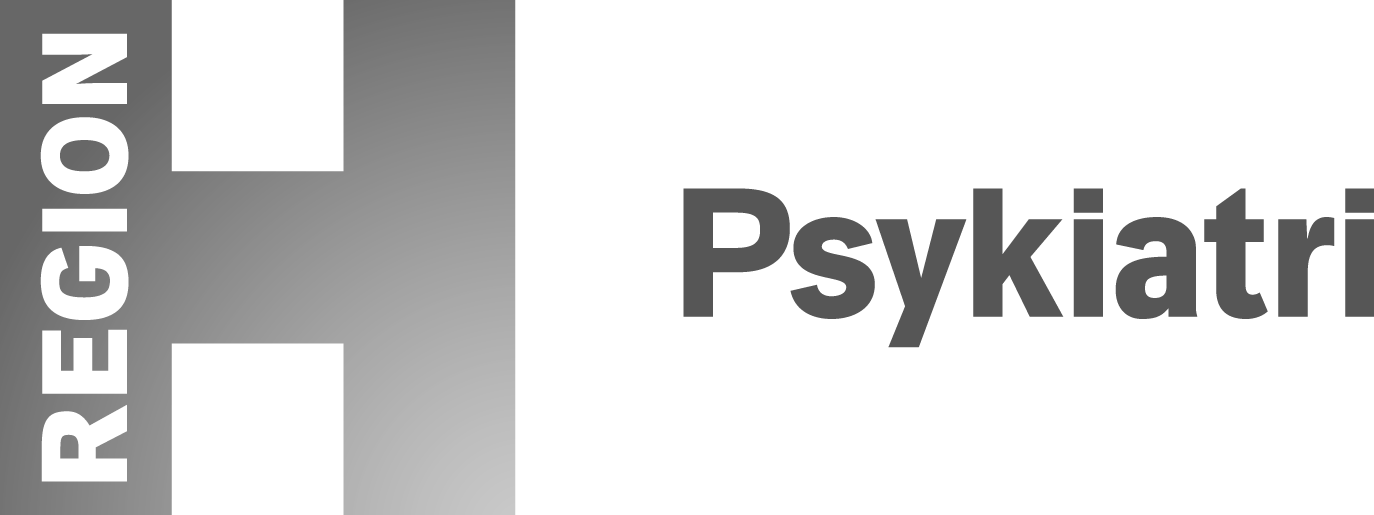
**
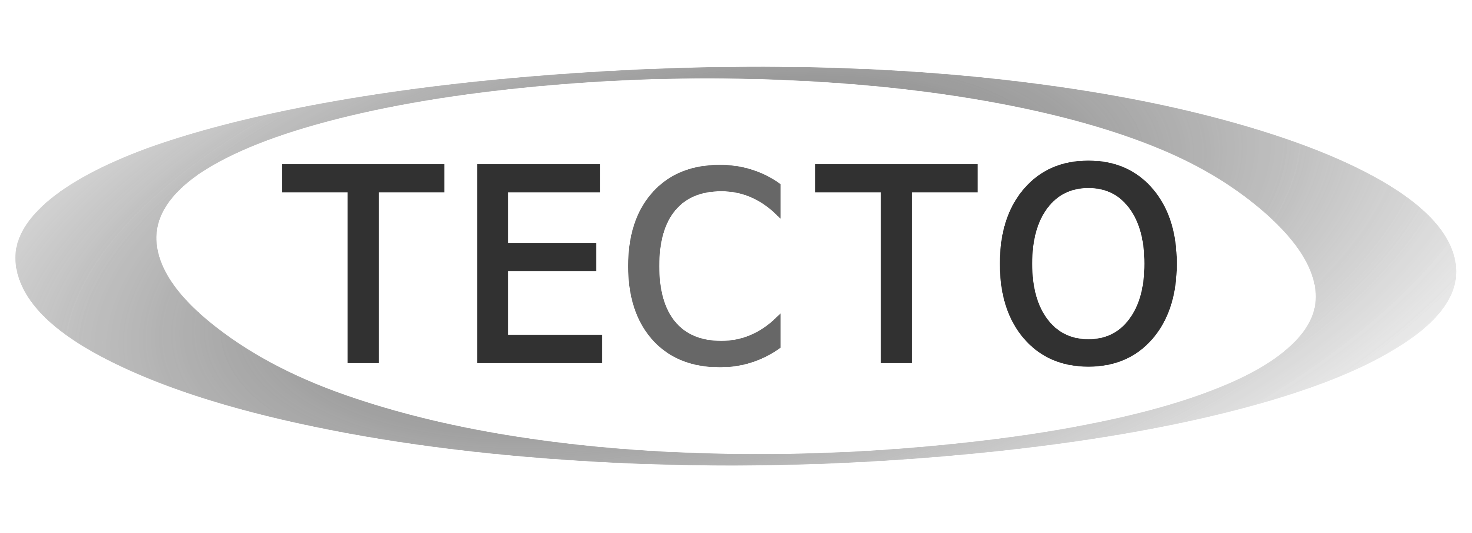


Information on participation in a research project about OCD

*15-17-year-olds*


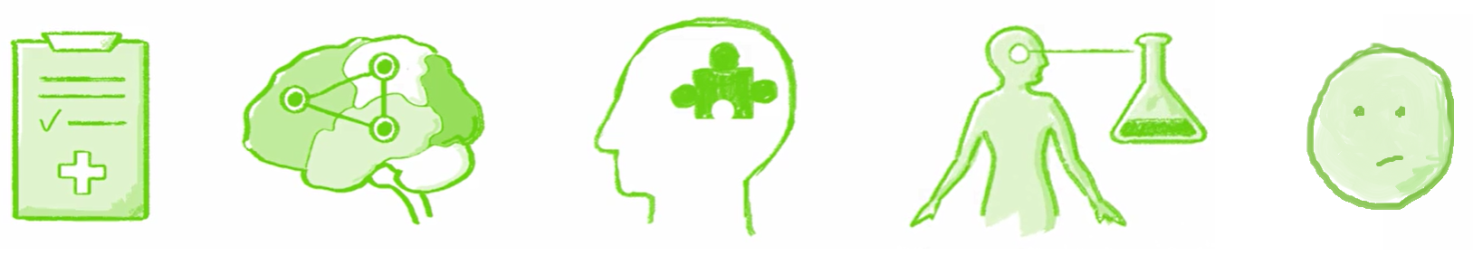

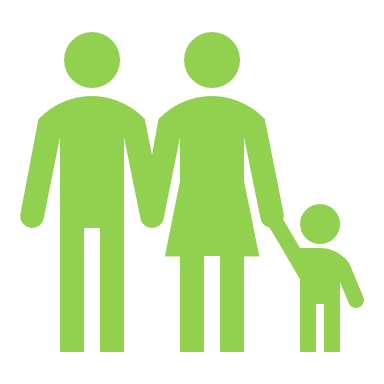


## Information on participation in OCD research project

Project title: TECTO Trial

**FOR YOUNG PEOPLE 15-17 YEARS**

You have been referred because of symptoms of OCD (Obsessive-Compulsive Disorder) and have been assessed at the Child and Adolescent Psychiatric Centre, Bispebjerg. Based on the results of the examination, you have been diagnosed with OCD. We would therefore like to ask you if you would like to participate in a scientific study of family-based therapy for children and adolescents with OCD. The research project is being carried out by the Child and Adolescent Psychiatric Centre, Capital Region, in collaboration with the MR Scanning Department at Hvidovre Hospital.

Before you decide whether to take part in the study, you need to fully understand what the study is about and how we conduct it. We therefore ask you to read this Participant Information carefully.

The aim of the project is to gain knowledge that can improve treatment for children and adolescents with OCD.

If you and your parents decide that you would like to take part in the trial, we will ask your parents to sign a consent form. Remember that you have the right to a period of reflection before deciding whether you want the consent form signed by your parents.

Participation in the trial is voluntary. You and your parents can withdraw consent at any time without giving a reason. It will not affect your further treatment if you refuse the study or if you withdraw from the study during the process.

#### Purpose of the study

In our project, we will investigate the effects of a family-based talk therapy and which brain areas and brain processes the therapy affects in children and adolescents. We will do this by comparing the form of therapy with another form of family-based therapy. We will also look at OCD symptoms, family life, your quality of life and how you function in everyday life.

#### Participation in the project

If you agree to take part in the study, you will receive one of two types of therapy, both of which consist of therapeutic sessions with one of our therapists. The therapy consists of 14 sessions spread over 16 weeks. The therapists are all trained in the two types of therapy and are either psychologists or psychiatric specialists. Both therapies include training in OCD and home exercises. In addition, the therapist will give your parents ongoing guidance on how best to support you. The two therapies are similar in most respects, but differ in the psychological approach that will be reflected in the exercises. It is this difference that we want to explore. Research suggests that both therapies are effective in treating OCD . We have chosen not to name the therapies, as this will make it easier to keep the treatments secret from our researchers, so that they can record as neutral measurements as possible. The study will end after the 16 weeks, regardless of how many sessions you have attended.

During the course there will be a number of research studies. In the section "Research studies and programme" on pages 3-5, you can read more about each study and when it will take place. Six months after the end of the therapy programme , we will ask you and your parents to come back for a follow-up study.

#### The study

The study is a randomized trial. This means that the type of treatment we offer you will be randomly assigned. The random allocation helps to ensure the quality of the project, and you therefore cannot choose which treatment you will receive in the project. If we assess that further treatment is needed after the trial, you will be offered to continue with regular treatment.

It is planned to include 128 children and adolescents aged 8 to 17 who have OCD and 256 parents of children and adolescents with OCD. It will also include 128 children and adolescents of the same age who do not have OCD and 256 parents of children and adolescents without OCD. We are doing this because we want to compare children and young people who do not have OCD with children and young people who do have OCD. Similarly, we will compare parents of children and adolescents who have OCD with parents of children and adolescents without OCD.

#### Other treatment options

If you do not wish to participate in the project, this will not affect your entitlement to current or future treatment. If you choose to participate, you can withdraw from the project at any time. If you withdraw from the project, you will still be entitled to receive the treatment you would have received if you had not participated in the project. So you do not lose any rights as a patient by participating in the project.

In the table below, we have outlined the main differences between receiving treatment in our research project and receiving the usual treatment in the clinic. It is important to stress that the content of the usual treatment for OCD may vary. We cannot therefore guarantee that your course of treatment will be within the outlined framework if you start usual care in the clinic.

|  | **OCD research project** | **Common treatment** |
| --- | --- | --- |
| **Number of sessions** | 14 sessions | Varies according to severity and effect of treatment |
| **Duration of session** | 75 min. | Typically 45-60 min. |
| **Time of session** | Typically fixed weekly sessions spread over 16 weeks.  Open Mon-Fri: 8-16 | Typically fixed weekly sessions  Open Mon-Fri: 8-16 |
| **Format** | Individual therapy  Both parents participate in 5 full sessions  At least one parent attends the last 30 minutes. in the remaining sessions | Individual or group therapy  Parents are involved to varying degrees depending on the age of the child/young person |
| **Research activity** | Total time spent by trial participant is approximately 29 hours. For details see page 4 | No |
| **Location** | Primarily B193/B195, Child and Adolescent Psychiatric Centre, Bispebjerg  And 2 days at MR scanning department, Hvidovre Hospital | Child and Adolescent Psychiatric Centre in your associated ward |
| **Remuneration** | Transport: compensation for research activities  Gift card: given per long day of study (see page 8 - Compensation) | No |

#### Absence or interruption of the trial

Participation in the study is voluntary and if you take part, you and your parents can withdraw consent *at any time*. If you withdraw consent, it will help the research if you tell us the reason, otherwise it may affect our results, but if you do not want to tell us why, please do not do so.

It is also voluntary whether you want to receive information about your examination results, except for MRI scans. If you consent to us taking an MRI scan of your brain, we are obliged to inform you of the results if we detect abnormal changes in your brain (see section on risks and drawbacks). However, this will rarely be the case. If you do not wish to receive information about abnormal changes in your brain, you can opt out of having an MRI scan. You can still be included in the study even if you do not want to be scanned.

You will be closely monitored during the study and treatment, and if we decide that the project's treatment is unsuitable for you or you get much worse, we will withdraw you from the project to find a treatment that is better suited to you. If you fail to attend three consecutive scheduled sessions without cancelling, we will bring forward the final tests and then end the study. You will then continue in regular treatment if you and your family wish and there is a need for it.

#### Research studies and programme

In addition to the treatment, you will take part in various studies with the researchers, where we measure the effect of the therapy. The figure on page 6 gives you an overview of when you will have to take part in studies and for how long. In the following we will go through the types of studies you will be taking part in.

**Parental participation**

Both therapies are family-based, so it is important that parents are present at each session. In five of the sessions, parents must be present for the entire session and in the remaining sessions, parents must be present for the last 30 minutes of the session. It is preferable that both parents attend, but as we realise that it can be difficult to fit this into everyday life, it is also perfectly fine if it is just one parent. If possible, please allow your parents to schedule the same parent to attend the sessions (with the exception of MR test days).

**Tasks and questionnaires**

Before you start treatment and after you are discharged from treatment, you will have to complete different types of tasks designed to test problem-solving strategies, attentional function and memory. We will also ask you and your parents to answer various questionnaires about symptoms of OCD, family life, your quality of life and how you function in everyday life.

**Magnetic resonance imaging (MRI) of the brain**

During an MRI scan, we take pictures of your brain while you perform computer tasks.

An MRI scanner consists of a large tubular magnet. You lie on a tray, which is driven into the tube. Before the scan, we will inform you about what you will do inside the scanner and the whole process will be practised in a so-called MOCK scanner (scanner simulator) so that you are prepared for what will happen. The person doing the scan will be in the next room, and throughout the examination you will be able to get in touch with the staff by pressing a rubber ball. On the test day before, you will try a virtual reality version of being MRI scanned.

We perform the MR scans at the MR department (section 340B) at Hvidovre hospital. We can arrange a taxi for you.

**Interviews**

During the course of treatment, you will be interviewed by healthcare professionals about the progress of your OCD symptoms and any unwanted symptoms of treatment.

**Puzzles**

Four times during the programme, you and one of your parents will be asked to complete different difficult puzzles. We will also measure your and your parent's heart rate before, during and after the puzzle.

**Saliva tests**

You and your parents will be asked to take saliva samples (max 2.8 mililitres each), four times during the course. We will use the saliva samples to measure levels of the hormone oxytocin, which is thought to be important for bonding between people. When saliva sampling, you will be asked to chew on cotton wool for 60 seconds. It is important that you do not eat 2 hours before or drink anything half an hour before the samples are taken.

**Obtaining investigation information**

To avoid you having to do the same tests several times, we will collect information from examinations, tests and video recordings collected during your assessment in the department. If it has been less than a year since you have been in psychiatric care, it will sometimes be necessary to collect data from individual tests carried out in previous care.is because individual tests are not meaningful if they are repeated within a year.

**Video recording**

We film all sessions with the therapist, as well as tests and interviews, so that researchers and clinical staff can ensure the quality of treatment and research data.

Tasks, questionnaires, interviews, puzzles and saliva tests (5 hours)

)

MR scan (3 hours)

**DAY 1**

**DAY 2**

**Starting treatment**

Questionnaires (10 min.)

Interviews and questionnaires (2 hours)

)

))

Interviews, questionnaires and saliva tests (2 hours)

Questionnaires (15 min.)

Interviews (3½ hours)

**Treatment completed**

Tasks, questionnaires, interviews, quizzes and saliva tests (5 hours)

MR scan (3 hours)

Interviews, questionnaires, puzzles and saliva tests (2 hours)

**6 months**

**Studies in progress week**

**1**

**4**

**8**

**15**

**16**

**DAY 1**

**DAY 2**

**SURVEY PROGRAM**

**Study programme**

In the figure below, you can see when the different examinations take place and how long they last.

#### Risks and disadvantages

Please note that if you choose to participate in the trial, you may experience absences from school or work for research activities and therapy sessions. We have described the risks associated with the different parts of the research below.

**Therapy, interviews and research studies**

There are no known risks associated with the two types of therapy or the studies in which you participate.

**MR scan**

There are no known risks associated with MR scanning. The scan is painless and cannot be felt in the body. The scanner uses radio waves and a magnetic field to take the images.

As the MRI scanner contains a strong magnetic field, it is important that you tell us if you:

- has metal objects implanted in the body (e.g. pacemaker or insulin pump)
- has metal splinters in the eye
- has metal bracket for teeth straightening on the teeth
- is pregnant

Individuals may experience stress and claustrophobic discomfort. If you experience claustrophobia or other discomfort during the scan, we will take you out of the scanner immediately.

In rare cases, we may detect abnormal changes on the scan images of your brain that are not known to you beforehand. Only very rarely will these be changes that have a health significance, but if they are, it may affect your future ability to take out private health or life insurance. You should therefore be aware that if you *do not* want information about any abnormal changes found in your brain, and you *do not* want us to pass this information on to your own doctor and/or the relevant hospital department, you should decline to have an MRI scan.

We would like to emphasise that the MRI scan is for research purposes and is not a diagnostic scan. Therefore, you should not consider the scan as a health check.

**New knowledge on risks and drawbacks**

We do not expect any risks for you by participating in the study. You should be aware that there may always be unforeseen risks and stresses associated with participation in health science research. Therefore, although there are no known risks associated with the study, unexpected risks can never be completely ruled out. Please let us know if you experience any health problems during the study. Of course, if we discover new possible side effects during the course of the study that we have not already told you about, you will be informed immediately and you will have to decide whether you want to continue in the study. If, contrary to expectations, you suffer injuries as a result of taking part in the research project, you can seek compensation under the Patient Insurance Act. The project has been approved by the Scientific Ethics Committees of the Capital Region (protocol no. H-18010607).

#### Anonymity, data retention and data processing

All information about you obtained through the research project is covered by the rules on confidentiality. By consenting, you allow your results to be included in the research in an anonymised form. Any material that can be identified (e.g. name or video recording) will be stored anonymously with a code. This code is only available to the research unit at BUC and the Copenhagen Trial Unit, Rigshopitalet, Region Hovedstaden, which is responsible for data storage. Thus, only the project staff can recognise the individual participant. You have the possibility to obtain access to the file according to the rules of the Public Access Act.

We will keep the code to identify the anonymised data until the time when the data processing ends. This includes data on gender and age, which will be used for the statistical analyses. As we would like to be able to contact you during the study and contact you about the study results or anything else afterwards, we will keep your name, phone number and address. We will also have video recordings of the investigation, examinations and treatment available until the end of the data processing.

Data will be stored by Copenhagen Trial Unit, Rigshopitalet, Region Hovedstaden. We store video recordings and data from the various tests confidentially in protected folders on the Capital Region's server. MR images are stored confidentially in protected folders on the DRCMR (Danish Research Center for Magnetic Resonance) server. We collect saliva samples at Bispebjerg Hospital and transport them to a research biobank at the Clinical Biochemistry Department, Rigshospitalet Glostrup, where they are stored in a locked freezer in a locked room. We follow the rules of the Danish Data Protection Authority, and the storage is notified and approved by the Danish Data Protection Authority.

Our plan is to include participants from summer 2018 and three years onwards. The last participant will finish in the summer of 2022, after which we will analyse the results. We expect to have completed analyses and work on publishing scientific papers during 2022. In case of any delays, we will complete data processing by 01/04 2027. All personally identifiable samples and data, except saliva samples, will be destroyed at the end of the study. Saliva samples will be kept for a maximum of six months after the last participating family's samples have been collected. They will then be destroyed. The samples can only be used in a new research project if a scientific ethics committee gives permission.

During the course of your treatment, information may be passed on to the therapist in the department responsible for the case, when your therapist or the research team considers it important for your treatment. For example, this may be for the purpose of further assessment or treatment. If you do not want this, you can opt out in the consent form.

#### Compensation

To compensate you for the time you spend on the project, we offer you 5 gift vouchers of 250 DKK each (1250 DKK in total) to www.sendentanke.dk. We give one gift voucher for each of the long test days before and after the treatment. This amount is taxable (remember to report this on your annual statement to the tax authorities). If you have a free card, it will not matter unless you earn more in total than the free card amount in a year.

#### Initiative and economic conditions

The initiator of the project is the former head of the research unit at the Child and Adolescent Psychiatric Centre of the Capital Region, Professor Kerstin Plessen, employed at the University of Copenhagen. Professor and senior physician Anne Katrine Pagsberg, employed at the same institution, is leading the project.

The project is funded by the Research Pool of the Capital Region of Denmark Psychiatry (1,659,000), the Capital Region of Denmark Research Fund (DKK 1,475,000), the Lundbeck Foundation (DKK 1,575,000, ref.: R191-2015-922 and DKK 390,000, ref.: R211-2015-3990), the Gangsted Foundation (DKK 216,000, ref.R433-A29811), Holm's Memorial Grant (DKK 86,533, ref.: 20006-1951), Sofus Carl Emil Friis and Olga Friis' Wife Grant (DKK 507,736), Psychiatric Research Fund of 1967 (DKK 50,000), Network for Research and Quality Assurance in Psychotherapy and Child and Adolescent Psychiatric Centre (DKK 10,000). We continue to seek additional funding for the project. The investigators have no financial ties to funders.

#### Complaints

If you are subjected to unsatisfactory treatment, you have the possibility to complain, and we will assist you with further information and relevant forms.

#### Access to trial results

We expect the research project to be completed by the end of 2022. The results of the research project will be published in international scientific journals and in the media and may thus help to provide a basis for improving the treatment of OCD internationally.

If you would like to receive information about the results of the research project once it is completed, you can tick a box on the consent form.

We hope that this information has given you enough insight into what it means to participate in the trial and that you feel equipped to make a decision about your possible participation. We also ask you to read the attached material "The rights of the subject in a research project".

If you want to know more about the project, please contact:

Project coordinator Sofie Heidenheim Christensen, Research Unit BUC, Department Bispebjerg, Bispebjerg Bakke 30, 2400 Copenhagen

Tel: 20543576

Mail: [rhp-tecto@regionh.dk](mailto:rhp-tecto@regionh.dk)

Yours sincerely,

Anne Katrine Pagsberg

Professor, Senior Physician, PhD

Region Hovedstadens Psykiatri, Børne- og Ungdomspsykiatrisk Center and University of Copenhagen

Mail: Anne.Katrine.Pagsberg@regionh.dk

##
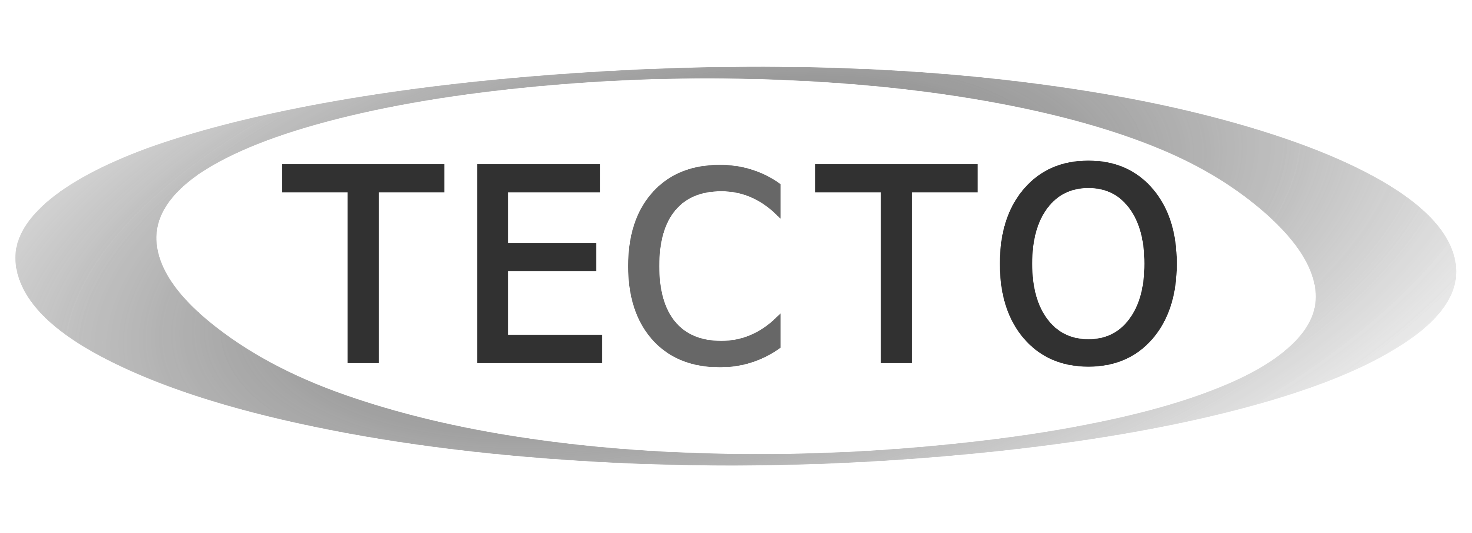


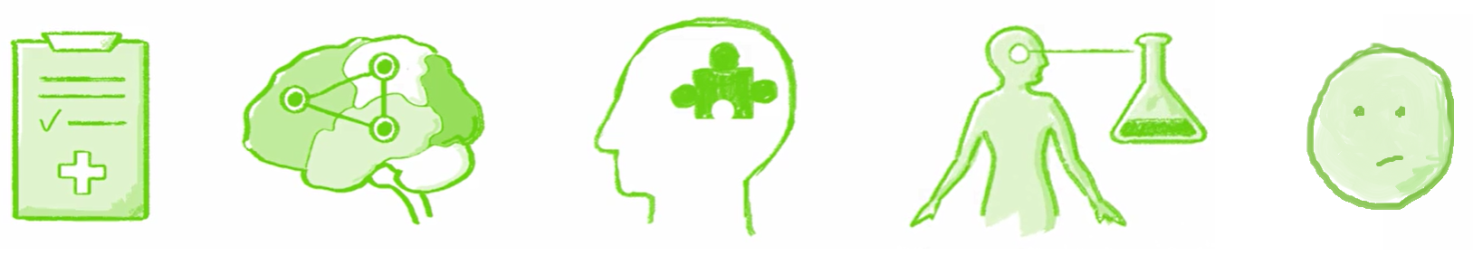

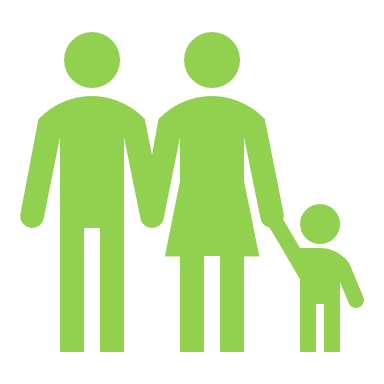


Information on participation in a research project about OCD

*Parental participation*

**
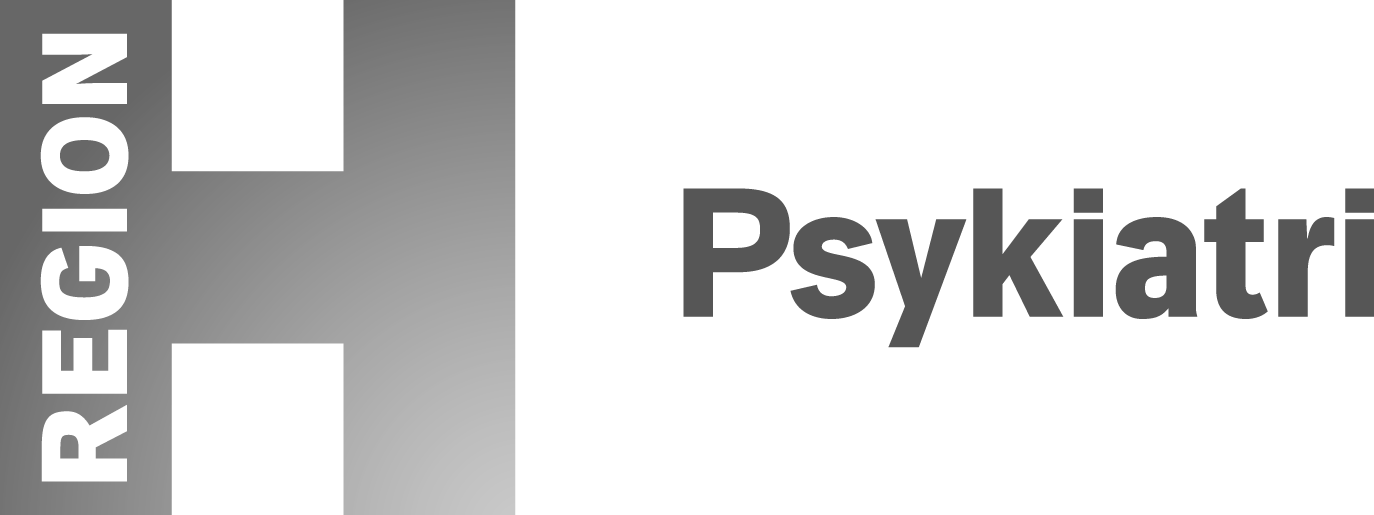
**

## Parental participation in OCD research project

Project title: TECTO Trial

**TO PARENTS**

Your child has been referred for symptoms of Obsessive-Compulsive Disorder (OCD) and has been assessed at the Child and Adolescent Psychiatric Centre, Bispebjerg. Based on the results of the examination, your child has been diagnosed with OCD. Subsequently, you and your child have been offered to participate in our research project on the treatment of OCD. You can find information about the study and the treatment offers in the information material provided, which is entitled *"Information about participation in the OCD research project"****.*** The project includes parental participation and you will therefore receive information material specifically about it.

Before you decide whether to take part in the study, we will inform you about what the study is about and how we are conducting the survey. We will therefore ask you to read the participant information carefully.

If you decide that you would like to take part in the trial, we will ask you to sign a consent form. Remember that you have the right to a period of reflection before deciding whether to sign the consent form.

Participation in the trial is voluntary. You can withdraw your consent at any time and without giving a reason. It will not affect your child's further treatment if you refuse the study or if you withdraw from the study during the process.

#### Objectives of the project

The aim of the project is to gain knowledge that can improve the treatment of children and adolescents with OCD. In order to study the effect of the treatment, we are interested in collecting information from you, the parents. We plan to include 128 children and adolescents aged 8 to 17 who have OCD and 256 parents of children with OCD. It will also include 128 children and young people of the same age who do not have OCD, and 256 parents of children without OCD. We are doing this because we want to compare children and young people who do not have OCD with children and young people who do have OCD. Similarly, we will compare parents of children who have OCD with parents of children without OCD.

#### Participation in the project

Your child will receive therapy in the research project, and both forms of therapy are family-based. It is therefore important that parents are present at each session. In five of the sessions, parents must be present for the entire session and in the remaining sessions, parents must be present for the last 30 minutes of the session. It is preferable that both parents attend, but as we realise that it can be difficult to fit this into everyday life, it is also perfectly fine if it is just one parent. Parents are involved in the treatment so that you can learn how best to support your child through the treatment process.

During the course of treatment, there are five examination days in connection with the therapy. If possible, you should plan for the same parent to attend the sessions. On one day, you as a parent will fill in some questionnaires while your child is being interviewed about his or her symptoms. As a parent, you will have to take part in interviews about the course of treatment.

You will be asked to take saliva samples of a maximum size of 2.8 millilitres each, four times during the course. We will use the saliva samples to measure hormone levels. When taking saliva samples, you will be asked to chew on cotton wool for 60 seconds. It is important that you do not eat 2 hours before or drink anything half an hour before the samples are taken.

In addition, you will be asked to assist your child in completing various difficult puzzles four times during the programme. We will also measure yours and your child's heart rate before, during and after the puzzle.

It is voluntary for you to complete questionnaires, participate in interviews and puzzles, and provide saliva samples, and if you do, you can opt out and withdraw consent *at any time*.

#### Risks and disadvantages

There are no known risks associated with the studies you are participating in.

**Video recording**

We videotape all sessions with the therapist, tests and interviews so that researchers and clinical staff can ensure the quality of treatment and research data.

#### Study programme

In the figure below, you can see when the different examinations take place and how long they last.

Tasks, questionnaires, interviews, puzzles and saliva tests (5 hours)

)

MR scan (3 hours)

**DAY 1**

**DAY 2**

**Starting treatment**

Questionnaires (10 min.)

Interviews and questionnaires (2 hours)

)

))

Interviews, questionnaires and saliva tests (2 hours)

Questionnaires (15 min.)

Interviews (3½ hours)

**Treatment completed**

Tasks, questionnaires, interviews, puzzles and saliva tests (5 hours)

MR scan (3 hours)

Interviews, questionnaires, puzzles and saliva tests (2.5 hours)

**6 months**

**Studies in progress week**

**1**

**4**

**8**

**15**

**16**

**DAY 1**

**DAY 2**

**SURVEY PROGRAM**

#### Anonymity, data retention and data processing

All information obtained through the research project is subject to the rules of confidentiality. By giving your consent, you allow your results to be included in the research in an anonymised form. All material that can be identified (e.g. name) will be stored anonymously with a code. This code is only available to the research unit at BUC and the Copenhagen Trial Unit, Rigshopitalet, Region Hovedstaden, which is responsible for data storage. Thus, only the project staff can recognise the individual participant. You have the possibility to obtain access to the file according to the rules of the Public Access Act.

We will keep the code to identify the anonymised data until the time when the data processing ends. As we would like to be able to contact you during the study and to contact you about the study results (if you wish) or anything else afterwards, we will keep your name, telephone number and address.

Data will be stored by the Copenhagen Trial Unit, Rigshopitalet, Region Hovedstaden. We collect saliva samples at Bispebjerg Hospital and transport them to a research biobank at the Clinical Biochemistry Department, Rigshospitalet Glostrup. We follow the rules of the Danish Data Protection Authority and the storage is notified and approved by the Danish Data Protection Authority.

Our plan is to include participants from summer 2018 and three years onwards. The last participant will finish in the summer of 2022, after which we will analyse the results. We expect to have completed analyses and work on publishing scientific papers during 2022. In case of any delays, we will complete data processing by 01/04 2027. All personally identifiable samples and data, except saliva samples, will be destroyed at the end of the study. Saliva samples will be kept for a maximum of six months after the last participating family's samples have been collected. They will then be destroyed. The samples can only be used in a new research project if a scientific ethics committee gives permission.

#### Initiative and economic conditions

The initiator of the project is the former head of the research unit at the Child and Adolescent Psychiatric Centre of the Capital Region, Professor Kerstin Plessen, employed at the University of Copenhagen. Professor and senior physician Anne Katrine Pagsberg, employed at the same institution, is leading the project.

The project is funded by the Research Pool of the Capital Region of Denmark Psychiatry (1,659,000), the Capital Region of Denmark Research Fund (DKK 1,475,000), the Lundbeck Foundation (DKK 1,575,000, ref.: R191-2015-922 and DKK 390,000, ref.: R211-2015-3990), the Gangsted Foundation (DKK 216,000, ref.R433-A29811), Holm's Memorial Grant (DKK 86,533, ref.: 20006-1951), Sofus Carl Emil Friis and Olga Friis' Wife Grant (DKK 507,736), Psychiatric Research Fund of 1967 (DKK 50,000), Network for Research and Quality Assurance in Psychotherapy and Child and Adolescent Psychiatric Centre (DKK 10,000). We continue to seek additional funding for the project. The investigators have no financial ties to funders.

#### Complaints

If you are subjected to unsatisfactory treatment, you have the possibility to complain, and we will assist you with further information and relevant forms.

**Access to trial results**

We expect the research project to be completed by the end of 2022. The results of the research project will be published in international scientific journals and in the media and may thus help to provide a basis for improving the treatment of OCD internationally.

If you would like to receive information about the results of the research project once it is completed, you can tick a box on the consent form.

We hope that this information has given you enough insight into what it means to participate in the trial and that you feel equipped to make a decision about your possible participation. We also ask you to read the attached material "The rights of the subject in a research project".

If you want to know more about the project, please contact:

Project coordinator Sofie Heidenheim Christensen, Research Unit BUC, Department Bispebjerg, Bispebjerg Bakke 30, 2400 Copenhagen

Tel: 20543576

Mail: [rhp-tecto@regionh.dk](mailto:rhp-tecto@regionh.dk)

Yours sincerely,

Anne Katrine Pagsberg

Professor, Senior Physician, PhD

Region Hovedstadens Psykiatri, Børne- og Ungdomspsykiatrisk Center and University of Copenhagen

Mail: Anne.Katrine.Pagsberg@regionh.dk

## Information on participation as a healthy control person in an OCD research project

Project title: TECTO Trial - Treatment Effects of family based Cognitive Therapy in children and adolescents with Obsessive compulsive disorder

**FOR YOUNG HEALTHY CONTROL PERSONS, 15-17 YEARS**

We would like to ask you if you want to to participate in a scientific study at the Child and Adolescent Psychiatric Centre, Capital Region.

Before you decide whether to take part in the study, you need to fully understand what the study is about and how we conduct it. We therefore ask you to read this Participant Information carefully.

The aim of the project is to gain knowledge that can improve treatment for children and adelescents with Obsessive-Compulsive Disorder (OCD).

If you decide to take part in the trial, we will ask your parents to sign a consent form. Remember that you have the right to a period of reflection time before deciding whether to sign the consent form.

Participation in the trial is voluntary. You and your parents can withdraw consent at any time without giving a reason.

#### Purpose of the study

The aim of the study is to compare the cognitive development of children and adolescents without OCD with children and adolescents who have been diagnosed with OCD and are receiving psychotherapy as treatment. You will be included as a healthy control person in the study and will be compared with children and adolescents with OCD on a series of tests. The project has been approved by the Ethics Committee of the Capital Region (protocol no. H-18010607).

#### Participation in the project

If you agree to take part in the study, you and your parents will have to attend five test days in total.

The children and adelescents with OCD participating in the trial will receive psychotherapy over 4 months. You will be tested at times similar to just before treatment begins and just after treatment ends. You can read more about the tests in the "Plan of the trial" section.

#### Exclusion from and interruption of the trial

Participation in the study is voluntary and if you take part, you and your parents can withdraw your consent *at any time*. If you withdraw your consent, it will benefit the research if you tell us the reason as it may affect our outcome measures, but if you do not want to tell us why, please do not do so.

It is also voluntary whether you want to receive information about your test results, except in the case of MRI, where we are obliged to inform you of the results if we detect abnormal changes in your brain (see section on risks and drawbacks). However, this is extremely rare.

To participate in the trial, you must meet the following criteria:

- No current or previous psychiatric diagnoses
- Intelligence quotient > 70

On the first test day, we will decide whether you can be included. If you cannot be included, you will still be compensated with a gift voucher of 250 DKK for your participation in the first test day.

#### Plan for the experiment

We plan on including 128 children and adolescents aged 8 to 17 with OCD, 256 parents of children and adolescents with OCD, 128 children and adolescents of the same age without OCD, and 256 parents of children and adolescents without OCD. The study will run from summer 2018 to winter 2021.

In the figure at the top of page 4, you can see an overview of how the surveys are distributed over the test days and how long each test day lasts approximately.

In the following, we will review the types of surveys that you and your parents will participate in.

**Interview**

For a start, health professionals will conduct a structured interview in which you and your parents will be asked about any symptoms of the most common psychiatric disorders in children and adolescents.

**Tasks and questionnaires**

You will have to solve different types of tasks aimed at examining problem-solving strategies, attention function and memory. We will also ask you and your parents to complete questionnaires about quality of life, family life and how you are functioning in everyday life.

**Magnetic resonance (MR) brain scan**

During an MRI scan, we will take pictures of your brain while you perform computer tasks.

An MR scanner consists of a large magnet shaped like a tube. You will lie on a tray, which is driven into the tube. Before the scan, we will inform you about what you will be doing inside the scanner, and the process will be practised a few times in a so-called MOCK scanner (scanner simulator) so that you are sure of what is going to happen. The person doing the scan will be in a room next door and will be in contact with you throughout the scan via an intercom. On the test day before, you will try a virtual reality version of being MRI scanned.

We perform the MR scans at the MR department (section 340B) at Hvidovre Hospital. We can arrange a taxi for you and your parents.

**Puzzles**

During the process, you and one of your parents will be asked to complete various difficult puzzles. We will also measure your and your parent's heart rate before, during and after the puzzle.

**Saliva tests**

You and your parents will be asked to give saliva samples. We will use the saliva samples to measure levels of hormones. When taking saliva samples, you will be asked to chew on cotton wool for 60 seconds. It is important that you do not eat 2 hours before or drink anything half an hour before the samples are taken.

**Video recording**

We videotape some of the studies so that researchers and clinical staff can ensure the quality of the research data.

**Interview and tasks for child (3,5 hours)**

**Tasks for child, puzzles, questionnaires and saliva tests (4 hours)**

**DAY 1**

**DAY 2**

**MR scan of child**

**(3 hours)**

**DAY 3**

**SURVEY PROGRAM**

**Startup**

**4 months**

**after start-up**

**Tasks for child, puzzles, questionnaires and saliva tests (4 hours)**

**MR scan of child**

**(3 hours)**

**DAY 1**

**DAY 2**

#### Risks and disadvantages

We have described the risks associated with the different parts of the research below.

**Interviews and research studies**

There are no known risks associated with the studies you are participating in.

**MR Scanning**

There are no known risks associated with MRI. The scan is painless and cannot be felt in the body. The scanner uses radio waves and a magnetic field to take the images.

As the MR scanner contains a strong magnetic field, it is important that you tell us if you:

- have metal objects implanted in your body (e.g. pacemaker or insulin pump)
- have metal splinters in your eye
- have a metal bracket for teeth straightening on your teeth
- are pregnant

Individuals may experience stress and claustrophobic discomfort. If you experience claustrophobia or other discomfort during the scan, we will take you out of the scanner immediately.

Since we see a picture of your brain, in rare cases we can detect abnormal changes that are not already known. Only very rarely these changes will have a health impact, but if they do, it may affect your future ability to take out private health or life insurance. You should therefore be aware that if you *do not* want information about any abnormal changes found in your brain, and you *do not* want us to pass this information on to your own doctor and/or the relevant hospital department, you should decline to have an MRI scan.

We would like to stress that the MRI scan is for research purposes and is not a diagnostic scan. Therefore, you cannot consider the scan as a health check.

#### Anonymity, data retention and data processing

All information about you obtained through the research project is covered by the rules on confidentiality. By consenting, you allow your results to be included in the research in an anonymised form. Any personally identifiable materials (e.g. name or video recording) will be stored in a database anonymised by a code. This code is only accessible to the research unit at BUC and the Copenhagen Trial Unit, Rigshopitalet, Capital Region of Denmark, which is responsible for data storage. Thus, only the project staff can recognise the individual participant. You have the possibility to obtain access to the file according to the rules of the Public Access Act.

We will keep the code to identify the anonymised data until the end of the data processing. This includes data on sex and age, which will be used for the statistical analysis. As we would like to be able to contact you during the study and contact you about the study results (if requested) or anything else afterwards, we will keep your name, phone number and address. We will also keep video recordings of the surveys available until the end of the data processing.

Data will be stored by Copenhagen Trial Unit, Rigshopitalet, Capital Region of Denmark. We store video recordings and data from the various tests confidentially in protected folders on the Capital Region's server. MR images are stored confidentially in protected folders on the DRCMR (Danish Research Center for Magnetic Resonance) server. We store saliva samples separately in a research biobank at the Clinical Biochemistry Department, Rigshospitalet Glostrup, in a locked freezer in a locked room. Data protection regulations are followed and the storage is notified and approved by the Data Protection Agency. Inclusion of trial participants will take place from summer 2018 and three years onwards. The last patient will complete his/her course in summer 2022, after which we will analyse the results. We expect to have completed analyses and work on publication of scientific articles during 2022. In case of any delays, we will complete data processing by 01/04 2027. All personally identifiable samples and data, except saliva samples, will be destroyed at the end of the study. Saliva samples will be kept for a maximum of six months after the last sample has been taken, after which they will be destroyed. The samples can only be used in a new research project if authorised by a Scientific Ethics Committee.

#### Compensation

To compensate you for the time you spend on the project, we offer you 5 gift vouchers of 250 DKK (1.250 DKK total) to sendentanke.dk. We will give a 250 DKK gift voucher for each of the 5 test days. This amount is taxable (remember to report this on your annual statement to the tax authorities). If you have a free card, it will not matter unless you earn more in total than the free card amount in a year.

#### Initiative and economic conditions

The initiator of the project is former head of the research unit at the Child and Adolescent Psychiatric Center, Capital Region, Professor and Senior Physician Kerstin Plessen, Head of the project. Professor and Senior Physician Anne Katrine Pagsberg leads the project.

The project is funded by the Research Pool of the Capital Region of Denmark Psychiatry (1,659,000), the Capital Region of Denmark Research Fund (DKK 1,475,000), the Lundbeck Foundation (DKK 1,575,000, ref.: R191-2015-922 and DKK 390,000, ref.: R211-2015-3990), the Gangsted Foundation (DKK 216,000, ref.R433-A29811), Holm's Memorial Grant (DKK 86,533, ref.: 20006-1951), Sofus Carl Emil Friis and Olga Friis' Wife Grant (DKK 507,736), Psychiatric Research Fund of 1967 (DKK 50,000), Network for Research and Quality Assurance in Psychotherapy and Child and Adolescent Psychiatric Centre (DKK 10,000). We continue to seek additional funding for the project. The investigators have no financial ties to funders.

#### Complaints

If you have an unsatisfactory experience during the examination, you have the possibility to complain and we will help you with further information and relevant forms.

#### Access to trial results

We expect the research project to be completed by the end of 2022. The results of the research project will be published in international scientific journals and in the media and may thus contribute to improved treatment of OCD internationally.

If you would like to receive information about the results of the research project once it is completed, you can tick a box on the consent form.

We hope that this information has given you enough insight into what it means to participate in the trial and that you feel equipped to make a decision about your possible participation. We also ask you to read the attached material "The rights of the subject in a research project".

If you want to know more about the project, please contact:

Project coordinator Sofie Heidenheim Christensen, Research Unit BUC, Department Bispebjerg, Bispebjerg Bakke 30, 2400 Copenhagen.

Tel: 20 36 66 78

Yours sincerely

Anne Katrine Pagsberg

Professor, Senior Physician, Ph.D.

Region Hovedstadens Psykiatri, Børne- og Ungdomspsykiatrisk Center and University of Copenhagen.

Mail: Anne.Katrine.Pagsberg@regionh.dk

## Information on participation as a healthy control person in an OCD research project

Project title: TECTO Trial - Treatment Effects of family based Cognitive Therapy in children and adolescents with Obsessive compulsive disorder

**FOR ADOLESCENTS OF LEGAL AGE - 18 YEARS**

We would like to ask you if you want to participate in a scientific study at the Child and Adolescent Psychiatric Centre, Capital Region.

Before you decide whether to take part in the study, you need to fully understand what the study is about and how we conduct it. We therefore ask you to read this Participant Information carefully.

The aim of the project is to gain knowledge that can improve treatment for children and young people with Obsessive-Compulsive Disorder (OCD).

If you decide to take part in the trial, we will ask you to sign a consent form. Remember that you have the right to a period of reflection time before deciding whether to sign the consent form.

Participation in the trial is voluntary. You can withdraw consent at any time without giving a reason.

#### Purpose of the study

The aim of the study is to compare the cognitive development of children and adolescents without OCD with children and adolescents who have been diagnosed with OCD and are receive psychotherapy as treatment.

You will be included as a healthy control person in the study and will be compared to children and adolescents with OCD on a series of tests. The project has been approved by the Ethics Committee of the Capital Region (protocol no. H-18010607).

#### Participation in the project

If you agree to take part in the study, you and your parents will have to attend five test days in total.

The children and adolescents with OCD participating in the trial will receive psychotherapy over 4 months. You will be tested at times similar to just before treatment begins and just after treatment ends. You can read more about the tests in the "Plan of the trial" section.

#### Exclusion from and interruption of the trial

Participation in the survey is voluntary and if you take part, you can withdraw your consent *at any time*. If you withdraw your consent, it will benefit the research if you tell us the reason as it may affect our outcome measures, but if you do not want to tell us why, please do not do so.

It is also voluntary whether you want to receive information about your test results, except in the case of MRI, where we are obliged to inform you of the results if we detect abnormal changes in your brain (see section on risks and drawbacks). However, this is extremely rare.

To participate in the trial, you must meet the following criteria:

- No current or previous psychiatric diagnoses
- Intelligence quotient > 70

On the first test day, we will decide whether your child can be included. If you cannot be included, you will still be compensated with a gift voucher of 250 DKK for your participation in the first test day

#### Plan for the experiment

It is planned to include 128 children and adolescents aged 8 to 17 with OCD and 128 children and adolescents of the same age without OCD. The study will run from summer 2018 to winter 2021.

In the figure at the top of page 4, you can see an overview of how the surveys are distributed over the test days and how long each test day lasts approximately.

In the following, we will review the types of surveys that you and your parents will participate in.

**Interview**

For a start, healthcare professionals will conduct a structured interview in which you and your parents will be asked about any symptoms of the most common psychiatric disorders in children and adolescents.

**Tasks and questionnaires**

You will have to solve different types of tasks aimed at examining problem-solving strategies, attention function and memory. We will also ask you and your parents to answer questionnaires about quality of life, family life and how you are functioning in everyday life.

**Magnetic resonance (MR) brain scan**

During an MRI scan, we take pictures of your brain while you perform computer tasks.

An MR scanner consists of a large magnet shaped like a tube. You will lie on a tray, which is driven into the tube. Before the scan, we will inform you about what you will be doing inside the scanner, and the process will be practised a few times in a so-called MOCK scanner (scanner simulator) so that you are sure of what is going to happen. The person doing the scan will be in a room next door and will be in contact with you throughout the scan via an intercom. On the test day before, you will try a virtual reality version of being MRI scanned.

We perform the MR scans at the MR department (section 340B) at Hvidovre Hospital. We can arrange a taxi for you and your parents.

**Puzzles**

During the process, you and one of your parents will be asked to complete various difficult puzzles. We will also measure your and your parent's heart rate before, during and after the puzzle.

**Saliva tests**

You and your parents will be asked to give saliva samples We will use the saliva samples to measure levels of hormones. When taking saliva samples, you will be asked to chew on cotton wool for 60 seconds. It is important that you do not eat 2 hours before or drink anything half an hour before the samples are taken.

**Video recording**

We videotape some of the studies so that researchers and clinical staff can ensure the quality of the research data.

**Interview and tasks for child (3,5 hours)**

**Tasks for child, puzzles, questionnaires and saliva tests (4 hours)**

**DAY 1**

**DAY 2**

**MR edging of child**

**(3 hours)**

**DAY 3**

**SURVEY PROGRAM**

**Startup**

**4 months**

**after start-up**

**Tasks for child, puzzles, questionnaires and saliva tests (4 hours)**

**MR scan of child**

**(3 hours)**

**DAY 1**

**DAY 2**

#### Risks and disadvantages

We have described the risks associated with the different parts of the research below.

**Interviews and research studies**

There are no known risks associated with the studies you are participating in.

**MR Scanning**

There are no known risks associated with MRI. The scan is painless and cannot be felt in the body. The scanner uses radio waves and a magnetic field to take the images.

As the MR scanner contains a strong magnetic field, it is important that you tell us if you:

- have metal objects implanted in your body (e.g. pacemaker or insulin pump)
- have metal splinters in your eye
- have a metal bracket for teeth straightening on your teeth
- are pregnant

Individuals may experience stress and claustrophobic discomfort. If you experience claustrophobia or other discomfort during the scan, we will take you out of the scanner immediately.

Since we see a picture of your brain, we can in rare cases detect abnormal changes that are not already known in you. Only very rarely will these changes have a health impact, but if they do, it may affect your future ability to take out private health or life insurance. You should therefore be aware that if you *do not* want information about any abnormal changes found in your brain, and you *do not* want us to pass this information on to your own doctor and/or the relevant hospital department, you should decline to have an MRI scan.

We would like to stress that the MRI scan is for research purposes and is not a diagnostic scan. Therefore, you cannot consider the scan as a health check.

#### Anonymity, data retention and data processing

All information about you obtained through the research project is covered by the rules on confidentiality. By consenting, you allow your results to be included in the research in an anonymised form. Any material that can be identified (e.g. name or video recording) will be stored in a database anonymised by a code. This code is only available to the research unit at BUC and the Copenhagen Trial Unit, Rigshopitalet, Capital Region of Denmark, which is responsible for data storage. Thus, only the project staff can recognise the individual participant. You have the possibility to obtain access to the file according to the rules of the Public Access Act.

We will keep the code to identify the anonymised data until the end of the data processing. This includes data on gender and age, which will be used for the statistical analysis. As we would like to be able to contact you during the study and contact you about the study results (if requested) or anything else afterwards, we will keep your name, phone number and address. We will also keep video recordings of the surveys available until the end of the data processing.

Data will be stored by Copenhagen Trial Unit, Rigshopitalet, Capital Region. We store video recordings and data from the various tests confidentially in protected folders on the Capital Region's server. MR images are stored confidentially in protected folders on the DRCMR (Danish Research Center for Magnetic Resonance) server. The rules of the Danish Data Protection Authority are followed and the storage is notified and approved by the Danish Data Protection Authority. Inclusion of trial participants runs from summer 2018 and three years onwards. The last patient will complete his/her course in the summer of 2022, after which we will analyse the results. We expect to have completed analyses and work on publication of scientific articles during 2022. In case of any delays, we will complete data processing by 01/04 2027. All personally identifiable samples and data, except saliva samples, will be destroyed at the end of the study. Saliva samples will be kept for a maximum of six months after the last sample has been taken, after which they will be destroyed. The samples can only be used in a new research project if authorised by a Scientific Ethics Committee.

#### Compensation

To compensate for the time you spend on the project, we are offering an incentive in the form of 5 gift vouchers of 250 DKK (1,250 DKK in total) to www.sendentanke.dk. We will give a 250 DKK gift voucher for each of the 5 test days. This amount is taxable (remember to report this on your annual statement to the tax authorities). If you have a free card, it will not matter unless you earn more in total than the free card amount in a year.

#### Initiative and economic conditions

The initiator of the project is former head of the research unit at the Child and Adolescent Psychiatric Center, Capital Region, Professor and Senior Physician Kerstin Plessen. Head of the research unit, Professor and Senior Physician Anne Katrine Pagsberg leads the project.

The project is funded by the Research Pool of the Capital Region of Denmark Psychiatry (1,659,000), the Capital Region of Denmark Research Fund (DKK 1,475,000), the Lundbeck Foundation (DKK 1,575,000, ref.: R191-2015-922 and DKK 390,000, ref.: R211-2015-3990), the Gangsted Foundation (DKK 216,000, ref.R433-A29811), Holm's Memorial Grant (DKK 86,533, ref.: 20006-1951), Sofus Carl Emil Friis and Olga Friis' Wife Grant (DKK 507,736), Psychiatric Research Fund of 1967 (DKK 50,000), Network for Research and Quality Assurance in Psychotherapy and Child and Adolescent Psychiatric Centre (DKK 10,000). We continue to seek additional funding for the project. The investigators have no financial ties to funders.

#### Complaints

If you have an unsatisfactory experience during the examination, you have the possibility to complain and we will help you with further information and relevant forms.

#### Access to trial results

We expect the research project to be completed by the end of 2022. The results of the research project will be published in international scientific journals and in the media and may thus contribute to improved treatment of OCD internationally. If you would like to receive information about the results of the research project once it is completed, please tick a box on the consent form.

We hope that this information has given you enough insight into what it means to participate in the trial and that you feel equipped to make a decision about your possible participation. We also ask you to read the attached material "The rights of the subject in a research project".

If you want to know more about the project, please contact:

Project coordinator Sofie Heidenheim Christensen, Research Unit BUC, Department Bispebjerg, Bispebjerg Bakke 30, 2400 Copenhagen.

Tel: 20 36 66 78

Yours sincerely

Anne Katrine Pagsberg

Professor, Senior Physician, Ph.D.

Region Hovedstadens Psykiatri, Børne- og Ungdomspsykiatrisk Center and University of Copenhagen.

Mail: Anne.Katrine.Pagsberg@regionh.dk

## Information about your child's participation as a healthy control person in an OCD research project

Project title: TECTO Trial - Treatment Effects of family based Cognitive Therapy in children and adolescents with Obsessive compulsive disorder

**TO PARENTS OF CONTROLS**

We would like to ask you if your child wants to participate in a scientific study at the Child and Adolescent Psychiatric Centre, Capital Region of Denmark.

Before you decide whether your son/daughter should take part in the study, you need to fully understand what the study is about and how we conduct it. We therefore ask you to read this participant information carefully.

The aim of the project is to gain knowledge that can improve treatment for children and young people with Obsessive-Compulsive Disorder (OCD).

If you decide that your son/daughter should take part in the trial, we will ask you to sign a consent form. Remember that you have the right to a period of reflection time before deciding whether to sign the consent form.

Participation in the trial is voluntary. You can withdraw consent at any time without giving a reason.

#### Purpose of the study

The aim of the study is to compare the cognitive development of children and adolescents without OCD with children and adolescents who have been diagnosed with OCD and who receive psychotherapy as treatment.

Your child will be a control in the study and will be compared to children and adolescents with OCD on a series of tests. The project has been approved by the Scientific Ethics Committee of the Capital Region (protocol no. H-18010607).

#### Participation in the survey

If you agree to allow your child to take part in the study, you and your child will have to attend five test days in total.

The children and young people with OCD participating in the trial will receive psychotherapy over 4 months. Your child will be tested at times similar to just before treatment begins and just after treatment ends. You can read more about the tests in the "Plan for the trial" section.

#### Exclusion from and interruption of the trial

Participation in the survey is voluntary and if you take part, you can withdraw your consent *at any time*. If you withdraw your consent, it will be beneficial to the research if you tell us the reason as it may affect our outcome measures, but if you do not want to tell us why, please do not do so.

It is also optional whether you want to receive information about your son/daughter's test results, except for MRI scans, as we are obliged to inform you of the results if we detect abnormal changes in your child's brain (see section on risks and drawbacks). However, this is extremely rare.

To participate in the trial, your child must meet the following criteria:

- No current or previous psychiatric diagnoses
- Intelligence quotient > 70

On the first test day, we will decide whether your child can be included. If your child cannot be included, you will still be compensated with a gift voucher of 250 DKK for your participation in the first test day

#### Plan for the experiment

It is planned to include 128 children and adolescents aged 8 to 17 with OCD, 256 parents of children and adolescents with OCD, 128 children and adolescents of the same age without OCD, and 256 parents of children and adolescents without OCD. The study will run from summer 2018 to winter 2021.

In the figure at the top of page 4, you can see how the surveys are distributed over the test days and how long each test day lasts.

In the following, we will go through the types of studies that you and your child will participate in.

**Interview**

For a start, healthcare professionals will conduct a structured interview in which you and your child will be asked about any symptoms of the most common psychiatric disorders in children and adolescents.

**Tasks and questionnaires**

Your child will have to solve different types of tasks aimed at testing problem-solving strategies, attention function and memory. We will also ask you and your child to complete questionnaires about quality of life, family relationships and how your son/daughter is functioning in everyday life.

**Magnetic resonance (MR) brain scan**

During an MRI scan, we take pictures of your child's brain while he/she performs computer tasks.

An MR scanner consists of a large magnet shaped like a tube. Your child will lie on a tray, which is driven into the tube. Before the scan, we will inform your child about what he/she will do inside the scanner, and the process will be practised a few times in a so-called MOCK scanner (scanner simulator) so that he/she is sure of what is going to happen. The person doing the scan will be in a room next door and will be in contact with your child throughout the scan via an intercom. On the test day before, your child will try a virtual reality version of being MRI scanned.

We perform the MR scans at the MR department (section 340B) at Hvidovre Hospital. We can arrange for a taxi to take you there by prior arrangement.

**Puzzles**

You will be asked to have one parent assist your child in putting together various difficult puzzles. We will also measure your and your child's heart rate before, during and after the puzzle.

**Saliva tests**

You and your child will be asked to give saliva samples. We will use the saliva samples to measure levels of hormones. When taking saliva samples, you will be asked to chew on cotton wool for 60 seconds. It is important that you do not eat 2 hours before or drink anything half an hour before the samples are taken.

**Video recording**

We videotape some of the studies so that researchers and clinical staff can ensure the quality of the research data.

**Interview and tasks for child (3,5 hours)**

**Tasks for child, puzzles, questionnaires and saliva tests (4 hours)**

**DAY 1**

**DAY 2**

**MR scan of child**

**(3 hours)**

**DAY 3**

**SURVEY PROGRAM**

**Startup**

**4 months**

**after start-up**

**Tasks for child, puzzles, questionnaires and saliva tests (4 hours)**

**MR scan of child**

**(3 hours)**

**DAY 1**

**DAY 2**

#### Risks and disadvantages

We have described the risks associated with the different parts of the research below.

**Interviews and research studies**

There are no known risks associated with the studies in which the child participates.

**MR Scanning**

There are no known risks associated with MRI. The scan is painless and cannot be felt in the body. The scanner uses radio waves and a magnetic field to take the images.

As the MRI scanner contains a strong magnetic field, it is important that you tell us if your child:

- has metal objects implanted in the body (e.g. pacemaker or insulin pump)
- has metal splinters in his/her eye
- has a metal bracket for teeth straightening on his/her teeth
- is pregnant

Individuals may experience stress and claustrophobic discomfort. If your child experiences claustrophobia or other discomfort during the scan, we will take your child out of the scanner immediately.

As we look at a picture of your child's brain, we may, in rare cases, detect abnormal changes that are not already known in your child. Only extremely rarely will these changes have a health significance, but if they do, it may affect future opportunities to take out private health or life insurance. You should therefore be aware that if you *do not* want information about any abnormal changes found in your child's brain, and you *do not* want us to pass this information on to your own doctor and/or the relevant hospital department, you must decline to have the child scanned.

We would like to stress that the MRI scan is for research purposes and is not a diagnostic scan. You cannot therefore regard the scan as a health check.

#### Anonymity, data retention and data processing

All information about your child that is obtained through the research project is covered by the rules on confidentiality. By giving consent, you allow your child's results to be included in the research in an anonymised form. Any material that can be identified (e.g. name or video recording) will be stored in a database anonymised by a code. This code is only available to the research unit at BUC and the Copenhagen Trial Unit, Rigshopitalet, Region Hovedstaden, which is responsible for data storage. Thus, only the project staff can recognise the individual participant. You have the possibility to obtain access to the file according to the rules of the Public Access Act.

We will keep the code to identify the anonymised data until the end of the data processing. This includes data on gender and age, which will be used for the statistical analysis. As we would like to be able to contact you during the study and contact you about the results of the study (if requested) or anything else afterwards, we will keep your name, phone number and address. We will also keep video recordings of the surveys available until the end of the data processing.

Data will be stored by Copenhagen Trial Unit, Rigshopitalet, Region Hovedstaden. We store video recordings and data from the various tests confidentially in protected folders on the Capital Region's server. MR images are stored confidentially in protected folders on the DRCMR (Danish Research Center for Magnetic Resonance) server. We store saliva samples separately in a research biobank at the Clinical Biochemistry Department, Rigshospitalet Glostrup, in a locked freezer in a locked room. Data protection regulations are followed and the storage is notified and approved by the Data Protection Agency. Inclusion of study participants will run from summer 2018 and three years onwards. The last participant will complete his/her programme in summer 2022, after which we will analyse the results. We expect to have completed analyses and work on publication of scientific articles during 2022. In case of any delays, we will complete data processing by 01/04 2027. All personally identifiable samples and data, except saliva samples, will be destroyed at the end of the study. Saliva samples will be kept for a maximum of six months after the last sample has been taken, after which they will be destroyed. Samples can only be used in a new research project if a scientific ethics committee gives permission.

#### Compensation

To compensate you for the time you spend on the project, we offer you 5 gift vouchers of 250 DKK (1.250 DKK total) to sendentanke.dk. We will give a 250 DKK gift voucher for each of the 5 test days. This amount is taxable (remember to report this on your annual statement to the tax authorities). If your child has a free card, it will not matter unless he/she earns more than the total amount of the free card in a year.

#### Initiative and economic conditions

The initiator of the project is former head of the research unit at the Child and Adolescent Psychiatric Center, Capital Region, Professor and Senior Physician Kerstin Plessen. Head of the research unit, Professor and Senior Physician Anne Katrine Pagsberg leads the project.

The project is funded by the Research Pool of the Capital Region of Denmark Psychiatry (1,659,000), the Capital Region of Denmark Research Fund (DKK 1,475,000), the Lundbeck Foundation (DKK 1,575,000, ref.: R191-2015-922 and DKK 390,000, ref.: R211-2015-3990), the Gangsted Foundation (DKK 216,000, ref.R433-A29811), Holm's Memorial Grant (DKK 86,533, ref.: 20006-1951), Sofus Carl Emil Friis and Olga Friis' Wife Grant (DKK 507,736), Psychiatric Research Fund of 1967 (DKK 50,000), Network for Research and Quality Assurance in Psychotherapy and Child and Adolescent Psychiatric Centre (DKK 10,000). We continue to seek additional funding for the project. The investigators have no financial ties to funders.

#### Complaints

If you or your child have an unsatisfactory experience during the examination, you have the possibility to complain and we will help you with further information and relevant forms.

#### Access to trial results

We expect the research project to be completed by the end of 2022. The results of the research project will be published in international scientific journals and in the media and may thus contribute to improved treatment of OCD internationally.

If you would like to receive information about the results of the research project when it is completed, you can tick a box on the consent form.

We hope that this information has given you enough insight into what it means to participate in the trial and that you feel equipped to make a decision about your child's possible participation. We also ask you to read the attached material "The rights of the subject in a research project".

We will try to contact you by phone within a few weeks to see if you are interested in letting your child participate in the project. As it is not always possible to find a correct telephone number, we would ask you to contact us if you would like your child to take part in the TECTO project. Of course, you are also very welcome to contact us if you have any questions about the project.

Project coordinator Sofie HeidenHeim Christensen, Research Unit BUC, Department Bispebjerg, Bispebjerg Bakke 30, 2400 Copenhagen.

Tel: 20 36 66 78

Yours sincerely

Anne Katrine Pagsberg

Professor, Senior Physician, Ph.D.

Region Hovedstadens Psykiatri, Børne- og Ungdomspsykiatrisk Center and University of Copenhagen.

Mail: Anne.Katrine.Pagsberg@regionh.dk

## Parental participation as a healthy control person in an OCD research project

Project title: TECTO Trial - Treatment Effects of family based Cognitive Therapy in children and adolescents with Obsessive compulsive disorder

**TO PARENTS OF HEALTHY CONTROL PERSONS**

Your child has been offered to take part in a research project at the Child and Adolescent Psychiatric Centre, Capital Region of Denmark, and we would like to ask you if you want to take part in the same scientific study. The project includes parental participation, and you will therefore receive information material specifically about this.

Before you decide whether to take part in the study, we will tell you what the study is about and how we are conducting it. We will therefore ask you to read the participant information carefully.

If you decide that you would like to take part in the trial, we will ask you each to sign a consent form. Remember that you have the right to a period of reflection before deciding whether to sign the consent form.

Participation in the trial is voluntary. You can withdraw your consent at any time and without giving a reason.

#### Purpose of the study

The aim of the study is to compare the cognitive development of children and adolescents without OCD with children and adolescents who have been diagnosed with OCD and are receiving psychotherapy as treatment. You will be included as controls in the study and will be compared with parents of children and adolescents with OCD. The project has been approved by the Scientific Ethics Committees of the Capital Region (protocol no. H-18010607).

#### Participation in the project

If you agree to let your child take part in the study, you and your child will have to attend five test days in total.

The children and adolescents with OCD participating in the trial will receive psychotherapy over 4 months. You will be asked to complete questionnaires, assist your child with difficult puzzles, have your heart rate measured and give saliva samples at times corresponding to just before treatment begins and just after treatment ends

#### Exclusion from and interruption of the trial

#### Participation in the survey is voluntary and if you take part, you can withdraw your consent at any time. If you withdraw your consent, it will be beneficial to the research if you tell us the reason as it may affect our outcome measures, but if you do not want to tell us why, please do not do so.

#### Plan for the experiment

It is planned to include 128 children and adolescents aged 8 to 17 with OCD, 256 parents of children and adolescents with OCD, 128 children and adolescents of the same age without OCD, and 256 parents of children and adolescents without OCD. The study will run from summer 2018 to winter 2021.

You will be asked to take part in an interview where health professionals will ask you and your child about symptoms of different mental disorders in your child. The interview will be video recorded. In addition, you will be asked to complete a series of questionnaires and give saliva samples. In the figure at the top of page 3, you can see how the tests are distributed over the test days and approximately how long each test day lasts.

**Puzzles**

You will be asked to have one of you assist your child in putting together various difficult puzzles. We will also measure your and your child's heart rate before, during, and after the puzzle.

**Saliva tests**

You and your child will be asked to give saliva samples. We will use the saliva samples to measure levels of hormones. When taking saliva samples, you will be asked to chew on cotton wool for 60 seconds. It is important that you do not eat 2 hours before or drink anything half an hour before the samples are taken.

#### Study programme

In the figure below, you can see when the different examinations take place and how long they last.

**Interview and tasks for child (3 hours)**

**Tasks for child, puzzles, questionnaires and saliva tests (4 hours)**

**DAY 1**

**DAY 2**

**MR scan of child**

**(3 hours)**

**DAY 3**

**SURVEY PROGRAM**

**Startup**

**4 months**

**after start-up**

**Tasks for child, puzzles, questionnaires and saliva tests (4 hours)**

**MR scan of child**

**(3 hours)**

**DAY 1**

**DAY 2**

#### Risks and disadvantages

There are no known risks associated with the studies you are participating in.

#### Anonymity, data retention and data processing

All information obtained through the research project is subject to the rules of confidentiality. By giving your consent, you allow your results to be included in the research in anonymised form. Any material that can be identified (e.g. name or video recording) will be stored in a database anonymised by a code. This code is only available to the research unit at BUC and the Copenhagen Trial Unit, Rigshopitalet, Region Hovedstaden, which is responsible for data storage. Thus, only the project staff can recognise the individual participant. You have the possibility to obtain access to the file according to the rules of the Public Access Act.

We will keep the code to identify the anonymised data until the end of the data processing. This includes data on sex and age, which will be used for the statistical analysis. As we would like to be able to contact you during the study and contact you about the results of the study (if requested) or anything else afterwards, we will keep your name, phone number and address. We will also keep video recordings of the surveys available until the end of the data processing.

Data will be stored by Copenhagen Trial Unit, Rigshopitalet, Region Hovedstaden. We store video recordings and data from the various tests confidentially in protected folders on the Capital Region's server. MR images are stored confidentially in protected folders on the DRCMR (Danish Research Center for Magnetic Resonance) server. We store saliva samples separately in a research biobank at the Clinical Biochemistry Department, Rigshospitalet Glostrup, in a locked freezer in a locked room. Data Protection Authority rules are followed and the storage is notified and approved by the Data Protection Authority. Inclusion of trial participants will take place from summer 2018 and three years onwards. The last participant will complete the programme in the summer of 2022, after which we will analyse the results. We expect to have completed analyses and work on publication of scientific articles during 2022. In case of any delays, we will complete data processing by 01/04 2027. All personally identifiable samples and data, except saliva samples, will be destroyed at the end of the study. Saliva samples will be kept for a maximum of six months after the last sample has been taken. They will then be destroyed. The samples can only be used in a new research project if authorised by a scientific ethics committee.

#### Initiative and economic conditions

The initiator of the project is the former head of the research unit at the Child and Adolescent Psychiatric Centre, Professor Kerstin Plessen. The head of the research unit, Professor Anne Katrine Pagsberg, is leading the project.

The project is funded by the Research Pool of the Capital Region of Denmark Psychiatry (1,659,000), the Capital Region of Denmark Research Fund (DKK 1,475,000), the Lundbeck Foundation (DKK 1,575,000, ref.: R191-2015-922 and DKK 390,000, ref.: R211-2015-3990), the Gangsted Foundation (DKK 216,000, ref.R433-A29811), Holm's Memorial Grant (DKK 86,533, ref.: 20006-1951), Sofus Carl Emil Friis and Olga Friis' Wife Grant (DKK 507,736), Psychiatric Research Fund of 1967 (DKK 50,000), Network for Research and Quality Assurance in Psychotherapy and Child and Adolescent Psychiatric Centre (DKK 10,000). We continue to seek additional funding for the project. The investigators have no financial ties to funders.

#### Complaints

If you experience unsatisfactory treatment, you have the possibility to complain and we will help you with further information and relevant forms.

#### Access to trial results

We expect the research project to be completed by the end of 2022. The results of the research project will be published in international scientific journals and in the media and may thus contribute to improved treatment of OCD internationally.

If you would like to receive information about the results of the research project when it is completed, you can tick a box on the consent form.

We hope that this information has given you enough insight into what it means to take part in the trial and that you feel equipped to make your own decision about whether to participate. We also ask you to read the attached material "The rights of the subject in a research project".

If you want to know more about the project, please contact:

Project coordinator Sofie Heidenheim Christensen, Research Unit BUC, Department Bispebjerg, Bispebjerg Bakke 30, 2400 Copenhagen.

Tel: 20 36 66 78

Yours sincerely

Anne Katrine Pagsberg

Professor, Senior Physician, Ph.D.

Region Hovedstadens Psykiatri, Børne- og Ungdomspsykiatrisk Center and University of Copenhagen.

Mail: Anne.Katrine.Pagsberg@regionh.dk
