# Supplementary material for: Family-based cognitive behavioural therapy versus family-based relaxation therapy for obsessive-compulsive disorder in children and adolescents: protocol for a randomised clinical trial (the TECTO trial)
Source: BMC Psychiatry. 2022 Mar 19;22:204. doi: 10.1186/s12888-021-03669-2 (PMC8933964; doi:10.1186/s12888-021-03669-2)
Supplement: Supplementary file 4 — Additional file 4. [file 12888_2021_3669_MOESM4_ESM.docx]

**TO PARENTS OF PATIENT**

**INFORMED CONSENT FOR PARTICIPATION IN A HEALTH SCIENCE RESEARCH PROJECT**

**Research project title:** TECTO Trial - Treatment Effects of family based Cognitive Therapy in children and adolescents with Obsessive compulsive disorder

**Declaration of the project participant:**

I have received written and verbal information and I know enough about the purpose, method, advantages, and disadvantages to give my consent.

I know that participation is voluntary and that I can always withdraw my consent without losing my current or future rights to treatment.

I consent to participate in the research project. I have received a copy of this consent form and a copy of the written information about the project for my own use.

| I also consent to the following, as a participant in the project: | **Yes** set x | **No** set x |
| --- | --- | --- |
| That there is recorded video of me |  |  |
| That there is recorded audio file of me |  |  |
| Saliva samples are taken from me and stored in a research biobank |  |  |

Project Participant:

Date: Signature:

Would you like to receive information about the results of the research project?

Yes (set x) No (set x)

**Declaration by the person providing the information:**

I declare that the participant has received oral and written information about the trial.

In my opinion the participant has received sufficient information to make an informed decision about participating in the trial.

The name of the person who provided the information:

**TO AN ADULT PARTICIPANT**

**INFORMED CONSENT FOR PARTICIPATION IN A HEALTH SCIENCE RESEARCH PROJECT**

**Research project title:** TECTO Trial - Treatment Effects of family based Cognitive Therapy in children and adolescents with Obsessive compulsive disorder

**Declaration of the project participant who has reached the age of 18:**

I have received written and verbal information and I know enough about the purpose, method, advantages, and disadvantages to give my consent.

I know that participation is voluntary and that I can always withdraw my consent without losing my current or future rights to treatment.

I consent to) participate in the research project. I have received a copy of this consent form and a copy of the written information about the project for my own use.

| I also consent to the following, as a participant in the project: | **Yes** set x | **No** set x |
| --- | --- | --- |
| That there is recorded video of me |  |  |
| That there is recorded audio file of me |  |  |
| That MR scans are done of me |  |  |
| Saliva samples are taken from me and stored in a research biobank |  |  |
| Information is passed on to the case manager during treatment when the therapist or research team consider it important for my treatment |  |  |

Information about the scan:

I understand that I will be informed if any abnormal changes are observed in my brain and that relevant information may be passed on to my own doctor and/or the relevant hospital department, if necessary. I understand that this examination is not a substitute for a regular examination, as the images will not be systematically assessed by a physician. It is therefore important to carry out a clinical examination should I have any symptoms that make this necessary.

Would you like to receive information about the results of the research project?

Yes (set x) No (set x)

Name:

Date: Signature:

**Declaration by the person providing the information:**

I declare that the participant has received oral and written information about the trial.

In my opinion the participant has received sufficient information to make an informed decision about participating in the trial.

The name of the person who provided the information:

Date: Signature:

Project identification: Scientific Committees Protocol No: H-18010607

**TO PARENTS OF PATIENT**

**INFORMED CONSENT FOR PARTICIPATION IN A HEALTH SCIENCE RESEARCH PROJECT**

**Research project title:** TECTO Trial - Treatment Effects of family based Cognitive Therapy in children and adolescents with Obsessive compulsive disorder

**Declaration by the holder of parental responsibility:**

I/we have received written and verbal information and I/we know enough about the purpose, method, advantages, and disadvantages to give my/our consent.

I/we know that participation is voluntary and that I/we can withdraw my/our consent at any time without my/our son/daughter losing his/her current or future rights to treatment.

I/we consent to (child's name) to participate in the research project. I/we have received a copy of this consent form and a copy of the written information about the project for my/our own use.

| I also consent to the following, as a participant and a participant’s parent in the project: | **Yes** set x | **No** set x |
| --- | --- | --- |
| There is recorded video of my/our child and me/us |  |  |
| There is recorded audio file of my/our child me/us |  |  |
| That there is performed MR scans of my/our child |  |  |
| Saliva samples are taken from my/our child and stored in the research biobank |  |  |
| Information is passed on to the case manager during the process when the therapist or the research team consider it important for my/our child's treatment |  |  |

Information about the scan:

I/we understand that I/we will be informed if abnormal changes are observed in our child's brain and that relevant information, if necessary, may be forwarded to my/our own doctor and/or the relevant hospital department. I/we understand that this examination is not a substitute for a regular examination, as the images will not be systematically assessed by a physician. It is therefore important to carry out a clinical examination should the child have symptoms which make this necessary.

Would you like to receive information about the results of the research project?

Yes (set x) No (set x)

The name or names of the legal guardians:

Date: Signature:

Date: Signature:

**Declaration by the person providing the information:**

I declare that the parents/children have received oral and written information about the trial.

In my opinion the participant has received sufficient information to make an informed decision about participating in the trial.

The name of the person who provided the information:

Date: Signature:

Project identification: Scientific Committees Protocol No: H-18010607

**TO PARENTS OF CONTROLS**

**INFORMED CONSENT FOR PARTICIPATION IN A HEALTH SCIENCE RESEARCH PROJECT**

**Research project title:** TECTO Trial - Treatment Effects of family based Cognitive Therapy in children and adolescents with Obsessive compulsive disorder

**Declaration by the holder of parental responsibility:**

I/we have received written and verbal information and I/we know enough about the purpose, method, advantages and disadvantages to give my/our consent.

I/we know that participation is voluntary and that I/we can withdraw my/our consent at any time without my/our son/daughter losing his/her current or future rights to treatment.

I/we consent to (child's name) to participate in the research project. I/we have received a copy of this consent form and a copy of the written information about the project for my/our own use.

| I/we give consent that, in connection with the research project, there may be | **Yes** set x | **No** set x |
| --- | --- | --- |
| That there is recorded video of my/our child and me/us |  |  |
| That there is recorded audio file of my/our child and me/us |  |  |
| That MR scans are done of my/our child |  |  |
| Saliva samples are taken from my/our child and stored in the research biobank |  |  |

Information about the scan:

I/we understand that I/we will be informed if abnormal changes are observed in our child's brain and that relevant information, if necessary, may be forwarded to my/our own doctor and/or the relevant hospital department. I/we understand that this examination is not a substitute for a regular examination, as the images will not be systematically assessed by a physician. It is therefore important to carry out a clinical examination should the child have symptoms which make this necessary.

Would you like to receive information about the results of the research project?

Yes (set x) No (set x)

The name or names of the legal guardians:

Date: Signature:

Date: Signature:

**Declaration by the person providing the information:**

I declare that the parents/children have received oral and written information about the trial.

In my opinion the participant has received sufficient information to make an informed decision about participating in the trial.

The name of the person who provided the information:

Date: Signature:

Project identification: Scientific Committees Protocol No: H-18010607

**TO AN ADULT CONTROL**

**INFORMED CONSENT FOR PARTICIPATION IN A HEALTH SCIENCE RESEARCH PROJECT**

**Research project title:** TECTO Trial - Treatment Effects of family based Cognitive Therapy in children and adolescents with Obsessive Compulsive Cisorder

**Declaration of the project participant who has reached the age of 18:**

I have received written and verbal information and I know enough about the purpose, method, advantages, and disadvantages to give my consent.

I know that participation is voluntary and that I can always withdraw my consent without losing my current or future rights to treatment.

I consent to) participate in the research project. I have received a copy of this consent form and a copy of the written information about the project for my own use.

| I give my consent that, in connection with the research project, there may be | **Yes** set x | **No** set x |
| --- | --- | --- |
| Recorded video of me |  |  |
| Recorded audio file of me |  |  |
| That MR scans are done of me |  |  |
| Saliva samples are taken from me and stored in a research biobank |  |  |

Information about the scan:

I understand that I will be informed if any abnormal changes are observed in my brain and that relevant information may be passed on to my own doctor and/or the relevant hospital department, if necessary. I understand that this examination is not a substitute for a regular examination, as the images will not be systematically assessed by a physician. It is therefore important to carry out a clinical examination should I have any symptoms that make this necessary.

Would you like to receive information about the results of the research project?

Yes (set x) No (set x)

Name:

Date: Signature:

**Declaration by the person providing the information:**

I declare that the participant has received oral and written information about the trial.

In my opinion the participant has received sufficient information to make an informed decision about participating in the trial.

The name of the person who provided the information:

Date: Signature:

Project identification: Scientific Committees Protocol No: H-18010607

**TO PARENTS OF CONTROLS**

**INFORMED CONSENT FOR PARTICIPATION IN A HEALTH SCIENCE RESEARCH PROJECT**

**Research project title:** TECTO Trial - Treatment Effects of family based Cognitive Therapy in children and adolescents with Obsessive Compulsive Disorder

**Declaration of the project participant:**

I have received written and verbal information and I know enough about the purpose, method, advantages, and disadvantages to give my consent.

I know that participation is voluntary and that I can always withdraw my consent without losing my current or future rights to treatment.

I consent to participate in the research project. I have received a copy of this consent form and a copy of the written information about the project for my own use.

| I give my consent that, in connection with the research project, there may be | **Yes** set x | **No** set x |
| --- | --- | --- |
| Recorded video of me |  |  |
| Saliva samples are taken from me and stored in a research biobank |  |  |

Project Participant:

Date: Signature:

Would you like to receive information about the results of the research project?

Yes (set x) No (set x)

**Declaration by the person providing the information:**

I declare that the participant has received oral and written information about the trial.

In my opinion the participant has received sufficient information to make an informed decision about participating in the trial.

The name of the person who provided the information:

Date: Signature:

Project identification: Scientific Committees Protocol No: H-1801060
