# Supplementary material for: Family-based cognitive behavioural therapy versus family-based relaxation therapy for obsessive-compulsive disorder in children and adolescents: protocol for a randomised clinical trial (the TECTO trial)
Source: BMC Psychiatry. 2022 Mar 19;22:204. doi: 10.1186/s12888-021-03669-2 (PMC8933964; doi:10.1186/s12888-021-03669-2)
Supplement: Supplementary file 5 — Additional file 5. [file 12888_2021_3669_MOESM5_ESM.docx]

**Supplementary file 5:**

**Definitions of adverse events**

Adverse events as defined by the International Conference on Harmonisation of Technical Requirements for Registration of Pharmaceuticals for Human Use – Guidelines for Good Clinical Practice

[*ICH Harmonised Guideline. Clinical safety data management: definitions and standards for expedited reporting E2A*. 1994.]

**Definitions of adverse events**

**Adverse Event:** any undesirable medical event occurring to a participant during a clinical trial, which does not necessarily have a causal relationship with the intervention.

**Adverse Reaction:** any undesirable and unintended medical response related to the intervention occurring to a participant during a clinical trial.

**Serious Adverse Event (SAE):** any adverse event that results in death, is life-threatening, requires hospitalisation or prolongs existing hospitalisation, results in persistent or significant disability or incapacity, or is a congenital anomaly or birth defect, which does not necessarily have a causal relationship with the intervention.

**Serious Adverse Reaction (SAR):** any adverse reaction that results in death, is life-threatening, requires hospitalisation or prolongs existing hospitalisation, results in persistent or significant disability or incapacity, or is a congenital anomaly or birth defect. This response is related to the intervention occurring to a participant during a clinical trial.

**Suspected Unexpected Serious Adverse Reaction (SUSAR):** any suspected adverse reaction which is both serious and unexpected (the nature or severity of which is not consistent with the information available to date).

**Classification of an event**

We will use the classifications in the table below to classify if an event is an ‘adverse event’ or an ‘adverse reaction’ (i.e. whether there is a causal relationship between the intervention and the event). Only events classified as ‘certain’ or ‘probable/likely’ will be classified as ‘reactions’, all others will be classified as ‘events’.

**Table: Classification of causality of adverse reactions.**

| **Certain** | - Event or laboratory test abnormality, with plausible time relationship to the trial intervention; cannot be explained by disease or other treatments. - Response to withdrawal plausible (pharmacologically, pathologically). - Event definitive pharmacologically or phenomenologically (i.e. an objective and specific medical disorder or a recognised pharmacological phenomenon). - Re-challenge satisfactory, if necessary. |
| --- | --- |
| **Probable / likely** | - Event or laboratory test abnormality, with reasonable time relationship to the trial intervention. - Unlikely to be attributed to disease or other treatment; response to withdrawal clinically reasonable. - Rechallenge not required. |
| **Possible** | - Event or laboratory test abnormality, with reasonable time relationship to the trial intervention. - Could also be explained by disease or other treatments. - Information on drug withdrawal may be lacking or unclear. |
| **Unlikely** | - Event or laboratory test abnormality, with a time to the trial intervention that makes a relationship improbable (but not impossible). - Disease or other drugs provide plausible explanations. |
| **Conditional/ unclassified** | - Event or laboratory test abnormality. - More data for proper assessment needed, or - Additional data under examination. |
| **Un-assessable/ unclassifiable** | - Report suggesting an adverse reaction. - Cannot be judged because information is insufficient or contradictory. - Data cannot be supplemented or verified. |

**Reporting**

SAEs, SARs, and SUSARs will be reported to the regional ethics committee in an expedited manner, and at the latest 7 days after the sponsor has received information about the event. SAEs, SARs, SUSARs will further be reported to the regional ethics committee in a yearly safety report.

All SAEs (including SARs and SUSARs) will also be reported in the trial as an outcome. The NEQ that assesses negative effects related to the intervention will also be reported as an outcome in the trial.

Furthermore, the following definitions will be used:

[Linden, M., *How to define, find and classify side effects in psychotherapy: from unwanted events to adverse treatment reactions.* Clinical psychology & psychotherapy, 2013. **20**(4): p. 286-296].

**Unwanted/adverse events (UE)**: all negative events that occur in parallel or in wake of treatment.

**Treatment-emergent reactions:** Any UE that is evaluated to be caused by treatment.

**Adverse treatment reactions:** Any UE that is probably caused by correct treatment.

**Malpractice reaction:** Any UE that is probably caused by incorrect or improperly applied treatment.
