# Supplementary material for: Family-based cognitive behavioural therapy versus family-based relaxation therapy for obsessive-compulsive disorder in children and adolescents: protocol for a randomised clinical trial (the TECTO trial)
Source: BMC Psychiatry. 2022 Mar 19;22:204. doi: 10.1186/s12888-021-03669-2 (PMC8933964; doi:10.1186/s12888-021-03669-2)
Supplement: Supplementary file 6 — Additional file 6. [file 12888_2021_3669_MOESM6_ESM.pdf]

Treatment Fidelity – Psychoeducation and Relaxation Training (PRT)

Treatment integrity assessment form: Session 1

Patient ID: ___________ Therapist: ________________ Date of scoring: ___________ Assessed by: _____________

PERFORMANCE: Assessment of compliance with manual/treatment model

Use the following scale:

1	2	3	4
Very deficient	Lack of compliance	Good compliance	Very good compliance

1. **Contact** **Score: ___**
   - Knowledge of the child's current hobbies and strengths __
   - Focus on alliance building __

2. **Obtains patient knowledge** **Score: ___**
   - Overview of social, developmental and school conditions and history __
   - Obtain knowledge about obsessive-compulsive symptoms (OCD) symptoms and treatment history __
   - Obtain knowledge about the impact of symptoms on everyday life __

3. **Psychoeducation about OCD** **Score: ___**
   - Explores family knowledge of OCD __
   - Provides factual information about OCD __
   - Introduces the understanding of OCD as a neurobiological disorder __
   - Introduces the idea that the patient may live an ordinary life despite OCD __

4. **Psychoeducation about treatment** **Score: ___**
   - Description of the relaxation therapy __
   - Introduces the rationale for relaxation as a treatment approach __
   - Stresses the importance of practicing relaxation __
   - Description of a behavioral reward program __

5. **Introduce home assignments** **Score: ___**
   - Self-monitoring of symptoms in diary __
   - Implementation of a behavioral reward program __

Session 1

- **Overall assessment - compliance with session 1 manual:** **Score: ___**
  The score should be an overall assessment of compliance related to the objectives and core areas of the manual for the whole session

6. **Parental Involvement:** **Score: ___**
   - One or both parents present throughout the session __

COMPETENCE: Assessment of competence to administer manual

Use the following scale: (tick)

Manages the protocol with skill, is able to explain and implement			
**1**<br>Very poor competence	**2**<br>Poor competence	**3**<br>Good competence	**4**<br>Very good competence

RELATIONSHIP AND PROCESS SKILLS: Therapist competence in relationship and process skills

Use the following scale: (tick)

Demonstrates flexibility, sensitivity, creativity and good communication skills			
**1**<br>Very poor competence	**2**<br>Poor competence	**3**<br>Good competence	**4**<br>Very good competence

Session 1

COMMENTS ON SESSION 1:

1. **What is your assessment of how difficult this client and family were?**

1	2	3	4
Very difficult	Difficult	Not difficult	On the whole not difficult

2. **Was there a video recording of the entire session?**

Yes __
No __

3. **Was the recording difficult to score due to poor technical quality?**

Yes __
No __

4. **Other comments** (if you have other comments, please note them in the box)

Treatment integrity assessment form: Session 2

Patient ID: ___________ Therapist: ________________ Date of scoring: ___________ Assessed by: _____________

PERFORMANCE: Assessment of compliance with manual/treatment model

Use the following scale:

1	2	3	4
Very deficient	Lack of compliance	Good compliance	Very good compliance

1. **Review events from last week** **Score: ___**
   - Events in the child's life and environment __
   - OCD symptoms and impact on everyday life __
2. **Psychoeducation** **Score: ___**
   - Establishes that patient remembers definition of OCD from session 1 __
3. **Introduction of new tools by the therapist** **Score: ___**
   - Explains feelings thermometer __
   - Makes symptom hierarchy __
   - Explains the rationale behind the symptom hierarchy __
4. **Homework** **Score: ___**
   - Reviews homework from last week __
   - Provides home assignments: self-monitoring with feelings thermometer __

Session 2

- **Overall assessment - compliance with session 2 manual:** **Score: ___**
  The score should be an overall assessment of compliance related to the objectives and core areas of the manual for the whole session

5. **Parental Involvement:** **Score: ___**
   - One or both parents present throughout the session __
   - Parent theme: Expectations __

COMPETENCE: Assessment of competence to administer manual

Use the following scale: (tick)

Manages the protocol with skill, able to explain and implement			
**1**<br>Very poor competence	**2**<br>Poor competence	**3**<br>Good competence	**4**<br>Very good competence

RELATIONSHIP AND PROCESS SKILLS: Therapist competence in relationship and process skills

Use the following scale: (tick)

Demonstrates flexibility, sensitivity, creativity and good communication skills			
**1**<br>Very poor competence	**2**<br>Poor competence	**3**<br>Good competence	**4**<br>Very good competence

Session 2

COMMENTS ON SESSION 2:

1. **What is your assessment of how difficult this client and family were?**

1	2	3	4
Very difficult	Difficult	Not difficult	On the whole not difficult

2. **Was there a video recording of the entire session?**

Yes __
No __

3. **Was the recording difficult to score due to poor technical quality?**

Yes __
No __

4. **Other comments** (if you have other comments, please note them in the box)

Treatment integrity assessment form: Session 3

Patient ID: ___________ Therapist: ________________ Date of scoring: ___________ Assessed by: _____________

PERFORMANCE: assessment of compliance with manual/treatment model

Use the following scale:

1	2	3	4
Very deficient	Lack of compliance	Good compliance	Very good compliance

1. Review events from last week **Score: ___**
- Events in the child's life and environment __
- OCD symptoms and impact on everyday life __

2. Rating of OCD symptoms **Score: ___**
- Rates symptoms on the feelings thermometer __

3. Relaxation training **Score: ___**
- Repeat rationale behind relaxation training __
- Explain muscle tension and relaxation exercises __
- The patient must sit in a chair or lie down __
- Performing an exercise __
- Focus on the parts of the body that the child has difficulty relaxing __

4. Homework **Score: ___**
- Reviews homework from last week __
- New homework: Practice exercise daily __
- New homework: Monitor exercises __

Session 3

- **Overall assessment - compliance with session 3 manual:** **Score: ___**
  The score should be an overall assessment of compliance related to the objectives and core areas of the manual for the whole session

5. **Parental Involvement:** **Score: ___**
   - One or both parents present for the last 30 minutes of the session __
   - Reviews the content of the session with parents __
   - Explains homework __
   - Parent theme: Exercise with parents __

COMPETENCE: Assessment of competence for manual administration

Use the following scale: (tick)

Manages the protocol with skill, able to explain and implement			
**1**<br>Very poor competence	**2**<br>Poor competence	**3**<br>Good competence	**4**<br>Very good competence

RELATIONSHIP AND PROCESS SKILLS: Therapist competence in relationship and process skills

Use the following scale: (tick)

Demonstrates flexibility, sensitivity, creativity and good communication skills			
**1**<br>Very poor competence	**2**<br>Poor competence	**3**<br>Good competence	**4**<br>Very good competence

Session 3

COMMENTS ON SESSION 3:

1. **What is your assessment of how difficult this client and family were?**

1	2	3	4
Very difficult	Difficult	Not difficult	On the whole not difficult

2. **Was there a video recording of the entire session?**

Yes __
No __

3. **Was the recording difficult to score due to poor technical quality?**

Yes __
No __

4. **Other comments** (if you have other comments, please note them in the box)

Treatment integrity assessment form: Session 4

Patient ID: ___________ Therapist: ________________ Date of scoring: ___________ Assessed by: _____________

PERFORMANCE: Assessment of compliance with manual/treatment model

Use the following scale:

1	2	3	4
Very deficient	Lack of compliance	Good compliance	Very good compliance

1. **Review events from last week** **Score: ___**
   - Events in the child's life and environment __
   - OCD symptoms and impact on everyday life __
   - Talk about positive experiences during the week __

2. **Rating of OCD symptoms** **Score: ___**
   - Makes rating on the feelings thermometer for each symptom __

3. **Relaxation training** **Score: ___**
   - Does an exercise from the catalogue __

4. **Homework** **Score: ___**
   - Reviews homework from last week __
   - New homework assignment: Practice exercise(s) daily __
   - New homework: Monitor exercises __

Session 4

- **Overall assessment - compliance with session 4 manual:** **Score: ___**
  The score should be an overall assessment of compliance related to the objectives and core areas of the manual for the whole session

5. Parental Involvement: **Score: ___**
- One or both parents present for the last 30 minutes __
- Reviews the content of the session with parents __
- Explains homework __
- Parent theme: Discuss good conditions for practicing at home __

COMPETENCE: Assessment of competence to administer manual

Use the following scale: (tick)

Manages the protocol with skill, able to explain and implement			
**1**<br>Very poor competence	**2**<br>Poor competence	**3**<br>Good competence	**4**<br>Very good competence

RELATIONSHIP AND PROCESS SKILLS: Therapist competence in relationship and process skills

Use the following scale: (tick)

Demonstrates flexibility, sensitivity, creativity and good communication skills			
**1**<br>Very poor competence	**2**<br>Poor competence	**3**<br>Good competence	**4**<br>Very good competence

Session 4

COMMENTS ON SESSION 4:

1. **What is your assessment of how difficult this client and family were?**

1	2	3	4
Very difficult	Difficult	Not difficult	On the whole not difficult

2. **Was there a video recording of the entire session?**

Yes __
No __

3. **Was the recording difficult to score due to poor technical quality?**

Yes __
No __

4. **Other comments** (if you have other comments, please note them in the box)

Treatment integrity assessment form: Session 5

Patient ID: ___________ Therapist: ________________ Date of scoring: ___________ Assessed by: _____________

PERFORMANCE: assessment of compliance with manual/treatment model

Use the following scale:

1	2	3	4
Very deficient	Lack of compliance	Good compliance	Very good compliance

1. **Review events from last week** **Score: ___**
   - Events in the child's life and environment __
   - OCD symptoms and impact on everyday life __
   - Talk about positive experiences __

2. **Rating of OCD symptoms** **Score: ___**
   - Makes rating on the feelings thermometer for each symptom __

3. **Relaxation training** **Score: ___**
   - Does an exercise from the catalogue (relaxation or breathing) __
   - Talking about relaxation in different situations __

4. **Homework** **Score: ___**
   - Reviewing homework from last week __
   - New homework: Do exercise(s) daily __
   - New homework: Monitor training __

Session 5

- **Overall assessment - compliance with session 5 manual:** **Score: ___**
  The score should be an overall assessment of compliance related to the objectives and core areas of the manual for the whole session

5. **Parental Involvement:** **Score: ___**
   - One or both parents present for the last 30 minutes __
   - Review the content of the session with parents __
   - Explains homework __
   - Parent theme: Minimizing guilt __

COMPETENCE: Assessment of competence to administer manual

Use the following scale: (tick)

Manages the protocol with skill, able to explain and implement			
**1**<br>Very poor competence	**2**<br>Poor competence	**3**<br>Good competence	**4**<br>Very good competence

RELATIONSHIP AND PROCESS SKILLS: Therapist competence in relationship and process skills

Use the following scale: (tick)

Demonstrates flexibility, sensitivity, creativity and good communication skills			
**1**<br>Very poor competence	**2**<br>Poor competence	**3**<br>Good competence	**4**<br>Very good competence

Session 5

COMMENTS ON SESSION 5:

1. **What is your assessment of how difficult this client and family were?**

1	2	3	4
Very difficult	Difficult	Not difficult	On the whole not difficult

2. **Was there a video recording of the entire session?**

   Yes __
   No __

3. **Was the recording difficult to score due to poor technical quality?**

   Yes __
   No __

4. **Other comments** (if you have other comments, please note them in the box)

Treatment integrity assessment form: Session 6

Patient ID: ___________ Therapist: ________________ Date of scoring: ___________ Assessed by: _____________

PERFORMANCE: Assessment of compliance with manual/treatment model

Use the following scale:

1	2	3	4
Very deficient	Lack of compliance	Good compliance	Very good compliance

1. **Review events from last week** **Score: ___**
   - Events in the child's life and environment __
   - OCD symptoms and impact on everyday life __
   - Talk about positive experiences __

2. **Rating of OCD symptoms** **Score: ___**
   - Makes rating on the feelings thermometer for each symptom __

3. **Relaxation training** **Score: ___**
   - Does an exercise from the catalogue __
   - Talks about relaxation in at least one new situation __

4. **Homework** **Score: ___**
   - Reviews homework from last week __
   - New homework: Do exercises daily __
   - New homework: Monitor training __

Session 6

- **Overall assessment - compliance with session 6 manual:** **Score: ___**
  The score should be an overall assessment of compliance related to the objectives and core areas of the manual for the whole session

5. **Parental Involvement:** **Score: ___**
   - One or both parents present for the last 30 minutes __
   - Reviews the content of the session with parents __
   - Explains homework __
   - Parent theme: The family's attitude towards the child and the OCD __

COMPETENCE: Assessment of competence to administer manual

Use the following scale: (tick)

Manages the protocol with skill, able to explain and implement			
**1**<br>Very poor competence	**2**<br>Poor competence	**3**<br>Good competence	**4**<br>Very good competence

RELATIONSHIP AND PROCESS SKILLS: Therapist competence in relationship and process skills

Use the following scale: (tick)

Demonstrates flexibility, sensitivity, creativity and good communication skills			
**1**<br>Very poor competence	**2**<br>Poor competence	**3**<br>Good competence	**4**<br>Very good competence

Session 6

COMMENTS ON SESSION 6:

1. **What is your assessment of how difficult this client and family were?**

1	2	3	4
Very difficult	Difficult	Not difficult	On the whole not difficult

2. **Was there a video recording of the entire session?**

   Yes __
   No __

3. **Was the recording difficult to score due to poor technical quality?**

   Yes __
   No __

4. **Other comments** (if you have other comments, please note them in the box)

Treatment integrity assessment form: Session 7

Patient ID: ___________ Therapist: ________________ Date of scoring: ___________ Assessed by: _____________

PERFORMANCE: Assessment of compliance with manual/treatment model

Use the following scale:

1	2	3	4
Very deficient	Lack of compliance	Good compliance	Very good compliance

1. **Review events from last week** **Score: ___**
   - Events in the child's life and environment __
   - OCD symptoms and impact on everyday life __
   - Talk about positive experiences __

2. **Rating of OCD symptoms** **Score: ___**
   - Makes rating on the feelings thermometer for each symptom __

3. **Relaxation training** **Score: ___**
   - Does an exercise from the catalogue (relaxation or breathing) __
   - Talks about relaxation in at least one new situation __
   -

4. **Homework** **Score: ___**
   - Reviews homework from last week __
   - New homework: Do exercises daily __
   - New homework: Monitor training __

Session 7

- **Overall assessment - compliance with session 7 manual:** **Score: ___**
  The score should be an overall assessment of compliance related to the objectives and core areas of the manual for the whole session

5. Parental Involvement: **Score: ___**
- One or both parents present throughout the session __

COMPETENCE: Assessment of competence to administer manual

Use the following scale: (tick)

Manages the protocol with skill, able to explain and implement			
**1**<br>Very poor competence	**2**<br>Poor competence	**3**<br>Good competence	**4**<br>Very good competence

RELATIONSHIP AND PROCESS SKILLS: Therapist competence in relationship and process skills

Use the following scale: (tick)

Demonstrates flexibility, sensitivity, creativity and good communication skills			
**1**<br>Very poor competence	**2**<br>Poor competence	**3**<br>Good competence	**4**<br>Very good competence

Session 7

COMMENTS ON SESSION 7:

1. **What is your assessment of how difficult this client and family were?**

1	2	3	4
Very difficult	Difficult	Not difficult	On the whole not difficult

2. **Was there a video recording of the entire session?**

Yes __
No __

3. **Was the recording difficult to score due to poor technical quality?**

Yes __
No __

4. **Other comments** (if you have other comments, please note them in the box)

Treatment integrity assessment form: Session 8

Patient ID: ___________ Therapist: ________________ Date of scoring: ___________ Assessed by: _____________

PERFORMANCE: Assessment of compliance with manual/treatment model

Use the following scale:

1	2	3	4
Very deficient	Lack of compliance	Good compliance	Very good compliance

1. **Review events from last week** **Score: ___**
   - Events in the child's life and environment __
   - OCD symptoms and impact on everyday life __
   - Talk about positive experiences __

2. **Rating of OCD symptoms** **Score: ___**
   - Makes rating on the feelings thermometer for each symptom __

3. **Relaxation training** **Score: ___**
   - Does an exercise from the catalogue (relaxation or breathing) __
   - Talks about relaxation in at least one new situation __

4. **Homework** **Score: ___**
   - Reviews homework from last week __
   - New homework: Do exercises daily __
   - New homework: Monitor training __

Session 8

- **Overall assessment - compliance with session 8 manual:** **Score: ___**
  The score should be an overall assessment of compliance related to the objectives and core areas of the manual for the whole session

5. **Parental Involvement:** **Score: ___**
   - One or both parents present for the last 30 minutes __
   - Reviews the content of the session with parents __
   - Explains homework __
   - Parent theme: Child taking responsibility for the treatment __

COMPETENCE: Assessment of competence to administer manual

Use the following scale: (tick)

Manages the protocol with skill, able to explain and implement			
**1**<br>Very poor competence	**2**<br>Poor competence	**3**<br>Good competence	**4**<br>Very good competence

RELATIONSHIP AND PROCESS SKILLS: Therapist competence in relationship and process skills

Use the following scale: (tick)

Demonstrates flexibility, sensitivity, creativity and good communication skills			
**1**<br>Very poor competence	**2**<br>Poor competence	**3**<br>Good competence	**4**<br>Very good competence

Session 8

COMMENTS ON SESSION 8:

1. **What is your assessment of how difficult this client and family were?**

1	2	3	4
Very difficult	Difficult	Not difficult	On the whole not difficult

2. **Was there a video recording of the entire session?**

Yes __
No __

3. **Was the recording difficult to score due to poor technical quality?**

Yes __
No __

4. **Other comments** (if you have other comments, please note them in the box)

Treatment integrity assessment form: Session 9

Patient ID: ___________ Therapist: ________________ Date of scoring: ___________ Assessed by: _____________

PERFORMANCE: Assessment of compliance with manual/treatment model

Use the following scale:

1	2	3	4
Very deficient	Lack of compliance	Good compliance	Very good compliance

1. **Review events from last week** **Score: ___**
   - Events in the child's life and environment __
   - OCD symptoms and impact on everyday life __
   - Talk about positive experiences __

2. **Rating of OCD symptoms** **Score: ___**
   - Makes rating on the feelings thermometer for each symptom __

3. **Relaxation training** **Score: ___**
   - Does an exercise from the catalogue (relaxation or breathing) __

4. **Homework** **Score: ___**
   - Reviews homework from last week __
   - New homework: Do exercises daily __
   - New homework: Monitor training __

Session 9

- **Overall assessment - compliance with session 9 manual:** **Score: ___**
  The score should be an overall assessment of compliance related to the objectives and core areas of the manual for the whole session

5. **Parental Involvement:** **Score: ___**
   - One or both parents present for the last 30 minutes __
   - Reviews the content of the session with parents __
   - Explains homework __
   - Parent theme: Distinguishing OCD from other problems __

COMPETENCE: Assessment of competence to administer manual

Use the following scale: (tick)

Manages the protocol with skill, able to explain and implement			
**1**<br>Very poor competence	**2**<br>Poor competence	**3**<br>Good competence	**4**<br>Very good competence

RELATIONSHIP AND PROCESS SKILLS: Therapist competence in relationship and process skills

Use the following scale: (tick)

Demonstrates flexibility, sensitivity, creativity and good communication skills			
**1**<br>Very poor competence	**2**<br>Poor competence	**3**<br>Good competence	**4**<br>Very good competence

Session 9

COMMENTS ON SESSION 9:

1. **What is your assessment of how difficult this client and family were?**

1	2	3	4
Very difficult	Difficult	Not difficult	On the whole not difficult

2. **Was there a video recording of the entire session?**

Yes __
No __

3. **Was the recording difficult to score due to poor technical quality?**

Yes __
No __

4. **Other comments** (if you have other comments, please note them in the box)

Treatment integrity assessment form: Session 10

Patient ID: ___________ Therapist: ________________ Date of scoring: ___________ Assessed by: _____________

PERFORMANCE: Assessment of compliance with manual/treatment model

Use the following scale:

1	2	3	4
Very deficient	Lack of compliance	Good compliance	Very good compliance

1. **Review events from last week** **Score: ___**
   - Events in the child's life and environment __
   - OCD symptoms and impact on everyday life __
   - Talk about positive experiences __

2. **Rating of OCD symptoms** **Score: ___**
   - Makes rating on the feelings thermometer for each symptom __

3. **Relaxation training** **Score: ___**
   - Does an exercise from the catalogue (relaxation or breathing) __
   -

4. **Homework** **Score: ___**
   - Reviewing homework from last week __
   - New homework: Do exercises daily __
   - New homework: Monitor training __

Session 10

- **Overall assessment - compliance with session 10 manual:** **Score: ___**
  The score should be an overall assessment of compliance related to the objectives and core areas of the manual for the whole session

5. **Parental Involvement:** **Score: ___**
   - One or both parents present for the last 30 minutes __
   - Review the content of the session with parents __
   - Explain homework __
   - Parent theme: Nurturing the family __

COMPETENCE: Assessment of competence to administer manual

Use the following scale: (tick)

Manages the protocol with skill, able to explain and implement			
**1**<br>Very poor competence	**2**<br>Poor competence	**3**<br>Good competence	**4**<br>Very good competence

RELATIONSHIP AND PROCESS SKILLS: Therapist competence in relationship and process skills

Use the following scale: (tick)

Demonstrates flexibility, sensitivity, creativity and good communication skills			
**1**<br>Very poor competence	**2**<br>Poor competence	**3**<br>Good competence	**4**<br>Very good competence

Session 10

COMMENTS ON SESSION 10:

1. **What is your assessment of how difficult this client and family were?**

1	2	3	4
Very difficult	Difficult	Not difficult	On the whole not difficult

2. **Was there a video recording of the entire session?**

Yes __
No __

3. **Was the recording difficult to score due to poor technical quality?**

Yes __
No __

4. **Other comments** (if you have other comments, please note them in the box)

Treatment integrity assessment form: Session 11

Patient ID: ___________ Therapist: ________________ Date of scoring: ___________ Assessed by: _____________

PERFORMANCE: Assessment of compliance with manual/treatment model

Use the following scale:

1		2	3	4
Very deficient	Lack of compliance	Good compliance	Very good compliance	

1. **Review events from last week** **Score: ___**
   - Events in the child's life and environment __
   - OCD symptoms and impact on everyday life __
   - Talk about positive experiences __

2. **Rating of OCD symptoms** **Score: ___**
   - Makes rating on the feelings thermometer for each symptom __

3. **Relaxation training** **Score: ___**
   - Does an exercise from the catalogue (relaxation or breathing) __
   -

4. **Homework** **Score: ___**
   - Reviewing homework from last week __
   - New homework: Do exercises daily __
   - New homework: Monitor training __

Session 11

- **Overall assessment - compliance with session 11 manual:** **Score: ___**
  The score should be an overall assessment of compliance related to the objectives and core areas of the manual for the whole session

5. **Parental Involvement:** **Score: ___ __**
   - Parents present throughout the session

COMPETENCE: Assessment of competence to administer manual

Use the following scale: (tick)

Manages the protocol with skill, able to explain and implement			
**1**<br>Very poor competence	**2**<br>Poor competence	**3**<br>Good competence	**4**<br>Very good competence

RELATIONSHIP AND PROCESS SKILLS: Therapist competence in relationship and process skills

Use the following scale: (tick)

Demonstrates flexibility, sensitivity, creativity and good communication skills			
**1**<br>Very poor competence	**2**<br>Poor competence	**3**<br>Good competence	**4**<br>Very good competence

Session 11

COMMENTS ON SESSION 11:

1. **What is your assessment of how difficult this client and family were?**

1	2	3	4
Very difficult	Difficult	Not difficult	On the whole not difficult

2. **Was there a video recording of the entire session?**

Yes __
No __

3. **Was the recording difficult to score due to poor technical quality?**

Yes __
No __

4. **Other comments** (if you have other comments, please note them in the box)

Treatment integrity assessment form: Session 12

Patient ID:______________ Therapist: ________________ Date of scoring:_________ Assessed by: ______________

PERFORMANCE: Assessment of compliance with manual/treatment model

Use the following scale:

1	2	3	4
Very deficient	Lack of compliance	Good compliance	Very good compliance

1. **Review events from last week** **Score: ___**
   - Events in the child's life and environment __
   - OCD symptoms and impact on everyday life __
   - Talk about positive experiences __

2. **Rating of OCD symptoms** **Score: ___**
   - Makes rating on the feelings thermometer for each symptom __

3. **Relaxation training** **Score: ___**
   - Does an exercise from the catalogue (relaxation or breathing) __

4. **Homework** **Score: ___**
   - Reviewing homework from last week __
   - New homework: Do exercises daily __
   - New homework: Monitor training __

Session 12

Overall assessment - compliance with session 12 manual: **Score: ___**
The score should be an overall assessment of compliance related to the objectives and core areas of the manual for the whole session

5. **Parental Involvement:** **Score: ___**
   - One or both parents present for the last 30 minutes __
   - Reviews the content of the session with parents __
   - Explains homework __
   - Parent theme: Preventing relapse __

COMPETENCE: Assessment of competence to administer manual

Use the following scale: (tick)

Manages the protocol with skill, able to explain and implement			
**1**<br>Very poor competence	**2**<br>Poor competence	**3**<br>Good competence	**4**<br>Very good competence

RELATIONSHIP AND PROCESS SKILLS: Therapist competence in relationship and process skills

Use the following scale: (tick)

Demonstrates flexibility, sensitivity, creativity and good communication skills			
**1**<br>Very poor competence	**2**<br>Poor competence	**3**<br>Good competence	**4**<br>Very good competence

Session 12

COMMENTS ON SESSION 12:

1. **What is your assessment of how difficult this client and family were?**

1	2	3	4
Very difficult	Difficult	Not difficult	On the whole not difficult

2. **Was there a video recording of the entire session?**

Yes __
No __

3. **Was the recording difficult to score due to poor technical quality?**

Yes __
No __

4. **Other comments** (if you have other comments, please note them in the box)

Treatment integrity assessment form: Session 13

Patient ID: ______________ Therapist: ________________ Date of scoring:_________ Assessed by: _______________

PERFORMANCE: Assessment of compliance with manual/treatment model

Use the following scale:

1	2	3	4
Very deficient	Lack of compliance	Good compliance	Very good compliance

1. **Review events from last week** **Score: ___**
   - Events in the child's life and environment __
   - OCD symptoms and impact on everyday life __
   - Talk about positive experiences __

2. **Rating of OCD symptoms** **Score: ___**
   - Makes rating on the feelings thermometer for each symptom __

3. **Relaxation training** **Score: ___**
   - Does an exercise from the catalogue (relaxation or breathing) __
   -

4. **Homework** **Score: ___**
   - Reviewing homework from last week __
   - New homework: Do exercises daily __
   - New homework: Monitor training __

Session 13

Overall assessment - compliance with session 12 manual: **Score: ___**
The score should be an overall assessment of compliance related to the objectives and core areas of the manual for the whole session

5. **Parental Involvement:** **Score: ___**
   - One or both parents present for the last 30 minutes __
   - Reviews the content of the session with parents __
   - Explains homework __
   - Parent theme: Preventing relapse __

COMPETENCE: Assessment of competence to administer manual

Use the following scale: (tick)

Manages the protocol with skill, able to explain and implement			
**1**<br>Very poor competence	**2**<br>Poor competence	**3**<br>Good competence	**4**<br>Very good competence

RELATIONSHIP AND PROCESS SKILLS: Therapist competence in relationship and process skills

Use the following scale: (tick)

Demonstrates flexibility, sensitivity, creativity and good communication skills			
**1**<br>Very poor competence	**2**<br>Poor competence	**3**<br>Good competence	**4**<br>Very good competence

Session 13

COMMENTS ON SESSION 12:

1. **What is your assessment of how difficult this client and family were?**

1	2	3	4
Very difficult	Difficult	Not difficult	On the whole not difficult

2. **Was there a video recording of the entire session?**

   Yes __
   No __

3. **Was the recording difficult to score due to poor technical quality?**

   Yes __
   No __

4. **Other comments** (if you have other comments, please note them in the box)

Treatment integrity assessment form: Session 14

Patient ID: ______________ Therapist: ________________ Date of scoring:_________ Assessed by: _______________

PERFORMANCE: Assessment of compliance with manual/treatment model

Use the following scale:

1	2	3	4
Very deficient	Lack of compliance	Good compliance	Very good compliance

1. **Review events from last week** **Score: ___**
   - Events in the child's life and environment __
   - OCD symptoms and impact on everyday life __
   - Talk about positive experiences __

2. **Rating of OCD symptoms** **Score: ___**
   - Makes rating on the feelings thermometer for each symptom __

3. **Relaxation training** **Score: ___**
   - Does an exercise from the catalogue (relaxation or breathing) __

4. **Homework** **Score: ___**
   - Reviewing homework from last week __

Session 14

- **Overall assessment - compliance with session 14 manual:** **Score: ___**
  The score should be an overall assessment of compliance related to the objectives and core areas of the manual for the whole session

5. Parental Involvement: **Score: __**

- One or both parents are present throughout the session __
- Whole family: Progress figures, relapse prediction, continued training __
- Any further treatment __
- Celebration of completion __

COMPETENCE: Assessment of competence to administer manual

Use the following scale: (tick)

Manages the protocol with skill, able to explain and implement			
**1**<br>Very poor competence	**2**<br>Poor competence	**3**<br>Good competence	**4**<br>Very good competence

RELATIONSHIP AND PROCESS SKILLS: Therapist competence in relationship and process skills

Use the following scale: (tick)

Demonstrates flexibility, sensitivity, creativity and good communication skills			
**1**<br>Very poor competence	**2**<br>Poor competence	**3**<br>Good competence	**4**<br>Very good competence

Session 14

COMMENTS ON SESSION 14:

1. **What is your assessment of how difficult this client and family were?**

1	2	3	4
Very difficult	Difficult	Not difficult	On the whole not difficult

2. **Was there a video recording of the entire session?**

Yes __
No __

3. **Was the recording difficult to score due to poor technical quality?**

Yes __
No __

4. **Other comments** (if you have other comments, please note them in the box)
